# New Se‐Compounds With Antileishmanial, Antitumor, and Carbonic Anhydrase Inhibitory Properties

**DOI:** 10.1002/ardp.70145

**Published:** 2025-12-03

**Authors:** Cristina Morán‐Serradilla, Daniel Plano, Andrea Angelli, Arun K. Sharma, Carmen Sanmartin, Claudiu T. Supuran

**Affiliations:** ^1^ Department of Pharmaceutical Sciences University of Navarra Pamplona Spain; ^2^ Department of NEUROFARBA, Pharmaceutical and Nutraceutical Section University of Firenze Florence Italy; ^3^ Department of Molecular and Precision Medicine Penn State College of Medicine Hershey Pennsylvania USA

**Keywords:** cancer, carbonic anhydrase, leishmaniasis, selenium

## Abstract

In the pursuit of novel agents for treating leishmaniasis and cancer, we have synthesized a small library of phenylcarboxamide‐selenium analogs and evaluated their in vitro antileishmanial, anticancer, and carbonic anhydrase inhibitory activities. The two trifluoromethoxy‐substituted aniline derivatives (**3** and **6**) exhibited IC_50_ values in the low micromolar range in both *Leishmania major* and *Leishmania infantum* promastigotes and presented better selectivity indexes (SIs) than the reference drugs (miltefosine and paromomycin). Furthermore, all of the reported compounds displayed an outstanding antitumoral activity against a panel of 60 cancer cell lines of the National Cancer Institute's (NCI) Developmental Therapeutic Program (DTP). The cytotoxicity of compounds **1**–**3** in nonmalignant HaCaT cells was also evaluated. Furthermore, as several selenocompounds have previously been proven to inhibit tumor‐associated human carbonic anhydrase (hCA) isoforms, all the compounds were assessed against hCA I, II, IX, and XII. Derivative **6** stood out as it inhibited tumor‐associated isoform XII and the cytosolic hCA II isoform in the low micromolar range.

## Graphical abstract

A small library of selenocompounds was evaluated for their antileishmanial, antitumoral, and human carbonic anhydrase (hCA) inhibitory properties. Two trifluoromethoxy‐substituted anilide derivatives (**3** and **6**) were the most effective ones against *Leishmania major* (*L. major*) and *Leishmania infantum* (*L. infantum*) promastigotes and also showed a promising anticancer activity against the Developmental Therapeutic Program (DTP) of the National Cancer Institute (NCI). Besides, they displayed the highest inhibitory activity toward the tumor‐associated hCA XII.

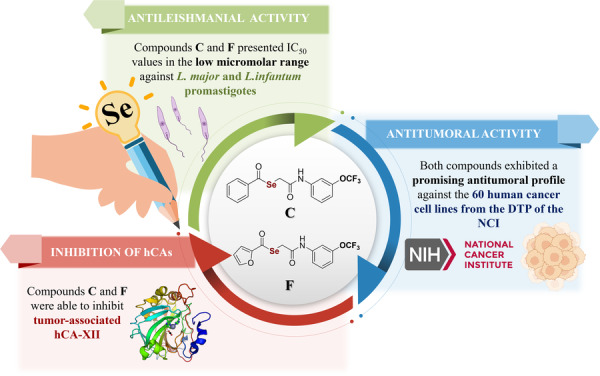

## 1 Introduction

The existing research data indicate that cancer is still a major malignant disorder that afflicts millions of people all around the world each year. Due to its high incidence and increasing resistance to the current therapeutic options, the development of novel, safe, and effective antitumoral compounds continues to be a hot research topic and a global drug discovery challenge. In this line, human carbonic anhydrase (hCA) inhibitors have gained great interest in the past few years. The CA is a ubiquitous family of zinc‐containing enzymes, present in prokaryotes and eukaryotes, which catalyze the reversible hydration of CO_2_ into bicarbonate and a proton (H^+^) with water. In humans, they can be found in several tissues (e.g., kidneys, gastrointestinal tract, eyes, lungs, nervous system, and skin) and different subcellular localizations, where they play an important role in many biological processes (e.g., acid–base balance and gluconeogenesis). Depending on their cellular location, four subgroups can be identified: (i) cytosolic isoforms (CA‐I, CA‐II, CA‐III, CA‐VII, and CA‐XIII); (ii) membrane‐bound isoforms (CA‐IV, CA‐IX, CA‐XII, CA‐XIV, and CA‐XV); (iii) mitochondrial isoforms (CA‐VA and CA‐VB); and (iv) secreted isoforms (CA‐VI) [1, 2]. A great number of these isozymes are considered important therapeutic targets with the potential to be modulated for the treatment of numerous diseases such as neurological disorders, osteoporosis, hypertension, and glaucoma [3, 4]. Of note, their antitumoral application has also been investigated. Many tumor types exhibit low levels of oxygen (hypoxia), increased glucose metabolism, and a dysregulated acid–base balance, with the extracellular pH more acidic than the normal values, which is detrimental to cell survival [5]. However, several cancer types, mainly hypoxic tumors, have overexpressed CA membrane‐associated isoenzymes, hCA‐IX and hCA‐XII, that are known to play a pivotal role in microenvironment *p*H regulation [6, 7, 8, 9]. Both isoforms are implicated in tumor growth, metastasis, and progression, specifically mitigating the intracellular acid stress caused by the increased metabolism in cancer cells [10, 11]. The inhibition of these two enzymes has been shown to impair the growth of the primary tumors and metastases as well as diminishing the population of cancer stem cells [12]. Mounting evidence has revealed that hCA‐IX expression is restricted to a limited number of normal tissues, whereas it is overexpressed on the cell surface of several solid tumors, particularly in clear renal cell carcinoma (RCC), and is associated with the development of tumor hypoxia. In fact, hCA‐IX is highly induced in a hypoxia‐inducible factor‐1 (HIF‐1) dependent way and is constitutively expressed in von Hippel‐Lindau (VHL)–defective cells. Additionally, it has been reported that its presence in breast malignancies is considered a poor prognostic marker [10, 13, 14, 15]. Likewise, hCA‐XII is overexpressed in a wide array of human cancers, such as brain, ovarian, and breast cancer [16]. Regarding the latter, it has been reported that the expression of this enzyme is under estrogen receptor (ER) regulation and in ER‐positive breast cancers is associated with lower relapse rates as well as lower grade disease and better patient survival. It is also worth mentioning that this enzyme has received great attention as its expression is limited to a restricted number of normal tissues [17]. It has been recently discovered that this enzyme is co‐expressed and colocated with Pgp in drug‐resistant cancer cells. Although CA‐II is the most widely expressed isoform in normal tissues and is often absent from many types of cancer, it has been found that it can be highly overexpressed in gastrointestinal stromal tumors [18].

On the other hand, leishmaniasis is a well‐known major vector‐borne parasitic disease that affects nearly 88 tropical and subtropical developing countries. Notably, there are over twenty *Leishmania* species that can cause the three reported clinical forms of the disease in humans. Amidst them, *Leishmania major* (*L. major*) is responsible for cutaneous leishmaniasis (CL), the most common form, whereas *Leishmaniasis infantum* (*L. infantum*) causes visceral leishmaniasis (VL) [19]. Although there are some treatments available, such as pentavalent antimonials and other second‐line drugs (amphotericin B, miltefosine, paromomycin, and pentamidine), their use is usually hindered by the serious side effects ascribed to them (e.g., cardiac arrhythmias, nephrotoxicity, and pancreatitis). To make matters worse, resistance to these therapeutic options is rising to dangerously high levels. This underscores the imperative need to develop new, more effective, and less aggressive antileishmanial compounds [19, 20].

**Figure 1:**
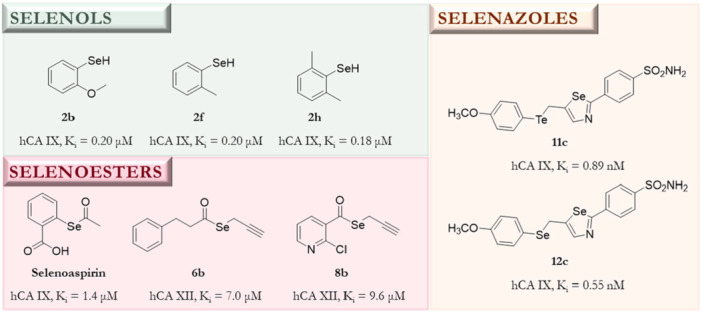
Chemical structures and **i**nhibition data of hCA IX and XII activity with several selenols (**2b**, **2f**, and **2h** [32]), selenoesters (**selenoaspirin**, **6b**, and **8b** [1, 33]), and selenazoles (**11c** and **12c** [5]).

A cohort of studies supports the notion that the incorporation of selenium (Se) moieties into a great variety of scaffolds renders bioactive compounds [21, 22, 23]. Thus, this strategy has been demonstrated to be a valid approximation for the development of potential drugs for the treatment of several diseases, such as leishmaniasis [24, 25], bacterial and viral infections [26, 27], and cancer [28, 29]. Additionally, some Se‐compounds have been reported to be CA inhibitors [1, 30]. Amidst them, selenoesters have stood out as they can release different active selenol fragments—a potent CA inhibitory chemotype—by the hydrolysis of the selenoester bond due to the selenolesterase activity of these metalloenzymes [31]. Figure 1 summarizes some of the reported selenocompounds with CA inhibitory activity.

In view of the former, we synthesized a small library of six selenoesters. Although the derivatives were initially designed for treating cancer as their structure resembled several clinical nonsteroidal antiandrogens (NSAAs), such as flutamide and bicalutamide, they were further tested as antileishmanial compounds. The rationale behind this is that it has been reported that numerous antitumoral compounds exert inhibitory activities against these parasites [34, 35, 36, 37]. Furthermore, several selenocompounds have been reported as promising candidates for the treatment of both diseases [21, 24, 25, 28, 38]. Regarding the antitumoral activities of the synthesized derivatives, all of them were submitted to the DTP of the NCI for an initial cytotoxic screening at 10 µM. Due to the promising results obtained, they were further selected for five‐dose evaluation. Their cytotoxic activities on nonmalignant HaCaT cells have also been reported as well as their capacity to inhibit several CA isoforms (hCA I, II, IX, and XII). Moreover, we have assessed their activity against two strains of *Leishmania*—*L. major* and *L. infantum*—along with their toxicity in healthy cells (THP‐1). The derivatives were found to be more effective than the standard drugs miltefosine and paromomycin in *L. major*, and two of them had no toxicity to the macrophages. Of note, the two trifluoromethoxy‐substituted anilide (TSA) derivatives (**3** and **6**) were the most effective ones in both strains, as they presented IC_50_ values below 10 µM.

## 2 Results and Discussion

### 2.1 Chemistry

We synthesized a new library of six selenoesters following a fragment‐based approach, varying the two terminal sides of the molecule. The design of the small library of compounds presented herein is based on some structural features present in NSAAs. Thus, the phenylcarboxamide scaffold is common to both first‐ and second‐generation NSAAs. Among all the functional groups synthetically available containing Se, that is, selenide, selenocyanate, diselenide, and trifluoromethylselenide, we decided to use the selenoester functionality based on several features: (i) the C‐Se bond in this functional group is more labile than in most of the other Se‐functional groups; (ii) an acyl group is presented in the most relevant NSAAs and the selenoester group is one of the few Se‐functional groups presenting an acyl group; (iii) the CAs have demonstrated to possess selenoesterase activity [31] which make them capable of cleave this functional group, hence maximizing the options to achieve CA inhibitors. On the one hand, the substitution of the *meta* and *para* positions of the aniline was encompassed with different substituents (H, CF_3_, OCF_3_, CN, and NO_2_). The trifluoromethyl group is present in the most relevant NSAAs, including flutamide, bicalutamide, and enzalutamide. Along with this functional group, the cyano and nitro groups are also decorating the phenyl ring in the NCAAs. We also decided to include a new substituent, the trifluoromethoxy group, given its similar electronic distribution when compared with CF_3_. On the other hand, in the selenoester moiety, we envisioned the introduction of a carbocyclic system (benzyl ring) and a heterocyclic system (two‐substituted furan ring). It has been reported that compounds with a furan nucleus present many biological applications. It is considered a potent pharmacological scaffold due to ease of synthesis and structural versatility of furan‐containing compounds [39, 40]. Furthermore, furan ring is present in several CA inhibitors [41].

**Scheme 1:**
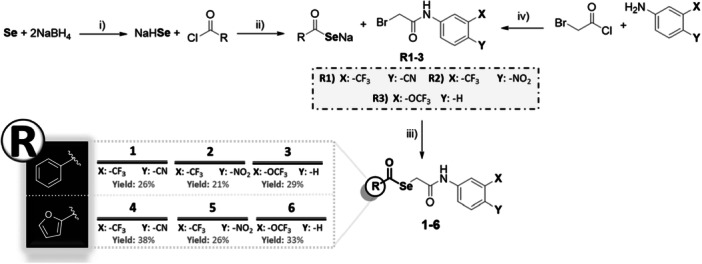
Synthesis of the target Se‐derivatives. Reagents and conditions: (i) H_2_O, 10–15 min, room temperature (R.T.); (ii) THF, R.T., 30–60 min; (iii) R.T. for 12 h; and (iv) DCM, K_2_CO_3_ or TEA, R.T.

The novel compounds were obtained following three reaction steps as a previously reported method for analogous derivatives [42], which is depicted in Scheme 1. First, sodium hydrogen selenide (NaHSe) was formed in situ by the reduction of elemental Se with NaBH_4_ in water. Then, benzoyl chloride or 2‐furoyl chloride was added to the reaction mixture and stirred for 30–60 min at room temperature (R.T.).

Finally, the bromide intermediates (R1–3) were added to the reaction, and the desired selenoesters (1–6) were obtained through a nucleophilic substitution. The synthetic procedure was carried out with yields ranging from 21% to 38%.

All the novel selenoesters were purified by column chromatography with a gradient of hexane and ethyl acetate as eluent and were stable at R.T. Their corresponding structures were elucidated by spectroscopic methods (^1^H, ^13^C, and ^77^Se nuclear magnetic resonance [NMR]) and elemental analysis as described in Section 4. The characterization of the six selenoderivatives is reported in Section 4, and the full spectra recorded are included in Supporting Information S1. An inspection of the ^1^H NMR spectra revealed that the most downfield shifted peak corresponded to proton linked to the NH of the anilide in the interval of 9–11 ppm. Regarding the ^13^C NMR spectra, a quartet signal that belonged to the –CF_3_ group appeared at 122–124 ppm (*J*_*C–F*_ = 272–274 Hz) as well as other quartets with smaller coupling constants. Likewise, in the case of compounds **3** and **4**, another quartet signal was ascribed to the –OCF_3_ group at ~121 ppm with *J*_*C–F*_ = 255.6–255.5 Hz, respectively. These coupling constants were in accordance with previous literature reports [43]. The signal corresponding to the carbonyl group of the selenoester bond appeared most shifted downfield, followed by the carbonyl group of the amide functionality. The peak belonging to the nitrile (–CN) group present in the aromatic ring was observed at 115.8 ppm, whereas the one corresponding to the –CH_2_– was found in the interval of 28–30 ppm. Overall, the chemical shifts of the Se peak appeared at 540–543 ppm.

### 2.2 Biology

#### 2.2.1 One‐Dose Assays of the NCI‐60 Human Tumor Cell Line Screening Program

**Figure 2:**
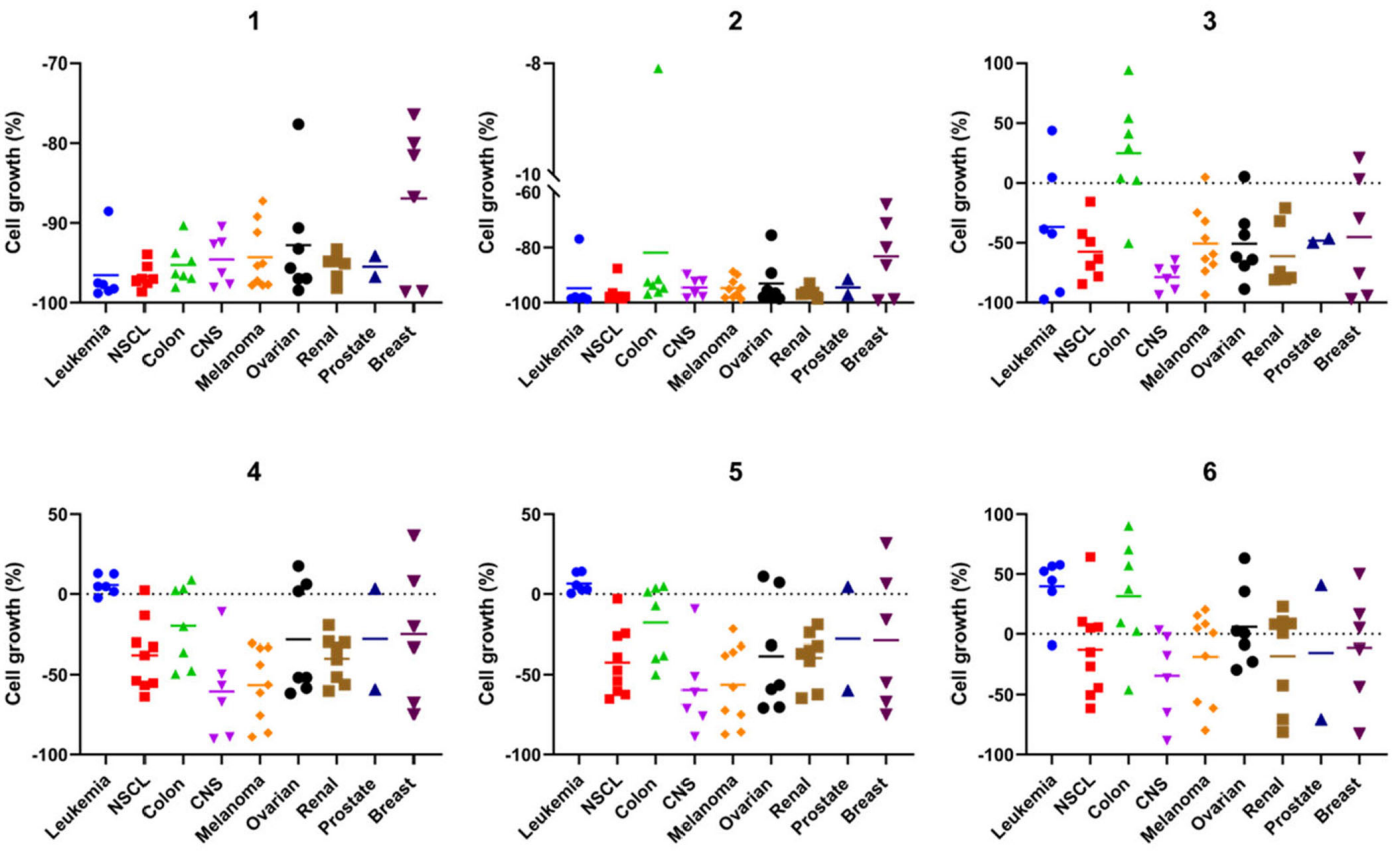
GP (%) of the NCI‐60 human cancer cell lines after treatment with a single dose of 10 µM of the reported selenoderivatives (**1–6**). CNS, central nervous system; NSCL, non‐small‐cell lung cancer.

**Figure 3:**
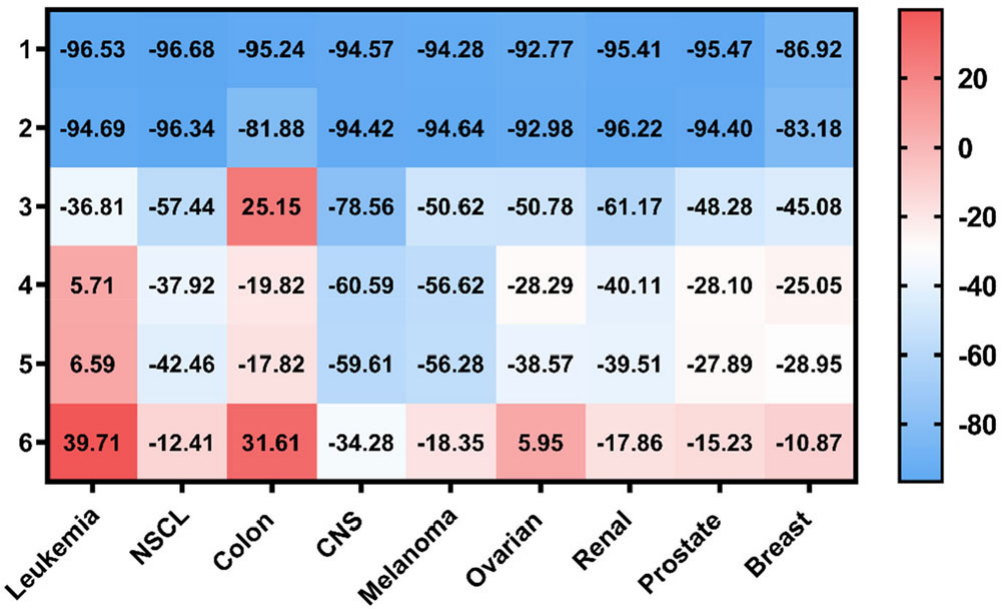
Summary of the average growth cell values (%) obtained in the NCI‐60 cytotoxicity screen at 10 µM for all the reported selenoderivatives (**1–6**).

To explore the potential antiproliferative properties of the newly synthesized derivatives, they were submitted to the National Cancer Institute's (NCI) Developmental Therapeutics Program (DTP) for screening at 10 μM in a panel of approximately 60 human cancer cell lines from different cancer types. The results are reported as the growth percentage (GP), which is the growth of the treated cells with each compound for 48 h compared to the untreated controls (Figure 2). GP between 0% and 50% means that the compound exhibits an antiproliferative activity, around 0 means cytostatic properties, and GP values between −100% and 0% stand for cytotoxic derivatives. The initial screening results are summarized in Figure 2, and the mean GP values of each subpanel are depicted in Figure 3. The full reports are compiled in the Supplementary Section.

The most active compounds were the benzene derivatives with mean GP values of −94.16% (**1**), −91.96% (**2**), and −43.99% (**3**) (Figures S19, S23, and S27). Regarding the data depicted in Figure 3, there were no significant differences between the average growth cell values obtained in each subpanel from the NCI in the case of **1** and **2**. Amidst the selenoesters bearing a phenyl ring, **3** was the only derivative that demonstrated potent cytotoxic activity in the HT29 colon cancer cell line. Interestingly, compound **3** exhibited pronounced cytotoxicity in the CNS subpanel, suggesting potential activity in brain cancer models. Conversely to the results obtained for **1**‐**3**, the furan derivatives (**4‐6**) did not exhibit cytotoxic behavior toward the tested cell lines from the leukemia subpanel, except for **6** against the HL‐60(TB) cells (Figures S31, S35, and S39). Besides, these three selenoesters were cytotoxic to the prostate DU‐145 cells, whereas they exhibited an antiproliferative effect against the PC‐3 cells. Additionally, numerous resistant cell lines from the NCI‐60 [44] were sensitive to the reported selenoesters. Of note, all the compounds exhibited cytotoxic behavior toward the most resistant cell lines from the CNS, breast, and prostate cancer subpanels (SNB‐19, DU‐145, and BT‐549, respectively). The HCC‐2998‐resistant colon cancer cells were susceptible to derivatives **1, 2**, and **5**. Besides, four compounds (**1, 2, 4**, and **5**) proved to have cytotoxic activity against OVCAR‐5, the most resistant cell line from the panel, whereas the TSA derivatives (**3** and **6**) exhibited a cytostatic behavior with GP values ranging from 2% to 5%.

#### 2.2.2 NCI Five‐Dose Assay

**Figure 4:**
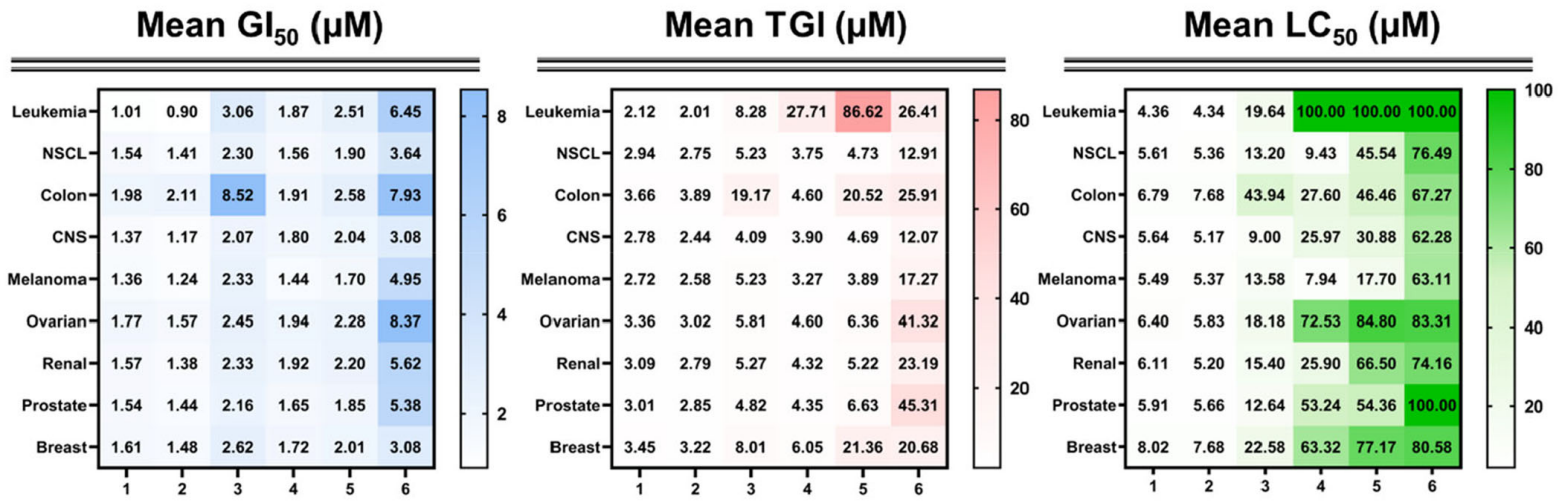
Average values of GI_50_, TGI, and LC_50_ values of all the reported selenoesters (**1–6**) against the NCI subpanels after the five‐dose assay.

All the compounds were selected for further testing since they fulfilled the set NCI one‐dose screening parameters for threshold inhibition. They were tested at five different concentrations (0.01, 0.1, 1, 10, and 100 μM) for 48 h against the same cell line panel and the dose–response curves along with three response parameters (50% growth inhibitory concentration [GI_50_], the 50% lethal concentration [LC_50_], and the total growth inhibition [TGI]) were calculated for every cancer cell line. The full reported data and corresponding graphs are compiled in Supporting Information S1. Average values of GI_50_, TGI, and LC_50_ values of all the reported selenoesters in all the subpanels are represented in Figure 4.

Overall, all the compounds exhibited potent anticancer activity with global mean GI_50_ values (1.53–5.39 µM) in the whole panel, which are greater values than the one obtained for 5‐fluorouracil (NSC 19893) (18 µM) in the same panel of cell lines [45]. Besides, all the compounds, except the two TSA derivatives (**3** and **6**), also exhibited mean GI_50_ values lower than gefitinib (NSC 715055) (3.2 µM) [45], and five of the synthesized compounds (**1–5**) were more effective than oxaliplatin (NSC 266046) (2.8 µM) [45]. Regarding the data depicted in Figure 4, there are no significant differences between the obtained mean GI_50_ values in each subpanel for **1** and **2** compared to their corresponding analogs (**4** and **5**). Although **6** was the least active compound, it exhibited significant inhibition potential with mean GI_50_ values below 10 µM in all the cancer subpanels tested. Another interesting point from the data is that several selenoesters demonstrated great antiproliferative activity against several resistant cell lines from the NCI‐60 [44]. Two benzene derivatives (**1** and **2**) displayed remarkable inhibitory activity with GI_50_ values of 0.64 and 0.28 µM, respectively, against the resistant breast cancer cell line HS 578 T (Figures S20 and S26). Remarkably, **2** also presented a TGI value of 0.89 µM, highlighting its potent antiproliferative effect in this cell line (Figure S26). Likewise, these compounds prompted the inhibition of cell growth in A498 renal‐resistant cells with GI_50_ values of 0.89 and 0.80 µM, respectively (Figures S20 and S26). It should also be pointed out that **2** demonstrated great growth inhibitory activity toward two more resistant cell lines, HOP‐62, and MALME‐3M, with GI_50_ values of 0.86 and 0.83 µM, respectively (Figure S26). According to the data summarized in Figure 4, 1 and 2 displayed an interesting antiproliferative activity against all the subpanels tested as they presented mean TGI values below 5 µM. Inspection of the LC_50_ results revealed two different behaviors between the evaluated compounds (Figure 4). Overall, the derivatives with the phenyl ring (**1–3**) exhibited the most potent cytotoxic activities in almost all the cancer subpanels. Conversely, the ones bearing a furyl ring (**4–6**) displayed LC_50_ values in the moderate‐high micromolar range in a wide array of cell lines from different types of cancer. Notably, the leukemia subpanel was the most resistant to the latter as they presented LC_50_ values higher than 100 µM in all the cell lines. Although **6** was devoid of activity in the prostate cancer subpanel, it can be observed that **4** and **5** present a unique selectivity in them (Figure 4). Thus, these selenoesters did not exhibit any cytotoxic effect toward the PC‐3 cells (LC_50_ > 100 µM), whereas they displayed outstanding activity in the resistant DU‐145 prostate cancer cell line (LC_50_ < 5 µM). Another important aspect perceived from Figures S38 and S44 is that **4** and **5** also had remarkable cytotoxicity against the OVCAR‐5 ovarian cancer cells, the most resistant cell line among the NCI‐60 panel. In the same line, the three furan derivatives (**4–6**) displayed certain selectivity in the breast cancer subpanel, as they did not exhibit cytotoxicity toward most of the cell lines.

#### 2.2.3 Carbonic Anhydrase

The inhibitory activity of all the selenoesters (**1–6**) was tested in vitro against the hCA isoforms I, II, IX, and XII, and their activities were compared to acetazolamide (AZZ), a standard CA inhibitor. As is evident from Table 1, among all the furan derivatives, **6** showed selectivity for cytosolic CA II isoform and tumor‐associated CA XII with K_I_ values in the low micromolar range (< 10 µM). Conversely, compounds **4** and **5** only inhibited tumor‐associated CA IX (*K_i_* = 19.0 and 21.1 µM, respectively). Another point that can be pointed out is that none of the benzene derivatives were able to inhibit any CA isoforms, except for compound **3**, the TSA derivative, which showed inhibition activity in the tumor‐associated CA XII isoform (*K_i_* = 17.4 µM). The selectivity shown by compounds **3**, **4**, and **5** is highly interesting as one of the major hurdles in the design and development of CA inhibitors is the lack of balanced isoform selectivity [46].

**Table 1:** Inhibition of hCA I, II, IX, and XII activity for compounds A–F.

K_I_ (µM)^a^				
Ref.	hCA I	hCA II	hCA IX	hCA XII
**1**	> 100	80.4	57.2	51.1
**2**	> 100	50.4	76.3	91.5
**3**	> 100	82.4	80.2	17.4
**4**	> 100	93.8	19.0	77.1
**5**	> 100	83.3	21.1	72.6
**6**	> 100	8.2	35.9	9.6
**AAZ**	0.25	0.012	0.026	0.006

Abbreviation: AZZ, acetazolamide.

^a^ Mean from three different assays, by a stopped flow technique (errors were in the range of ±5%–10% of the reported values).

#### 2.2.4 Cytotoxic Activity on Nonmalignant Cells

Encouraged by the impressive antitumoral activity of compounds **1**–**3**, we decided to evaluate their cytotoxic activity in nonmalignant HaCaT cells using the [3‐(4,5‐dimethylthiazol‐2‐yl)‐5‐(3‐carboxymethoxyphenyl)‐2‐(4‐sulfophenyl)‐2*H*‐tetrazolium] (MTS) assay as previously described [47]. Thus, the selenoesters were tested at seven different concentrations (1, 2.5, 5, 10, 25, 50, and 100 µM) during 48 h to determine their corresponding IC_50_ values. According to data depicted in Table 2, it can be observed that the TSA compound (**3**) displayed the lowest cytotoxicity among the tested selenoesters (IC_50_ ≈ 64 µM).

**Table 2:** Antiproliferative activity of the hit compounds expressed as average IC_50_ values.

IC_50_ (µM)^a^	
Ref.	HaCaT
**1**	11.79 ± 1.89
**2**	9.49 ± 0.42
**3**	63.71 ± 4.32

^a^ IC_50_ values are presented as the mean ± SD of at least three independent experiments determined by the MTS assay.

**Figure 5:**
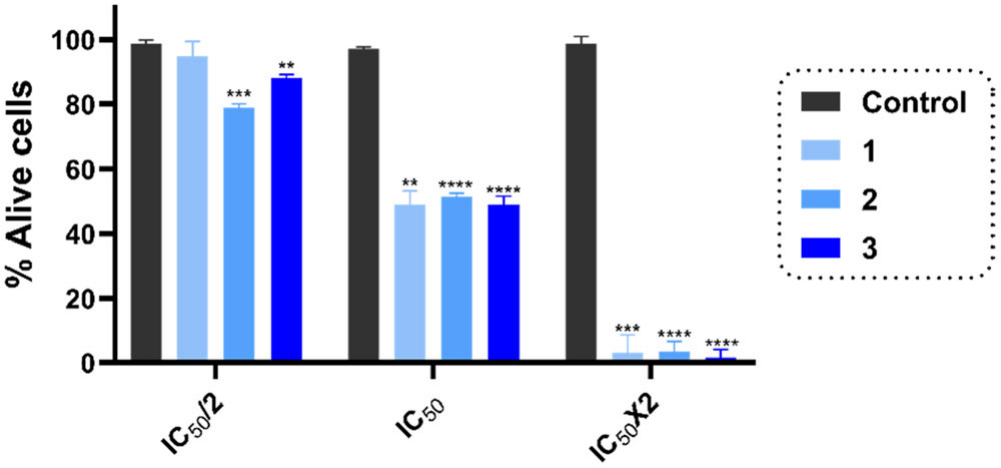
Effect of the selected selenoesters (**1**, **2**, and **3**) on the inhibition of cell viability. Data are expressed as the mean ± SD of three independent experiments. ****p < 0.0001, ***p < 0.001, and **p < 0.01 when comparing compounds with the control.

To further support the antiproliferative results obtained in the MTS assay, we performed the trypan blue dye exclusion assay in HaCaT cells (Figure 5) as previously described [47]. Cells were treated for 48 h with three concentrations of the compounds (half the IC_50_ [IC_50_/2], IC_50_, and two times the IC_50_ [2 × IC_50_]). The population of dead and alive cells was calculated, and the results are expressed as the percentage of alive cells. From the data, it seems clear that the inhibition of cell viability was dose‐dependent and was in concordance with our previous results.

#### 2.2.5 Antileishmanial Activity and Cytotoxicity in THP‐1 Cells

The synthesized compounds were tested for their antileishmanial activity against promastigotes of *L. major* and *L. infantum* at four concentrations (1, 10, 50, and 100 µM) for 48 h using the 3‐(4,5‐dimethylthiazol‐2‐yl)‐2,5‐diphenyltetrazolium bromide (MTT) assay. In this evaluation, the activity was expressed in IC_50_ (concentration inhibiting the parasite growth by 50%). The derivatives with IC_50_ values below 25 µM were selected as hit derivatives and evaluated against THP‐1 mammalian cells at five different concentrations to determine their cytotoxic concentration (CC_50_) values. The CC50 was defined as the concentration of selenoesters that led to a reduction in the viability of the macrophages, such that only 50% of the cells survived when compared to the control group. This cell line, derived from the blood of a human with acute monocytic leukemia, was chosen as it has been widely reported that it resembles tissue macrophages to various extents as well as supports the infection by numerous *Leishmania* promastigotes. The selectivity indexes (SIs) were calculated as the ratio between toxicity on macrophages (THP‐1 cells)/antileishmanial activity. The data for standard control drugs (miltefosine and paromomycin) are included for comparison purposes.

As reported in Table 3, among the tested compounds, it was determined that the TSA derivatives (**3** and **6**) were the most effective ones with IC_50_ values in the low micromolar range (6–10 µM) in both *L. major* and *L. infantum* promastigotes. Besides, compound **3** exhibited a CC_50_ value greater than 200 µM. Likewise, **5** stood out with moderate activity against *L. major* (IC_50_ = 18.27 µM) and without significant toxicity toward the THP‐1 macrophages (CC_50_ = 108.01 µM). It is also remarkable that all the compounds demonstrated better activity in *L. major* promastigotes than the reference drugs (miltefosine and paromomycin). Furthermore, five derivatives (**1–4** and **6**) were more effective than miltefosine in *L. infantum*, whereas only the TSA compounds (**3** and **6**) presented a better antileishmanial activity toward these parasites than paromomycin. It should also be noted that **3**, **5**, and **6** exhibited higher SI than the reference drugs.

**Table 3:** IC_50_ values of all the novel synthesized compounds against *L. major* and *L. infantum* promastigotes, cytotoxic activity against THP‐1 cells, and their corresponding SI.

Ref.	*L. major*	*L. infantum*	THP‐1	SI^c^	
	IC_50_ (µM)^a^	IC_50_ (µM)	CC_50_ (µM)^b^	*L. major*	*L. infantum*
**1**	26.59 ± 1.81	29.16 ± 2.65	—	—	—
**2**	27.07 ± 3.35	29.36 ± 2.10	—	—	—
**3**	9.67 ± 0.78	6.59 ± 3.73	> 200	> 20.68	> 30.35
**4**	26.47 ± 3.69	28.55 ± 2.40	—	—	—
**5**	18.27 ± 5.90	32.87 ± 8.25	108.01 ± 5.53	5.91	3.29
**6**	10.11 ± 1.27	5.99 ± 2.33	38.71 ± 6.71	3.83	6.47
**Miltefosine** d	66.6 ± 11.6	31.9 ± 2.3	13.3 ± 2.8	0.2	0.4
**Paromomycin** d	71.0 ± 3.3	18.0 ± 1.0	39.0 ± 13.7	0.6	2.2

^a^ IC_50_ is defined as the concentration that causes a 50% inhibition of the promastigote proliferation.

^b^ CC_50_ is defined as the concentration required to reduce cell viability by 50%.

^c^ SI is the ratio of the CC_50_ against THP‐1 cells and the IC_50_ in the *L. major* and *L. infantum* promastigotes. The results are expressed as mean ± SD of at least three independent experiments in triplicate.

^d^ References [24, 38].

### 2.3 ADMET Predictions

**Figure 6:**
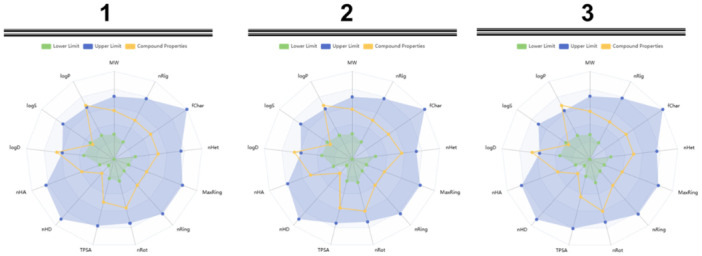
Radar view of the physicochemical properties of **1–3**. fChar, formal charge (Optimal: −4 to 4, based on Drug‐Like Soft rule); logD, logarithm of the n‐octanol/water distribution coefficients at pH = 7.4 (Compounds in the range from 1 to 3 log mol/L are considered proper); logP, logarithm of the n‐octanol/water distribution coefficient (Compounds in the range from 0 to 3 log mol/L are considered proper); logS, logarithm of aqueous solubility value (compounds in the range from −4 to 0.5 log mol/L are considered proper); MaxRing, number of atoms involved in the biggest system ring (Optima: 0–18, based on Drug‐Like Soft rule); MW, molecular weight (Optimal: 100–600, based on Drug‐Like Soft rule); nHA, number of hydrogen bond acceptors (Optimal: 0–12, based on Drug‐Like Soft rule); nHD, number of hydrogen bond donors (Optimal: 0–7, based on Drug‐Like Soft rule); nHet, number of heteroatoms (Optimal: 1–15, based on Drug‐Like Soft rule); nRing, number of rings (Optimal: 0–6, based on Drug‐Like Soft rule); nRot, number of rotatable bonds (Optimal: 0–11, based on Drug‐Like Soft rule); TPSA, topological polar surface area (Optimal: 0–140, based on Veber rule).

The theoretical absorption, distribution, metabolism, excretion, and toxicity (ADMET) profiles of **1–3** were predicted using ADMETlab 3.0 algorithm [48] and Swiss ADME server [49]. None of the studied compounds violated any of Lipinski's 5 rules and are not considered pan‐assay interference compounds (PAINS). Although the CaCo‐2 cell permeability findings showed that only 3 had an optimal membrane permeability, all of them possessed a high likelihood of being effectively absorbed through the intestinal membrane according to the obtained values for human intestinal absorption (HIA). In addition, the AMES toxicity profile showed that none of them presented the potential to be toxic. The full reports are shown in Figures S55–S57 and Table S1. The radar view of the physicochemical properties of **1–3** is depicted in Figure 6.

## 3 Conclusions

In summary, we have synthesized and characterized a small library of novel selenoesters. All the compounds were submitted to the DTP of the NCI and exhibited a promising antitumoral profile. Compounds **1**–**3** were further evaluated against the human keratinocyte HaCaT cell line. Moreover, bearing in mind that CA has been demonstrated to display a selenoesterase activity and that numerous selenocompounds have been reported to effectively inhibit several hCA isoforms, we further tested all our compounds against hCA I, II, IX, and XII. Compound **6** stood out as it inhibited tumor‐associated isoform XII and the cytosolic hCA II isoform in the low micromolar range (*K_i_* values < 10 μM). Notably, **3**, the other TSA, showed selectivity for hCA‐XII. On the other hand, all the compounds were evaluated in vitro against both *L. major* and *L. infantum* promastigotes as well as THP‐1 mammalian cells to determine their antileishmanial activities and SI. Compound **3** stood out as it was the most effective one against both promastigotes (IC_50_ values < 10 μM) and displayed no cytotoxicity against the macrophages derived from THP‐1 (CC_50_ ≥ 200 μM). Although more experiments are required to study in depth the antileishmanial, antitumoral, and hCA inhibitory properties of the reported compounds, the results herein reported could encourage the development of novel phenylcarboxamide‐selenium derivatives.

Furthermore, this study possesses great novelty in that although several selenoester derivatives with potent antitumor activity can be found in the literature, this is the first report where phenylcarboxamide‐Se derivatives present potent dual antitumor and leishmanicidal activity. Only three articles [25, 50, 51] have reported the antileishmanial effect of some selenoesters; all of them showed lesser potency and selectivity when compared with compound **3**. Notwithstanding, none of the previously reported selenoesters with leishmanicidal activity have demonstrated any relevant antitumor activity. Thereby, the results herein reported could pave the way to further development of selenoester derivatives with potent activity toward both cancer and leishmania. Although the cytotoxicity and selectivity results, along with the ADMET predictions, are very early stage, compound **3** could represent a good candidate or lead compound to develop a future clinical candidate to treat leishmaniasis and/or cancer.

## 4 Experimental

### 4.1 Chemistry

#### 4.1.1 General

All the chemicals were purchased from commercial suppliers and used as received. Reaction courses were monitored by thin‐layer chromatography (TLC) plates, and the spots were visualized using an ultraviolet (UV) lamp. The ^1^H, ^13^C, and ^77^Se NMR were recorded using a Bruker Avance Neo 400 MHz operating at 400, 100, and 76 MHz, respectively, using deuterated DMSO (DMSO‐*d*_6_) or deuterated acetone (acetone‐*d*_6_) as solvents and tetramethylsilane (TMS) as an internal signal. Chemical shift (δ) values are in parts per million (ppm), and the coupling constants (J) values are reported in hertz (Hz). Standard abbreviations indicating multiplicity are used as follows: bs = broad singlet, s = singlet, d = doublet, dd = double doublet, and t = triplet. Melting points (mp) were determined on a Metler FP82 + FP80 apparatus. Elemental analyses for H, N, and C were carried out on a Thermo Fisher FlashSmart Elemental Analyzer. The purity of the synthesized compounds was assessed by analytical high‐performance liquid chromatography (HPLC) using Supelco (C18 10 µM, 25 cm × 4.6 nm) column and DAD1B detector at a wavelength of 254 nm with a linear gradient of 40%–100% methanol/water in 30 min at a flow rate of 1 mL/min.

#### 4.1.2 General Procedure for the Synthesis of the Intermediates (R1–R3)

Bromoacetyl chloride (5.4 mmol) was added to a mixture of the corresponding anilines in dichloromethane (DCM) containing triethylamine (TEA) (for intermediate **R1**, 5.4 mmol) or an excess of K_2_CO_3_ (for **R2** and **R3**) or for 24 h. In the first case, the reaction mixtures were extracted with DCM (3 × 20 mL) and washed with distilled water (2 × 30 mL) to collect the organic material. The organic layers were dried with anhydrous sodium sulfate and concentrated in vacuo. For intermediates **R2** and **R3**, the resulting precipitate (KBr) was filtered off. The filtrate was concentrated under vacuum by rotatory evaporation to afford the crude product. The crude products were used without further purification.

#### 4.1.3 General Procedure for the Synthesis of the Novel Selenoesters (1–6)

A solution of sodium borohydride (9.4 mmol) in distilled water (12.5 mL) was added to a stirred suspension of gray Se (4.7 mmol) in distilled water (12.5 mL) at R.T. until an almost colorless solution of NaHSe was formed (approximately after 10 min). Then, the corresponding acid chlorides (4.7 mmol) were added, and the reaction mixture was stirred for 30‐60 min at R.T. Then, the corresponding halide intermediates (R1–R3, 4.7 mmol) were added to the reaction, and the mixture was stirred overnight at R.T. These intermediates were prepared as previously described and used without further purification. The crude compounds were isolated by extraction with DCM (3 × 20 mL) and washed with distilled water (2 × 30 mL) to collect the organic phases. Afterward, they were dried with anhydrous sodium sulfate. The solvent was removed under vacuum by rotatory evaporation. The derivatives were purified by silica gel column chromatography with a gradient of hexane and ethyl acetate as eluent.

*Se‐(2‐((4‐cyano‐3‐(trifluoromethyl)phenyl)amino)‐2‐oxoethyl)benzoselenoate (1)*. The title compound was synthesized from R1 and obtained as a yellow solid according to the general procedure described above. Yield: 25.94%, MP: 138.7°C. ^1^H NMR (400 MHz, DMSO‐*d*_6_) *δ* (ppm): 11.13 (bs, 1H, NH), 8.26 (d, *J*_H‐H_ = 2.1 Hz, 1H, H_Aryl_), 8.10 (d, *J*_H‐H_ = 8.5 Hz, 1H, H_Aryl_), 7.98 (dd, *J*_H‐H_ = 8.6 and 2.1 Hz, 1H, H_Aryl_), 7.91 (d, *J*_H‐H_ = 7.9 Hz, 2H, H_Benz_), 7.75 (t, *J*_H‐H_ = 7.6 Hz, 1H, H_Benz_), 7.59 (dd, *J*_H‐H_ = 8.0 and 7.8 Hz, 2H, H_Benz_), 4.04 (s, 2H, –CH_2_–). ^13^C NMR (101 MHz, DMSO‐*d*_6_) *δ* (ppm): 193.1 (–Se‐C=O), 168.5 (–NH‐C=O), 143.5, 137.5, 136.6, 134.7, 131.8 (q, *J*_C‐F_ = 31.8 Hz), 129.5, 127.0, 122.42 (q, *J*_C‐F_ = 273.7 Hz, –CF_3_), 122.0, 116.4 (q, *J*_C‐F_ = 5.1 Hz), 115.8 (–CN), 101.9 (q, *J*_C‐F_ = 2.3 Hz), 29.1 (*–*CH_2_–). ^77^Se NMR (76 MHz, DMSO‐*d*_6_) *δ* (ppm): 542. Anal. Calcd for C_17_H_11_F_3_N_2_O_2_Se (%): C, 49.65; H, 2.70; N, 6.81. Found: C, 49.84; H, 2.99; and N, 6.93. HPLC: *R*_T_ = 35.0 min; purity = 97.7%.

*Se‐(2‐((4‐nitro‐3‐(trifluoromethyl)phenyl)amino)‐2‐oxoethyl)benzoselenoate* (*2*). The title compound was synthesized from R2 and obtained as an orange solid according to the general procedure described above. Yield: 20.94%, MP: 142.6°C. ^1^H NMR (400 MHz, Acetone‐*d*_6_) *δ* (ppm): 10.16 (bs, 1H, NH), 8.34 (s, 1H, H_Aryl_), 8.13 (s, 2H, H_Aryl_), 7.94 (d, *J*_H‐H_ = 7.8 Hz, 2H, H_Benz_), 7.73 (t, *J*_H‐H_ = 7.4 Hz, 1H, H_Benz_), 7.58 (dd, *J*_H‐H_ = 7.9 and 7.7 Hz, 2H, H_Benz_), and 4.07 (s, 2H, –CH_2_–). ^13^C NMR (101 MHz, Acetone‐*d*_6_) *δ* (ppm): 193.8 (–Se‐C=O), 169.3 (–NH‐C=O), 144.3, 143.4, 138.9, 135.4, 130.2, 128.2, 124.8 (q, *J*_C‐F_ = 33.7 Hz), 123.2, 123.1, 123.0 (q, *J*_C‐F_ = 272.6 Hz, –CF_3_), 118.5 (q, *J*_C‐F_ = 6 Hz), and 29.9 (–CH_2_–, signal under solvent). ^77^Se NMR (76 MHz, Acetone‐*d*_6_) *δ* (ppm): 543. Anal. Calcd for C_16_H_11_F_3_N_2_O_4_Se (%): C, 44.56; H, 2.57; and N, 6.50. found: C, 44.47; H, 2.31; and N, 6.43.

*Se‐(2‐oxo‐2‐((3‐(trifluoromethoxy)phenyl)amino)ethyl)benzoselenoate (3)*. The title compound was synthesized from R3 and obtained as a yellow gel according to the general procedure described above. Yield: 28.87%.^1^H NMR (400 MHz, Acetone‐*d*_6_) *δ* (ppm): 9.68 (bs, 1H, NH), 7.95 (d, *J*_H‐H_ = 7.4 Hz, 2H, H_Benz_), 7.87 (s, 1H, H_Aryl_), 7.73 (t, *J*_H‐H_ = 7.4 Hz, 1H, H_Benz_), 7.59 (dd, *J*_H‐H_ = 7.8 and 7.6 Hz, 2H, H_Benz_), 7.55 (d, *J*_H‐H_ = 8.3 Hz, 1H, H_Aryl_), 7.43 (dd, *J*_H‐H_ = 8.3 and 8.2 Hz, 1H, H_Aryl_), 7.03 (d, *J*_H‐H_ = 8.2 Hz, 1H, H_Aryl_), and 4.00 (s, 2H, –CH_2_–). ^13^C NMR (101 MHz, Acetone‐*d*_6_) δ (ppm): 194.1 (–Se‐C=O), 168.5 (–NH‐C=O), 150.3, 141.8, 139.2, 135.4, 131.3, 130.3, 128.1, 121.6 (q, *J*_C‐F_ = 255.6 Hz, –OCF_3_), 118.7, 116.6, 112.7, and 30.0 (–CH_2_–, signal under solvent). ^77^Se NMR (76 MHz, Acetone‐*d*_6_) *δ* (ppm): 543. Anal. Calcd for C_16_H_12_F_3_NO_3_Se (%): C, 47.78; H, 3.01; and N, 3.48. Found: C, 47.96; H, 3.31; and N, 3.56. HPLC: *R*_T_ = 36.8 min; purity = 96.9%.

*Se‐(2‐((4‐cyano‐3‐(trifluoromethyl)phenyl)amino)‐2‐oxoethyl)furan‐2‐carboselenoate (4)*. The title compound was synthesized from R1 and obtained as a yellow solid according to the general procedure described above. Yield: 37.98%, MP: 133.5°C. ^1^H NMR (400 MHz, DMSO‐*d*_6_) *δ* (ppm): 11.10 (bs, 1H, NH), 8.24 (d, *J*_H‐H_ = 2.1 Hz, 1H, H_Aryl_), 8.12 (d, *J*_H‐H_ = 1.7 Hz, 1H, H_Furoyl_), 8.10 (d, *J*_H‐H_ = 8.6 Hz, 1H, H_Aryl_), 7.96 (dd, *J*_H‐H_ = 8.5 and 2.1 Hz, 1H, H_Aryl_), 7.49 (d, *J*_H‐H_ = 3.7 Hz, 1H, H_Furoyl_), 6.82 (dd, *J*_H‐H_ = 3.7 and 1.7 Hz, 1H, H_Furoyl_), and 4.00 (s, 2H, –CH_2_–).^13^C NMR (101 MHz, DMSO‐*d*_6_) *δ* (ppm): 179.9 (–Se‐C=O), 168.5 (–NH‐C=O), 150.8, 148.9, 143.5, 136.6, 131.8 (q, *J*_C‐F_ = 31.9 Hz), 122.4 (q, *J*_C‐F_ = 273.7 Hz, –CF_3_), 122.0, 116.7, 116.4 (q, *J*_C‐F_ = 5.2 Hz), 115.8 (–CN), 113.6, 101.8 (q, *J*_C‐F_ = 2.4 Hz), and 28.1 (–CH_2_–). ^77^Se NMR (76 MHz, DMSO‐*d*_6_) *δ* (ppm): 540. Anal. Calcd for C_15_H_9_F_3_N_2_O_3_Se (%): C, 44.90; H, 2.26; and N, 6.98. Found: C, 45.23; H, 2.45; and N, 7.09. HPLC: *R*_T_ = 31.1 min; purity = 95.5%.

*Se‐(2‐((4‐nitro‐3‐(trifluoromethyl)phenyl)amino)‐2‐oxoethyl)furan‐2‐carboselenoate (5)*. The title compound was synthesized from R2 and obtained as a yellow solid according to the general procedure described above. Yield: 25.98%, MP: 137.6°C. ^1^H NMR (400 MHz, DMSO‐*d*_6_) *δ* (ppm): 11.15 (bs, 1H, NH), 8.25 (d, *J*_H‐H_ = 2.2 Hz, 1H, H_Aryl_), 8.19 (d, *J*_H‐H_ = 9.0 Hz, 1H, H_Aryl_), 8.12 (d, *J*_H‐H_ = 1.7 Hz, 1H, H_Furoyl_), 8.02 (dd, *J*_H‐H_ = 9.0 and 2.3 Hz, 1H, H_Aryl_), 7.48 (d, *J*_H‐H_ = 3.7 Hz, 1H, H_Furoyl_), 6.81 (dd, *J*_H‐H_ = 3.8 and 1.8 Hz, 1H, H_Furoyl_), and 4.00 (s, 2H, –CH_2_–). ^13^C NMR (101 MHz, DMSO‐*d*_6_) *δ* (ppm): 179.9 (–Se‐C=O), 168.4 (–NH‐C=O), 150.8, 148.9, 143.5, 141.5, 127.8, 123.0 (q, *J*_C‐F_ = 33.2 Hz), 122.3, 122.0 (q, *J*_C‐F_ = 273.0 Hz, –CF_3_), 117.2 (q, *J*_C‐F_ = 6 Hz), 116.6, 113.6, and 28.1 (–CH_2_–). ^77^Se NMR (76 MHz, DMSO‐*d*_6_) *δ* (ppm): 540. Anal. Calcd for C_14_H_9_F_3_N_2_O_5_Se (%):C, 39.92; H, 2.15; and N, 6.65. Found: C, 40.06; H, 2.36; and N, 6.69. HPLC: *R*_T_ = 32.5 min; purity = 99.1%.

*Se‐(2‐oxo‐2‐((3‐(trifluoromethoxy)phenyl)amino)ethyl)furan‐2‐carboselenoate (6)*. The title compound was synthesized from R3 and obtained as a yellow gel according to the general procedure described above. Yield: 33.37%. ^1^H NMR (400 MHz, Acetone‐*d*_6_) *δ* (ppm): 9.67 (bs, 1H, NH), 7.94 (d, *J*_H‐H_ = 1.7 Hz, 1H, H_Furoyl_), 7.85 (s, 1H, H_Aryl_), 7.53 (d, *J*_H‐H_ = 8.2 Hz, 1H, H_Aryl_), 7.42 (dd, *J*_H‐H_ = 8.2 and 8.1 Hz, 1H, H_Aryl_), 7.36 (d, *J*_H‐H_ = 3.6 Hz, 1H, H_Furoyl_), 7.03 (d, *J*_H‐H_ = 8.1 Hz, 1H, H_Aryl_), 6.76 (dd, *J*_H‐H_ = 3.7 and 1.7 Hz, 1H, H_Furoyl_), 3.96 (s, 2H, –CH_2_–). ^13^C NMR (101 MHz, Acetone‐*d*_6_) *δ* (ppm): 181.1 (–Se‐C=O), 168.4 (–NH‐C=O), 152.5, 150.2, 148.9, 141.7, 131.1, 121.4 (q, *J*_C‐F_ = 255.5 Hz, –OCF_3_), 118.6, 116.5, 114.1, 112.6, and 28.4 (–CH_2_–). ^77^Se NMR (76 MHz, Acetone‐*d*_6_) *δ* (ppm): 543. Anal. Calcd for C_14_H_10_F_3_NO_4_Se (%): C, 42.87; H, 2.57; and N, 3.57. Found: C, 42.65; H, 2.33; and N, 3.50. HPLC: *R*_T_ = 33.0 min; purity = 97.0%.

### 4.2 Biological Assays

#### 4.2.1 NCI‐60 Analysis

All the reported selenoesters (**1–6**) were submitted to the DTP of NCI. First, they were screened at a single dose (10^−5^ M) against a panel of 60 cancer cell lines derived from different tumor types following the methodology available (https://dtp.cancer.gov/discovery_development/nci-60/methodology.htm). As all the compounds satisfied the threshold inhibition criteria established, they were further assessed at five concentrations (0.01, 0.1, 1, 10, and 100 µM) against the same cell panel to obtain their corresponding dose–response curves and three antitumor parameters (GI_50_, TGI, and LC_50_) by regression. GraphPad Prism 7.0 Software was used to represent the corresponding graphs.

#### 4.2.2 Cell Culture Conditions

The cell lines were obtained from the American Type Culture Collection (ATCC). Human monocytic leukemia cells, THP‐1, were cultured in RMPI‐1640 medium (Gibco, USA) supplemented with 10% heat‐inactivated FBS (Gibco, USA), 1 mM HEPES, 2 mM glutamine, 1 mM sodium pyruvate, and 5% penicillin/streptomycin, pH 7.2 at 37°C and 5% CO_2_. Human keratinocyte HaCaT cells were cultured in DMEM (ThermoFisher Scientific, USA), supplemented with 10% FBS (Gibco, USA) and 1% antibiotics (10.00 units/mL penicillin and 10.00 mg/mL streptomycin; Gibco, USA). The cells were incubated in tissue culture flasks at 37°C and 5% CO_2_.

#### 4.2.3 THP‐1 Cytotoxicity

THP‐1 cells were seeded at a concentration of 8 × 10^4^ cells per well in 96‐well plates and incubated with phorbol 12‐myristate 13‐acetate (PMA) (50 ng/mL) for 72 h (37°C and 5% CO_2_) to differentiate them into macrophages. After that, the culture medium was removed and the cells were treated with the reported selenoesters at different increasing concentrations ranging from 1 to 200 µM for 48 h at 37°C and 5% of CO_2_. Macrophages treated with DMSO (1%) were used as controls, and the compounds were dissolved in DMSO at a stock concentration of 10^−2^ M. Afterward, the MTT assay was performed. Data were obtained from three independent experiments performed in triplicate. The absorbance was measured at 550 nm using a MultiskanEX photometric plate reader for microplates. The CC_50_ values were calculated using OriginPro 8.5.1. software by nonlinear curve fitting. SIs were calculated as the ratio of the CC_50_ values determined for the macrophages and the IC_50_ values obtained in each *Leishmania* promastigote. GraphPad Prism 7.0 Software was used to obtain the corresponding graphs.

#### 4.2.4 Cell Viability Assay

The effect of each compound on the cell viability of HaCaT cells was tested using the MTS assay [47]. The compounds (**1–6**) were dissolved in DMSO at a concentration of 10^−2^ M, and serial dilutions were prepared. Briefly, the cells were seeded in a 96‐well plate at a density of 1 × 10^4^ cells per well. After 24 h of incubation, the cells were treated with either DMSO or seven different concentrations of each selenoester (1, 2.5, 5, 10, 25, 50, and 100 µM) for 48 h in triplicate. At the end of the treatment duration, the media were removed and fresh media were added containing 20 μL of MTS (Promega, USA) per well. After 1 h incubation, the absorbances were read at a wavelength of 490 nm in a 96‐well multiscanner, and the ratio of viable cells was calculated. Data were obtained from at least three independent experiments performed in triplicate. The IC_50_ values were calculated using OriginPro 8.5.1 Software for nonlinear curve fitting, and Graphpad Prism 7.0 Software was used to represent the corresponding graphs.

#### 4.2.5 Trypan Blue Staining Assay

Briefly, HaCaT cells were seeded in 12‐well plates at a density of 5 × 10^5^ cells per well for 24 h. Then, cells were treated with three serial concentrations (2 × IC_50_, IC_50_, and IC_50_/2) of **1**, **2**, and **3** for 48 h. The cells were harvested after the end of the treatments and mixed with 1:4 volume of 0.4% Trypan Blue dye (Invitrogen, MA, USA). Cells were loaded over the hemocytometer and counted separately using a bright‐field microscope. Viable cells presented a clear, bright appearance, while dead or membrane‐compromised cells were stained with the dye. Data were obtained from three independent experiments performed in triplicate. GraphPad Prism 7.0 Software was used to obtain the corresponding graphs.

#### 4.2.6 Carbonic Anhydrase Inhibition Studies

An Applied Photophysics stopped‐flow instrument was used to assay the CA‐catalyzed CO_2_ hydration activity [52]. Phenol red (at a concentration of 0.2 mM) was used as an indicator, working at the absorbance maximum of 557 nm, with 20 mM Hepes (pH 7.4) as a buffer, and 20 mM Na_2_SO_4_ (to maintain constant ionic strength), following the initial rates of the CA‐catalyzed CO_2_ hydration reaction for a period of 10–100 s. The CO_2_ concentrations ranged from 1.7 to 17 mM for the determination of the kinetic parameters and inhibition constants [53]. Enzyme concentrations ranged between 5 and 12 nM. For each inhibitor, at least six traces of the initial 5%–10% of the reaction were used to determine the initial velocity. The uncatalyzed rates were determined in the same manner and subtracted from the total observed rates. Stock solutions of the inhibitor (0.1 mM) were prepared in distilled–deionized water, and dilutions up to 0.01 nM were done thereafter with the assay buffer. Inhibitor and enzyme solutions were preincubated together for 15 min at room temperature before the assay, to allow for the formation of the E–I complex. The inhibition constants were obtained by Nonlinear least‐squares methods using PRISM 3 and the Cheng‐Prusoff equation as reported earlier and represent the mean from at least three different determinations. All CA isoforms were recombinant proteins obtained in‐house, as reported earlier [54, 55, 56].

#### 4.2.7 Parasite Culture Conditions

The promastigotes of *L. major* (clone VI, MHOM/IL/80/Friendlin) and *L. infantum* (BCN‐150) were grown at 26°C under continuous shaking in M199 1× medium (Sigma, USA). This media was supplemented with 10% of heat‐inactivated fetal bovine serum (FBS) (Gibco, USA), 0.0005% (w/v) hemin, 0.1 mM adenine, 25 mM HEPES (pH 7.2), 0.0001% (w/v) biotin, and 100 UI/mL penicillin and 100 mg/mL streptomycin (Gibco, USA).

#### 4.2.8 Leishmanicidal Activity Against Promastigotes

The antileishmanial activity of the reported selenoesters was determined using the MTT assay as previously described during the logarithmic growth phase of the tested promastigotes of *L. major* and *L. infantum*. Briefly, the *Leishmania* parasites were seeded into 96‐well flat‐bottom plates (3 × 10^5^ parasites/100 µL) and treated with four different concentrations ranging between 1 and 100 µM of each selenoester at 26°C for 48 h. Afterwards, 20 µL of MTT (5 mg/mL in PBS) was added to each well and incubated for 4 h under the same conditions. Then, 80 µL/well of DMSO was added to dissolve the resultant formazan crystals. The absorbances were determined on a Multiskan EX photometric plate reader for microplates at 540 nm. Three independent experiments performed in triplicate were carried out for each compound, and OriginPro 8.5.1 Software was used to calculate the IC_50_ values by fitting the data to a dose–inhibition sigmoid curve.

### 4.3 Statistical Analysis

Data were expressed as the mean ± standard deviation (SD) and experiments were performed at least three times in triplicate unless otherwise noted. Nonlinear curve regression analysis calculated by OriginPro version 8.5.1 Software was used to obtain the IC_50_ and CC_50_ values obtained for the synthesized selenoderivatives. The unpaired *t*‐test was used to calculate the statistical significance of differences comparing the selenoesters and the control. Data were analyzed using GraphPad Prism version 8.0.1, and the statistically significant values (*p* value) for unpaired *t*‐test analysis were taken as ****p < 0.0001, ***p < 0.001, and **p < 0.01 when comparing control and compounds.

## Conflicts of Interest

The authors declare no conflicts of interest.

## Supporting information

Supp_R1.

## Data Availability

The data that support the findings of this study are available on request from the corresponding author.
