# Supplementary material for: New Se‐Compounds With Antileishmanial, Antitumor, and Carbonic Anhydrase Inhibitory Properties
Source: Arch Pharm (Weinheim). 2025 Dec 3;358(12):e70145. doi: 10.1002/ardp.70145 (PMC12676205; doi:10.1002/ardp.70145)
Supplement: Supplementary file 1 — Supp_R1. [file ARDP-358-e70145-s001.pdf]

New Se-compounds with antileishmanial, antitumor, and carbonic anhydrase inhibitory properties

Cristina Morán-Serradilla [1], Daniel Plano [1], Andrea Angelli [2], Arun K. Sharma [3], Carmen Sanmartin [1,*], and Claudiu T. Supuran [2]

1 Department of Pharmaceutical Sciences, University of Navarra, Irunlarrea 1, E-31008 Pamplona, Spain
2 Department NEUROFARBA – Pharmaceutical and nutraceutical section, University of Firenze, Sesto Fiorentino, Florence, Italy
3 Department of Molecular and Precision Medicine, Penn State College of Medicine, 500 University Drive, Hershey, PA 17033, USA.

*Correspondence:
Dr. Carmen Sanmartin, Department of Pharmaceutical Sciences, University of Navarra, Irunlarrea 1, E-31008 Pamplona, Spain
Email: sanmartin@unav.es

Table of contents:

**Chemical characterization**	
^{1}H, ^{13}C and ^{77}Se NMR spectra	
1	Figures S1-3
2	Figures S4-6
3	Figures S7-9
4	Figures S10-12
5	Figures S13-15
6	Figures S16-18
Analytical HPLC chromatograms	
1	Figure S19
3	Figure S20
4	Figure S21
5	Figure S22
6	Figure S23
**Biological evaluation**	
NCI-60 results	
1	Figures S24-29
2	Figures S30-35
3	Figures S36-41
4	Figures S42-47
5	Figures S48-53
6	Figures S54-59
***In silico* studies**	
ADMET	Table S1<br>Figures S60-62

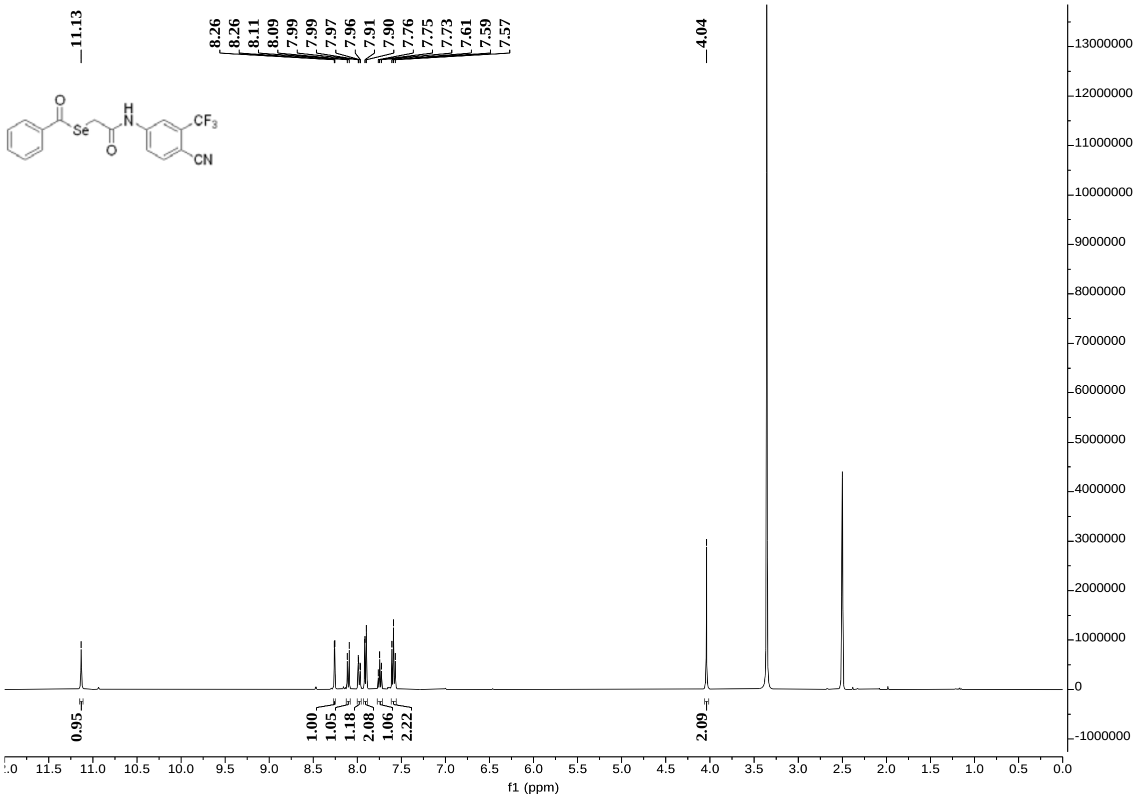

Figure S1. ^{1}H-NMR spectrum of compound **1.**

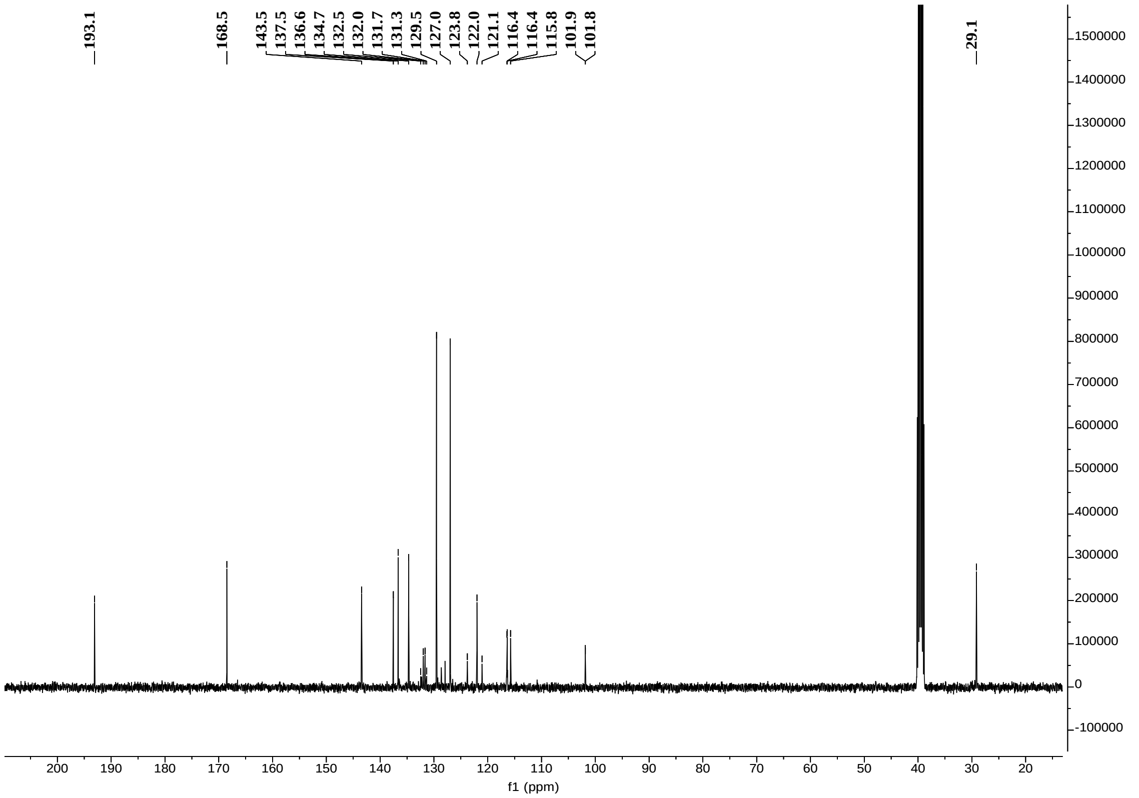

Figure S2. ^{13}C-NMR spectrum of compound **1**.

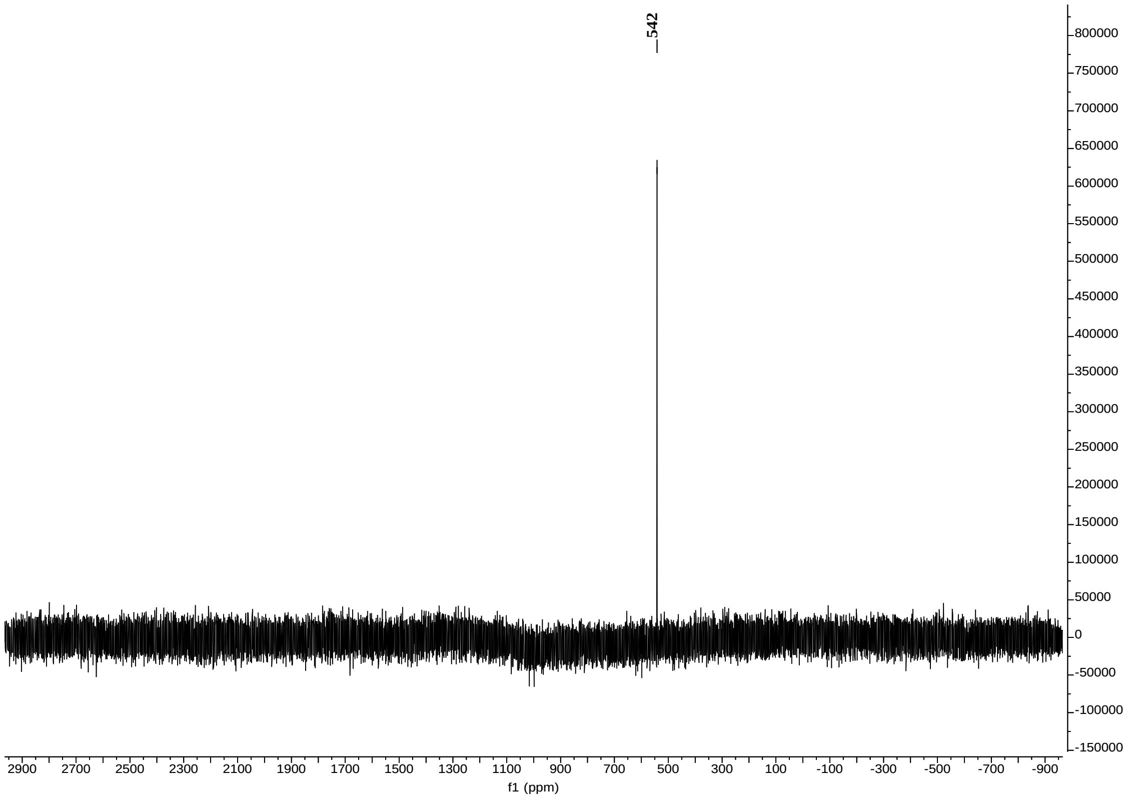

Figure S3. ^{77}Se-NMR spectrum of compound **1**.

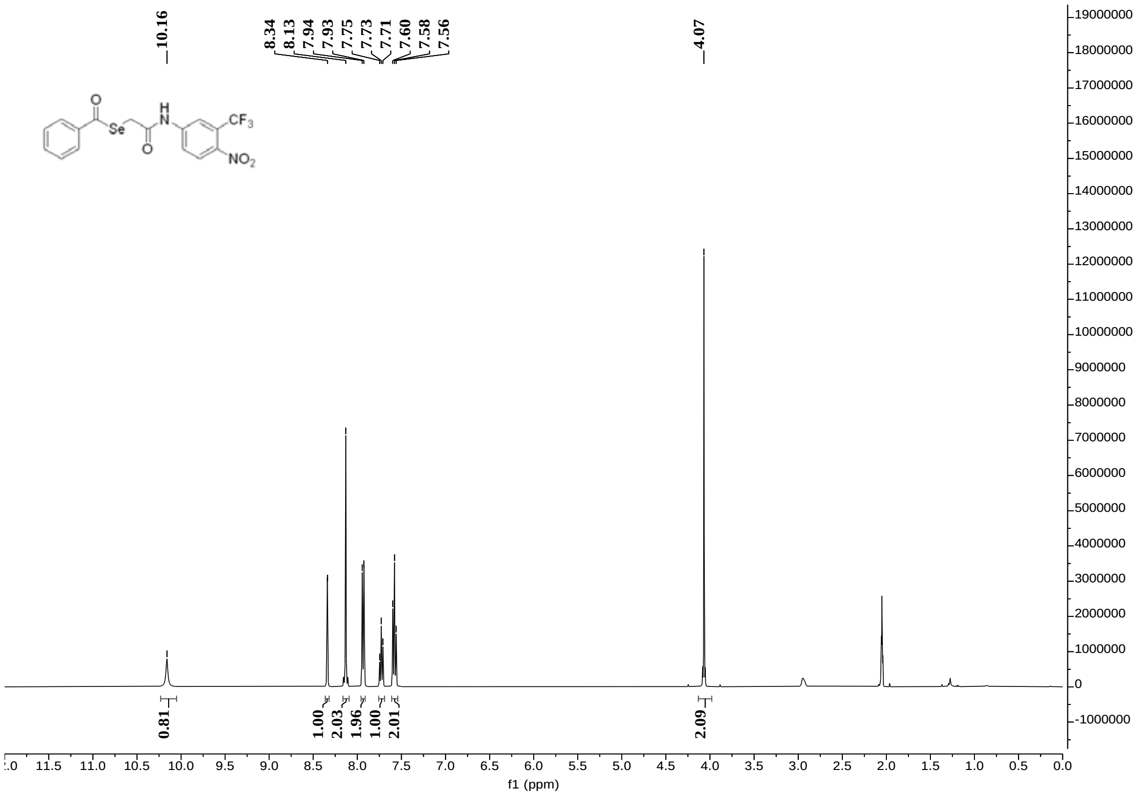

Figure S4. ^{1}H-NMR spectrum of compound **2.**

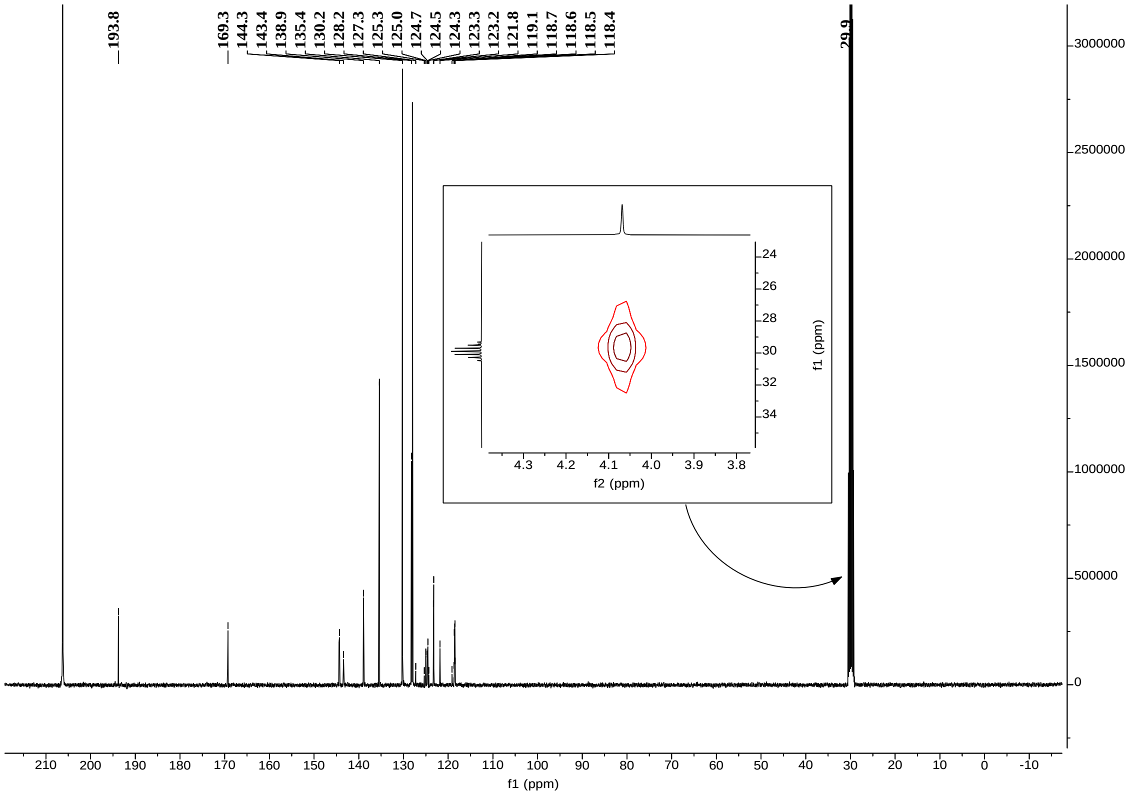

Figure S5. ^{13}C-NMR spectrum of compound **2**. A zoomed in region of the HMQC spectrum is shown.

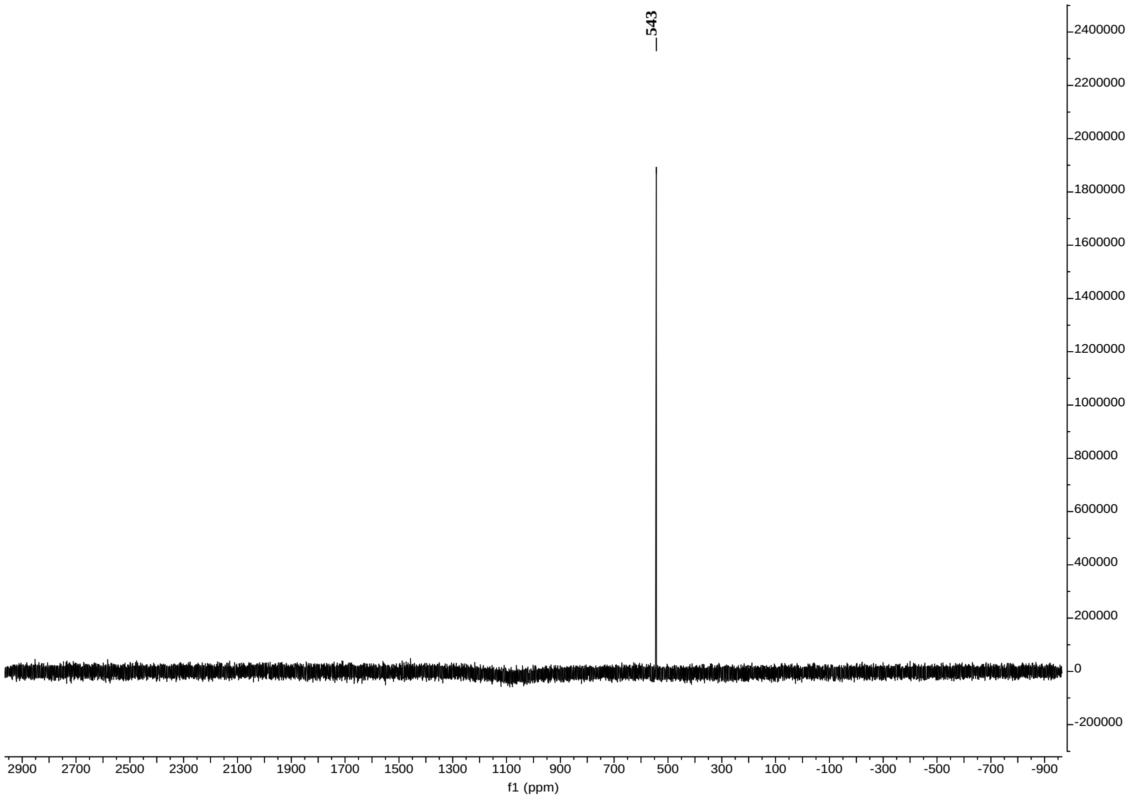

Figure S6. ^{77}Se-NMR spectrum of compound **2**.

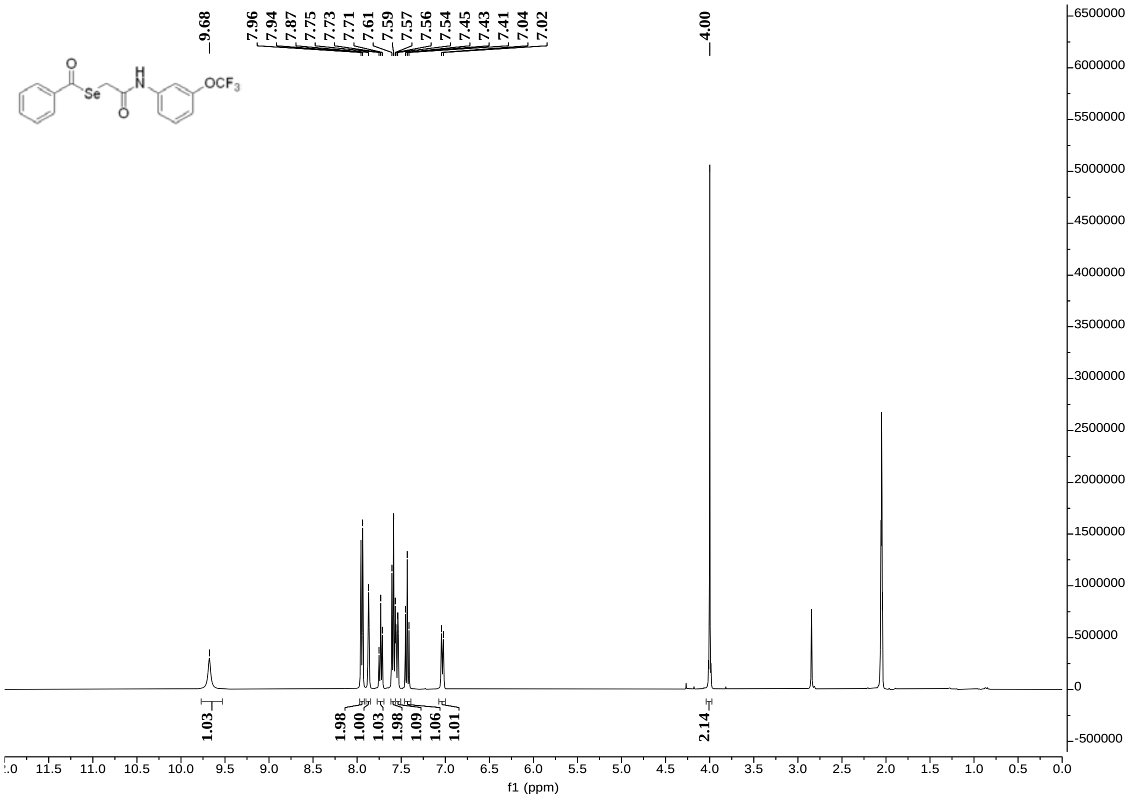

Figure S7. ^{1}H-NMR spectrum of compound **3.**

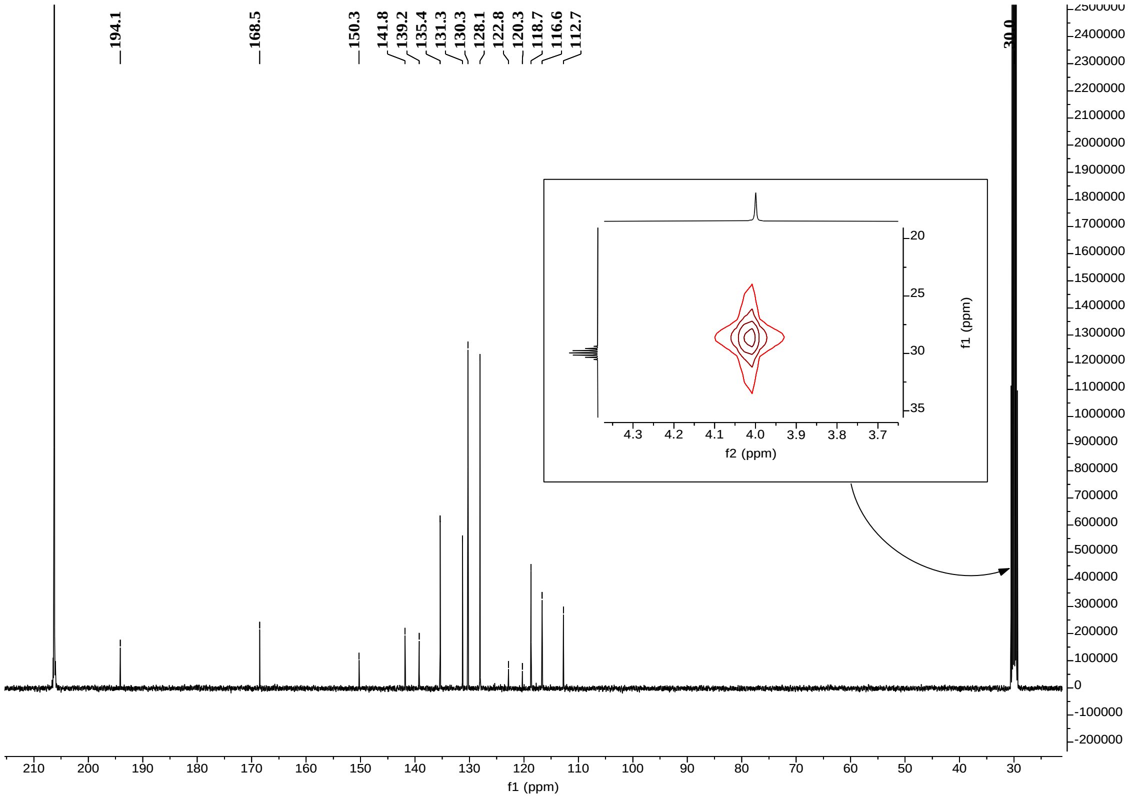

Figure S8. ^{13}C-NMR spectrum of compound **3**. A zoomed in region of the HMQC spectrum is shown.

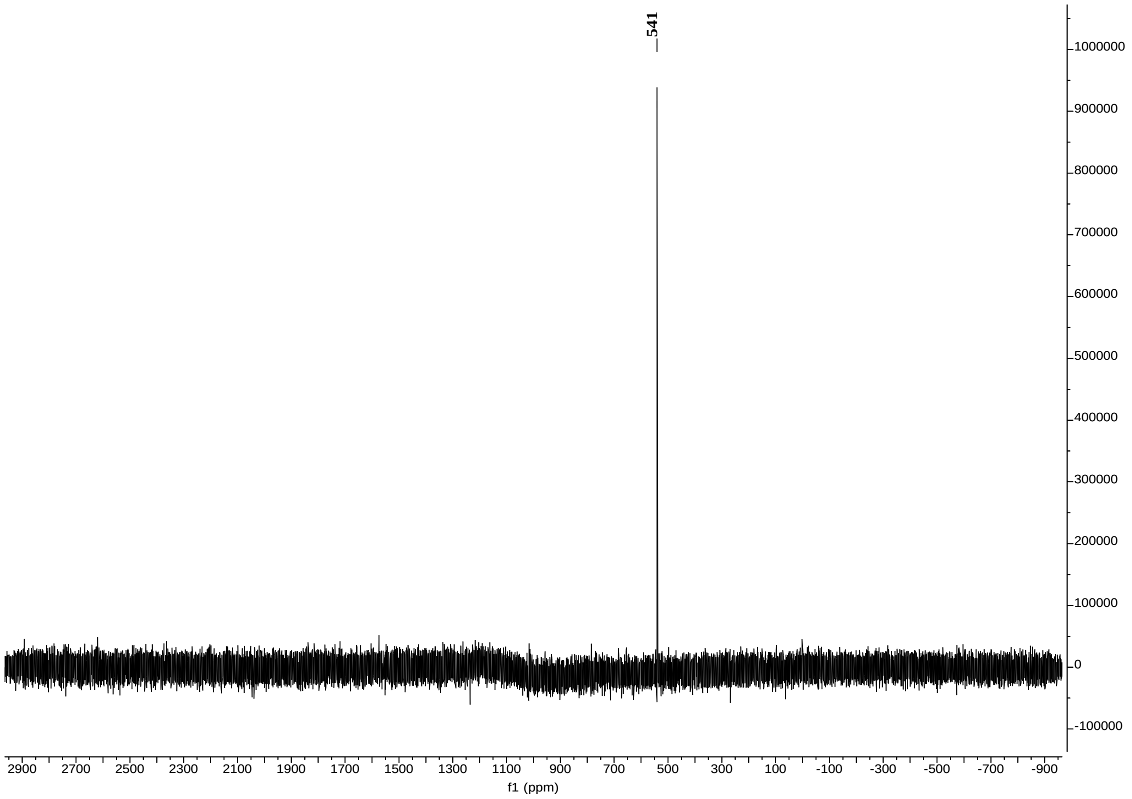

Figure S9. ^{77}Se-NMR spectrum of compound **3**.

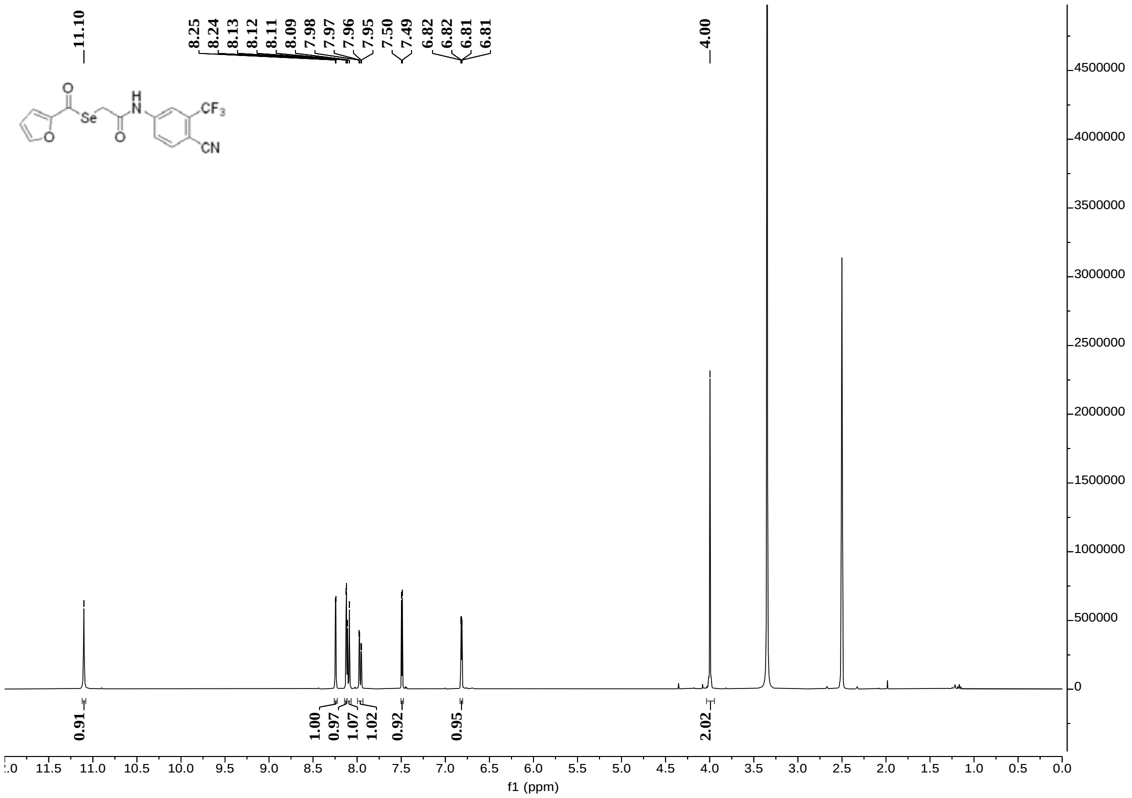

Figure S10. ^{1}H-NMR spectrum of compound **4.**

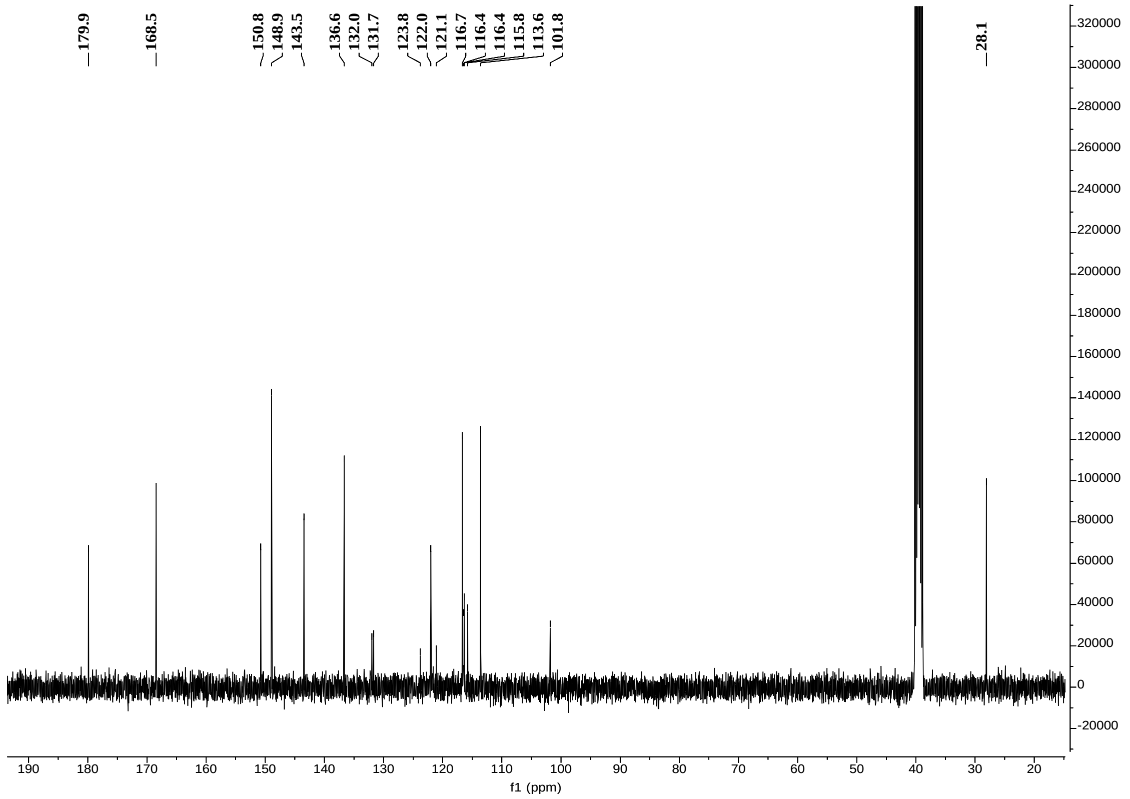

Figure S11. ^{13}C-NMR spectrum of compound **4**.

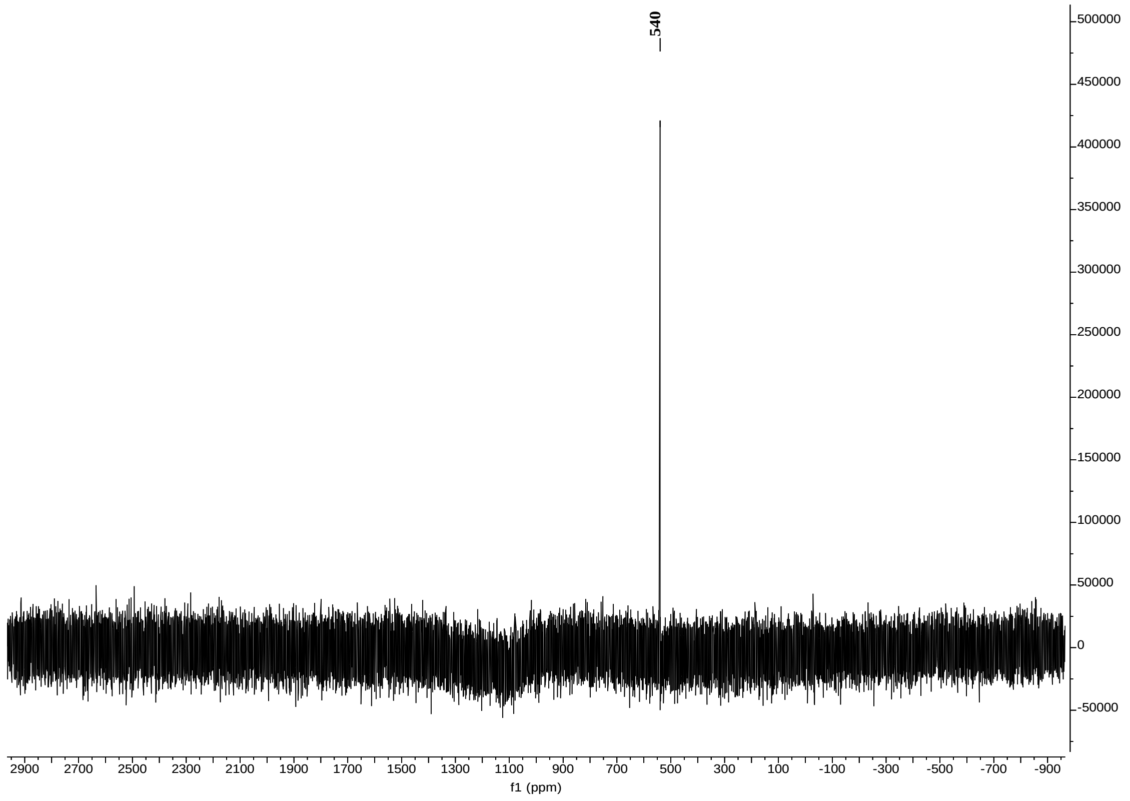

Figure S12. ^{77}Se-NMR spectrum of compound **4**.

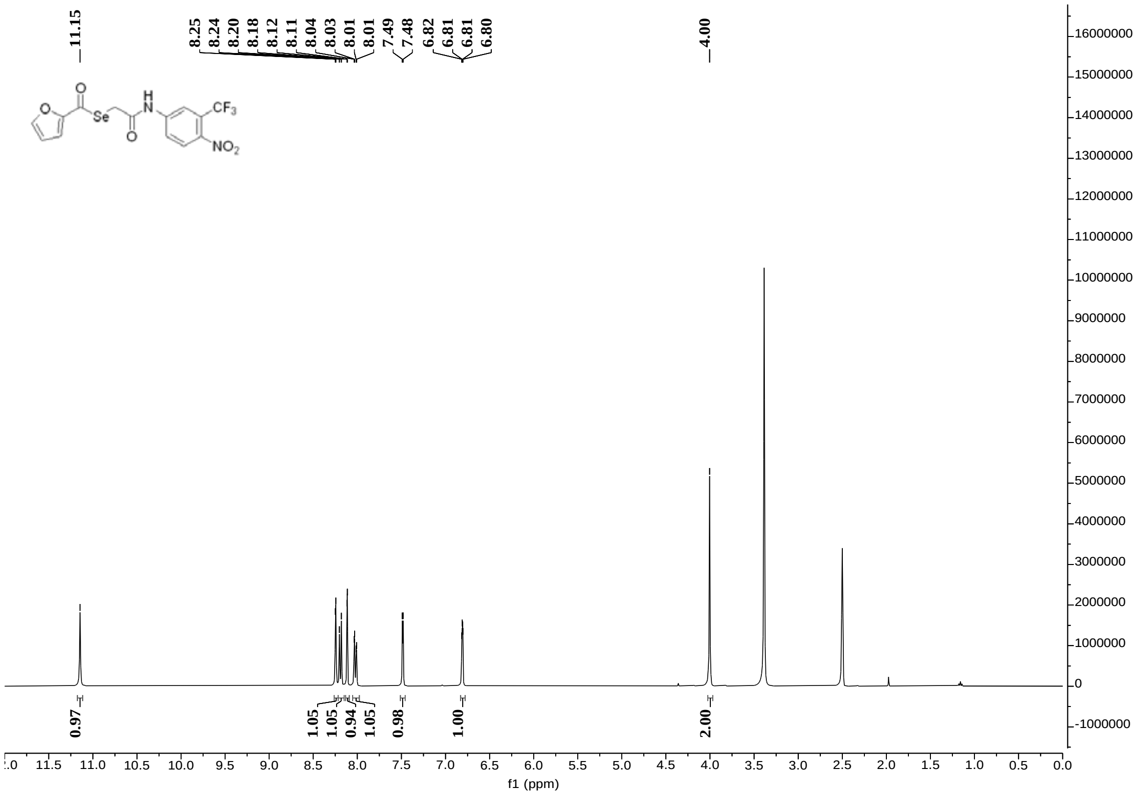

Figure S13. ^{1}H-NMR spectrum of compound **5.**

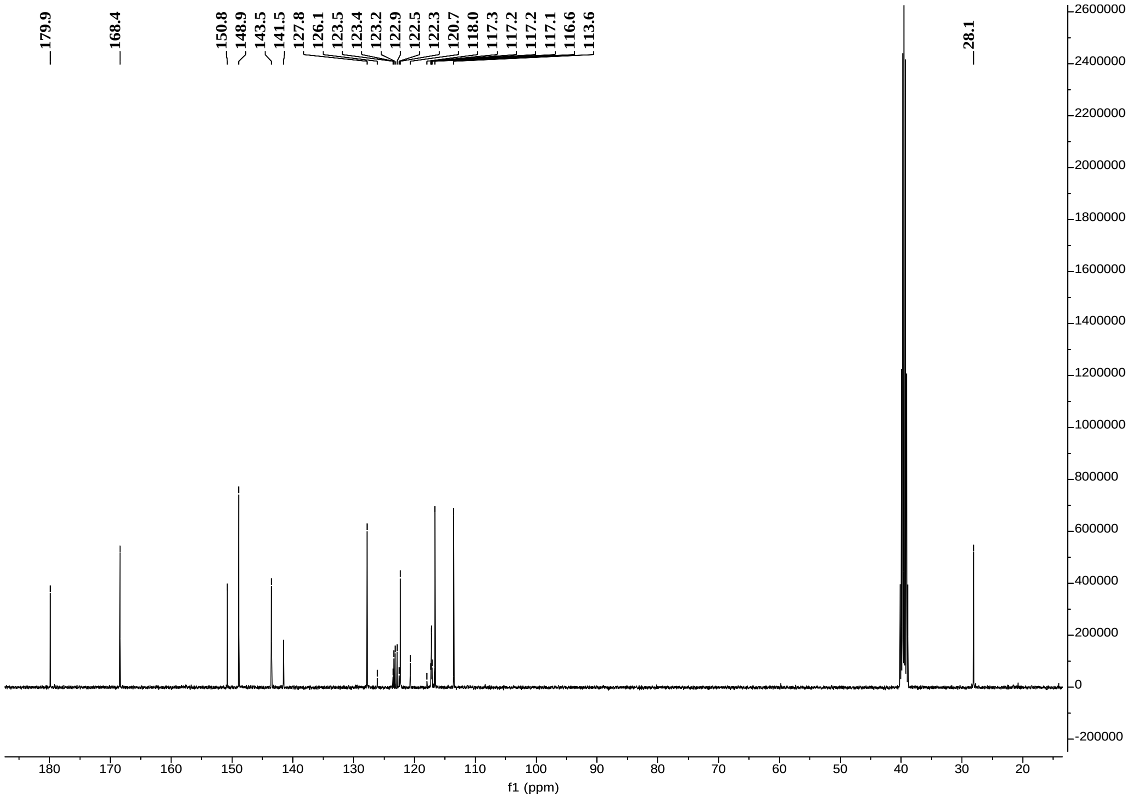

Figure S14. ^{13}C-NMR spectrum of compound **5**.

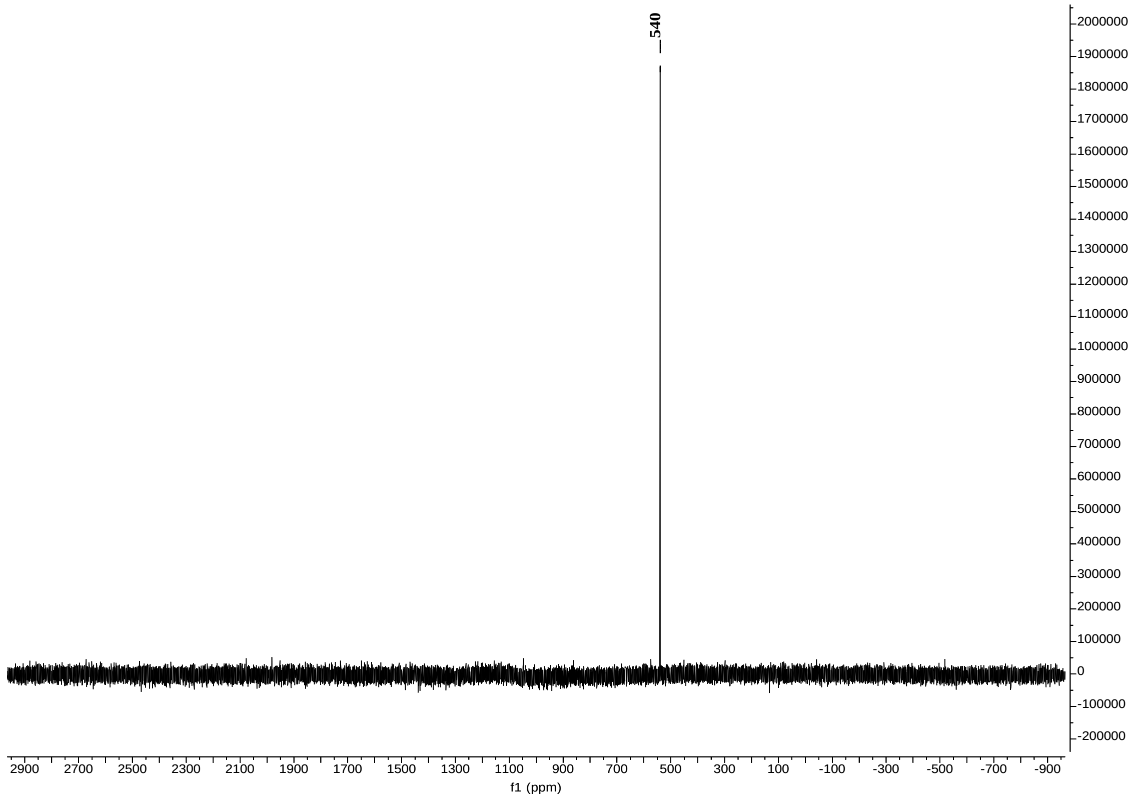

Figure S15. ^{77}Se-NMR spectrum of compound **5**.

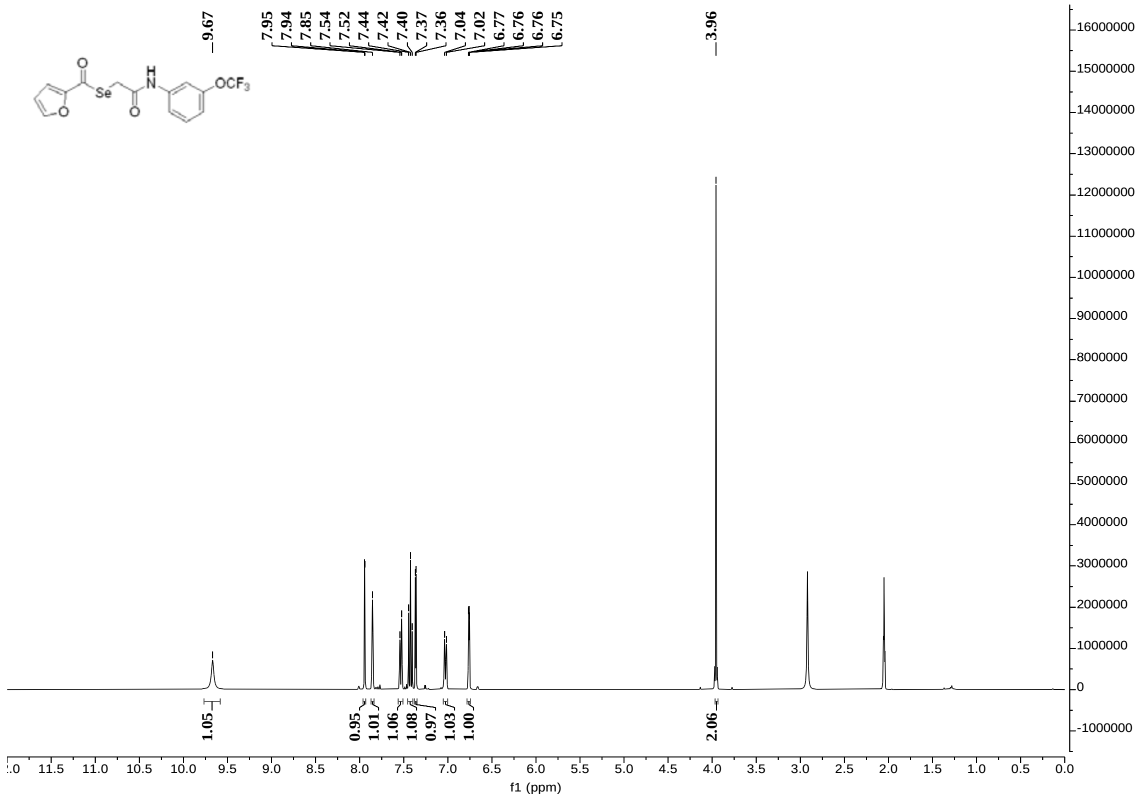

Figure S16. ^{1}H-NMR spectrum of compound **6.**

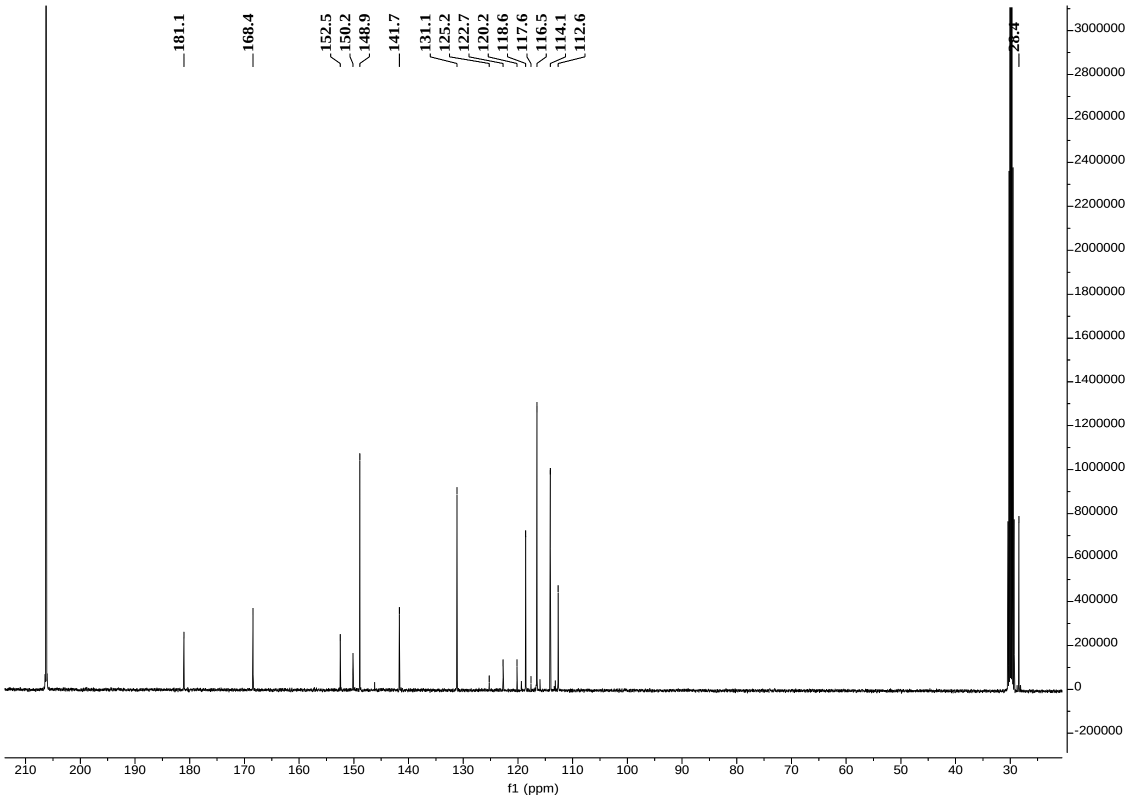

Figure S17. ^{13}C-NMR spectrum of compound **6**.

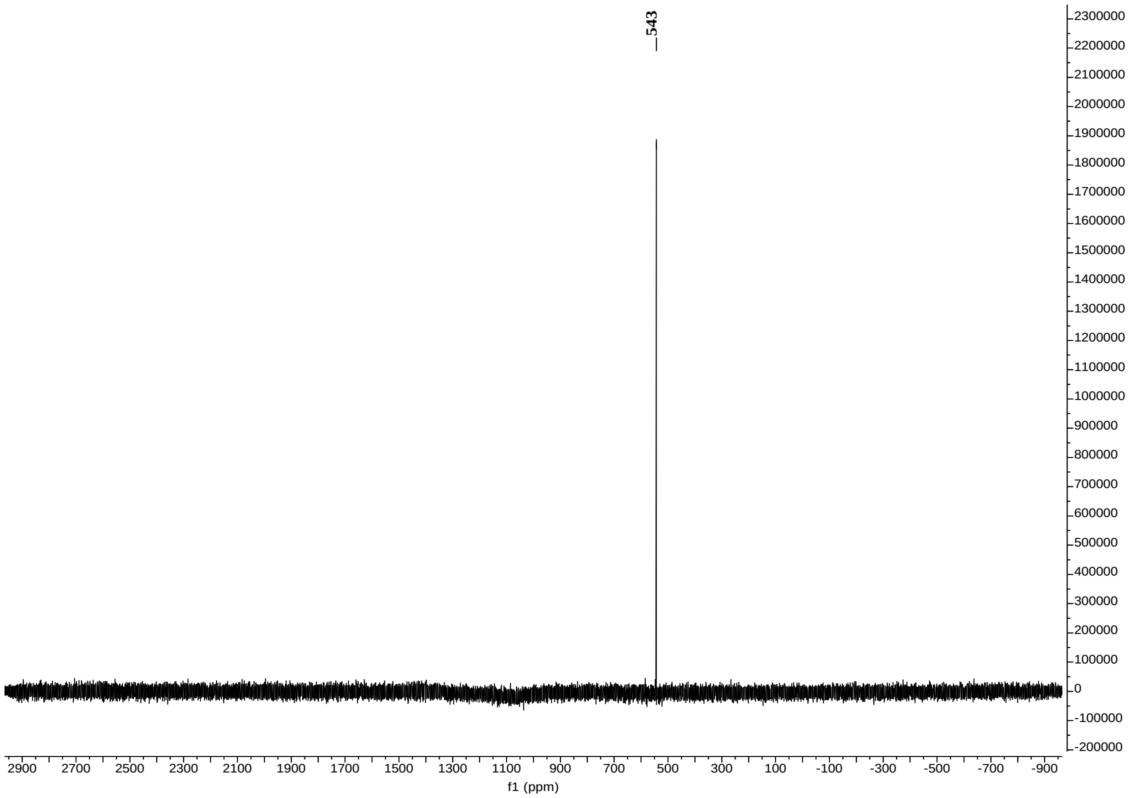

Figure S18. ^{77}Se-NMR spectrum of compound **6**.

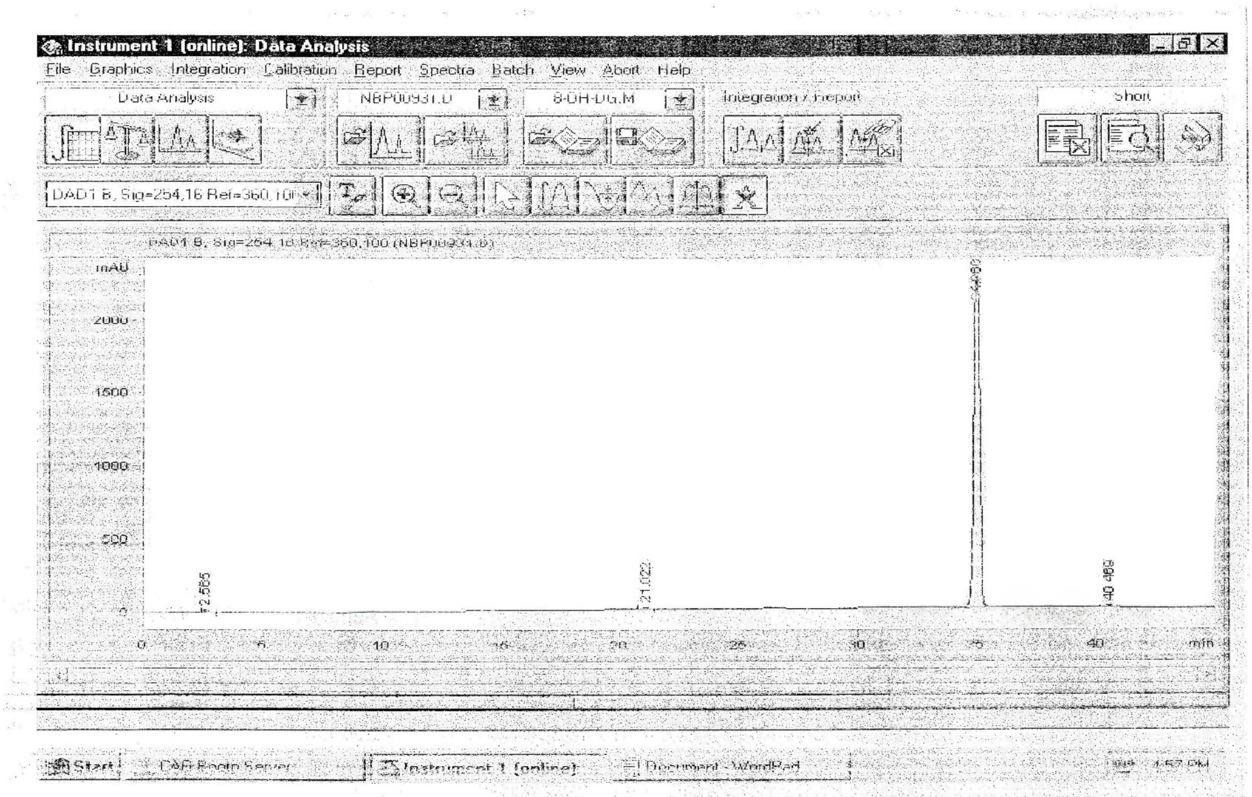

Peak	Ret Time (min)	Width (min)	Area (mAU*s)	Height (mAU)	Area %
1	2.565	0.1737	235.33588	18.84649	0.5583
2	21.022	0.2402	228.44855	14.46945	0.5419
3	34.966	0.2839	4.11997e4	2268.51587	97.7365
4	40.469	0.4871	490.38080	14.33652	1.1633

Figure S19. HPLC chromatogram of compound **1**.

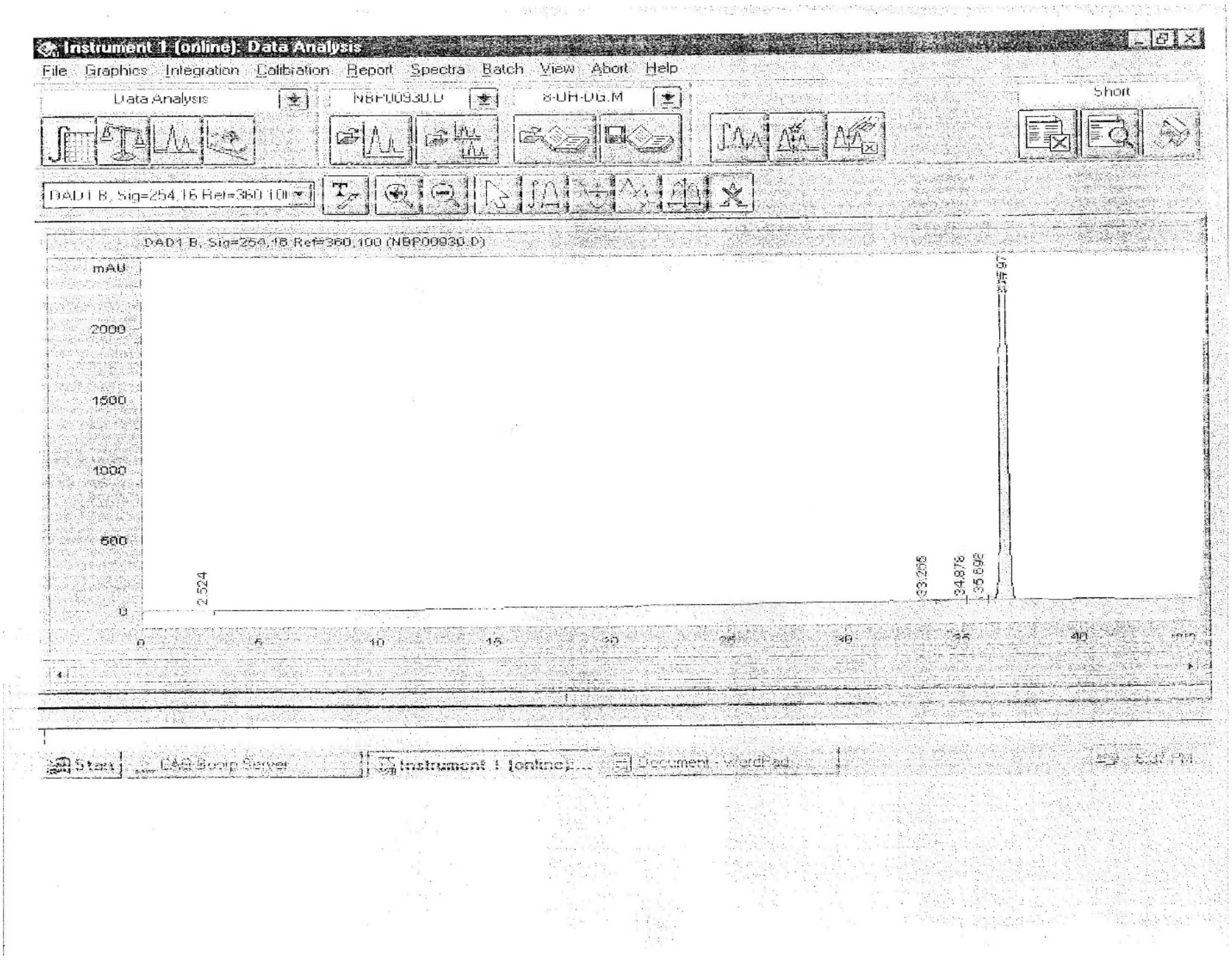

Peak	Ret Time (min)	Width (min)	Area (mAU*s)	Height (mAU)	Area %
1	2.524	0.2013	125.97157	8.48029	0.2180
2	33.255	0.8754	618.05225	8.80022	1.0697
3	34.878	0.8646	517.92682	7.78091	0.8964
4	35.698	0.4027	508.57611	17.51799	0.802
5	36.797	0.3990	5.60084e4	2325.20679	96.9357

Figure S20. HPLC chromatogram of compound **3**.

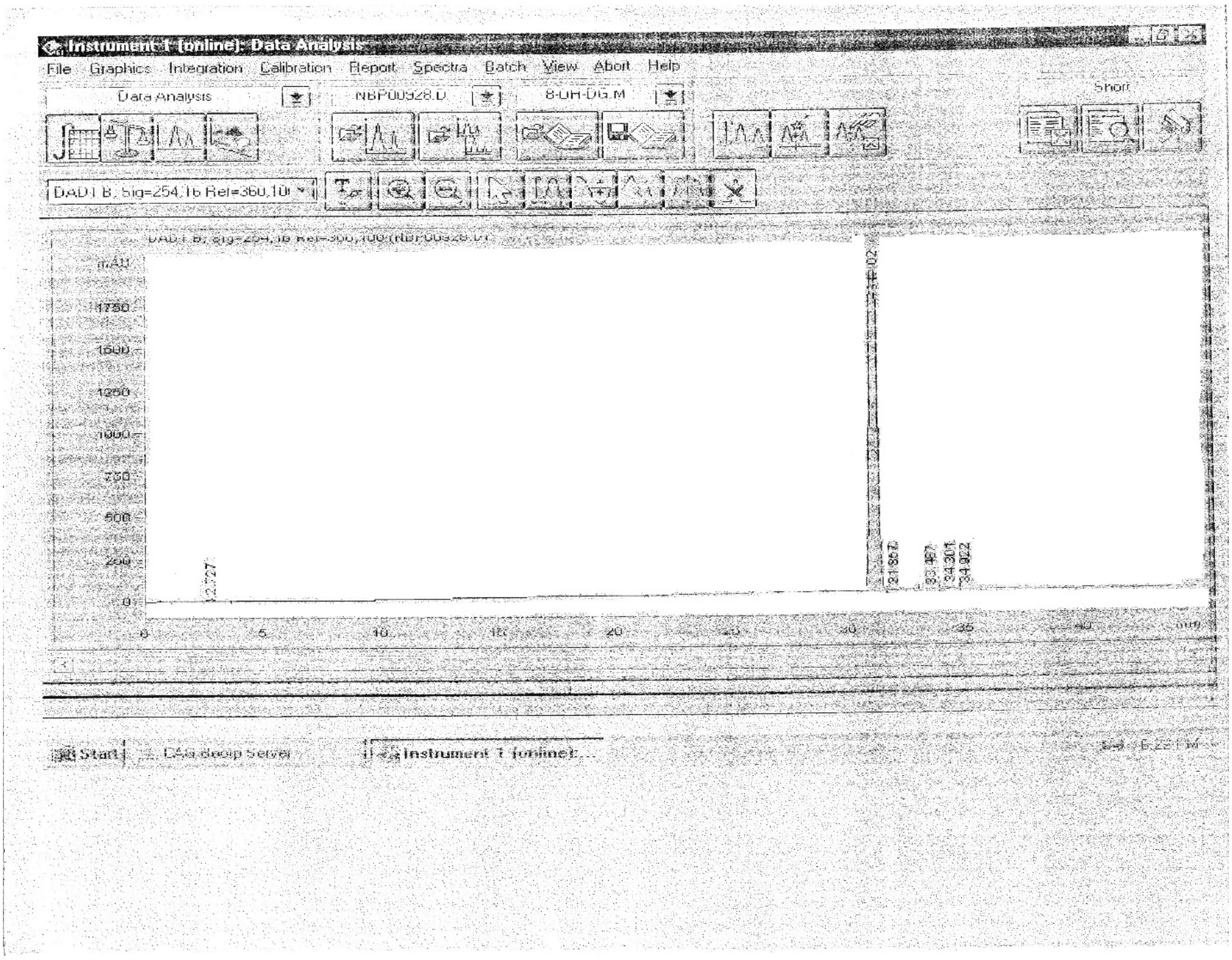

Peak	Ret Time (min)	Width (min)	Area (mAU*s)	Height (mAU)	Area %
1	2.727	0.5115	635.50903	15.46324	1.3904
2	31.102	0.3095	4.36725e4	1919.15308	95.5481
3	31.857	0.5726	324.30182	8.35929	0.7095
4	33.487	0.6173	248.69046	5.36024	0.5441
5	34.301	0.3866	409.17001	14.61705	0.8952
6	34.922	0.8061	417.17401	6.77763	0.9127

Figure S21. HPLC chromatogram of compound **4**.

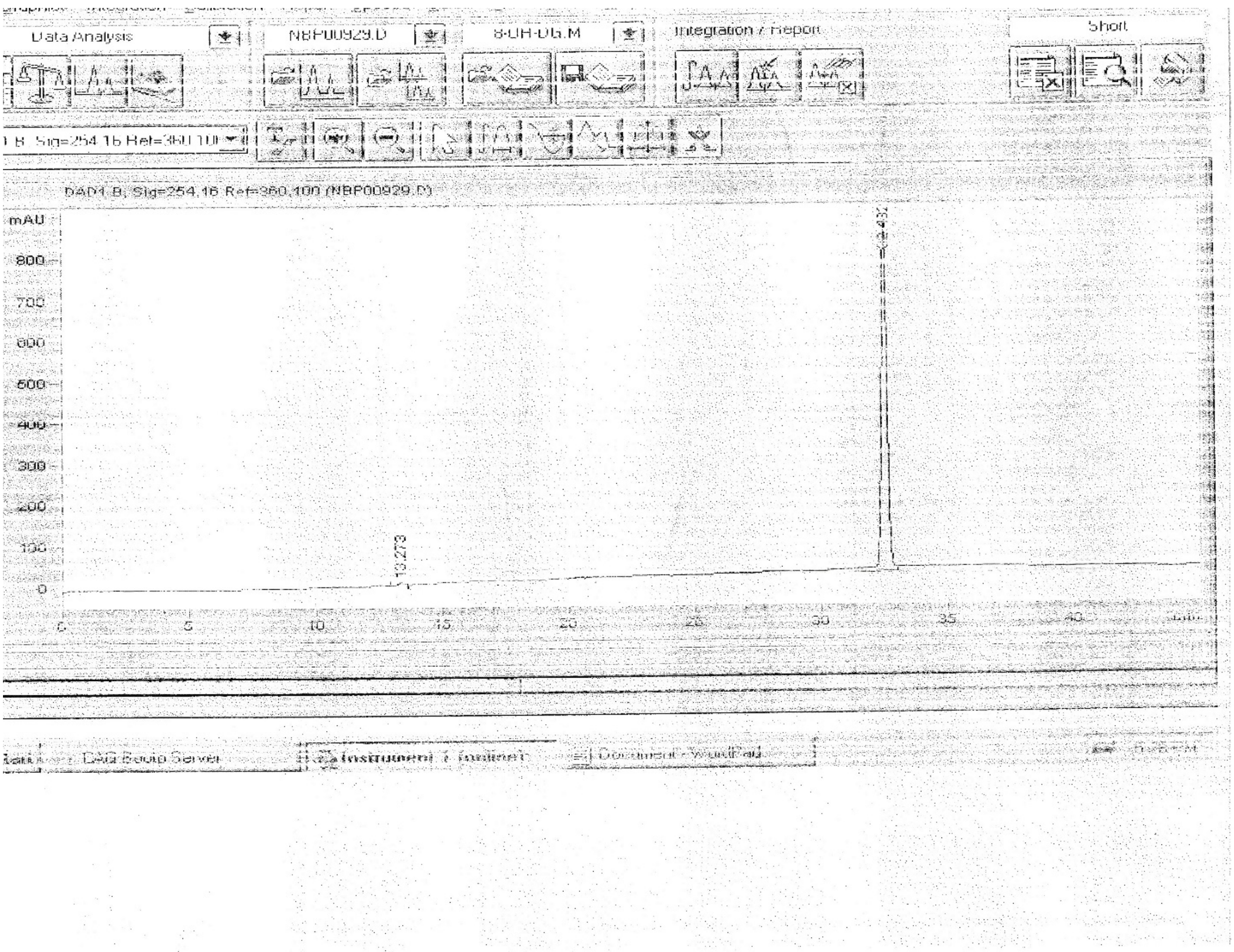

Peak	Ret Time (min)	Width (min)	Area (mAU*s)	Height (mAU)	Area %
1	13.273	0.2893	116.18382	6.12624	0.8648
2	32.482	0.2130	1.33193e4	849.00500	99.1352

Figure S22. HPLC chromatogram of compound **5**.

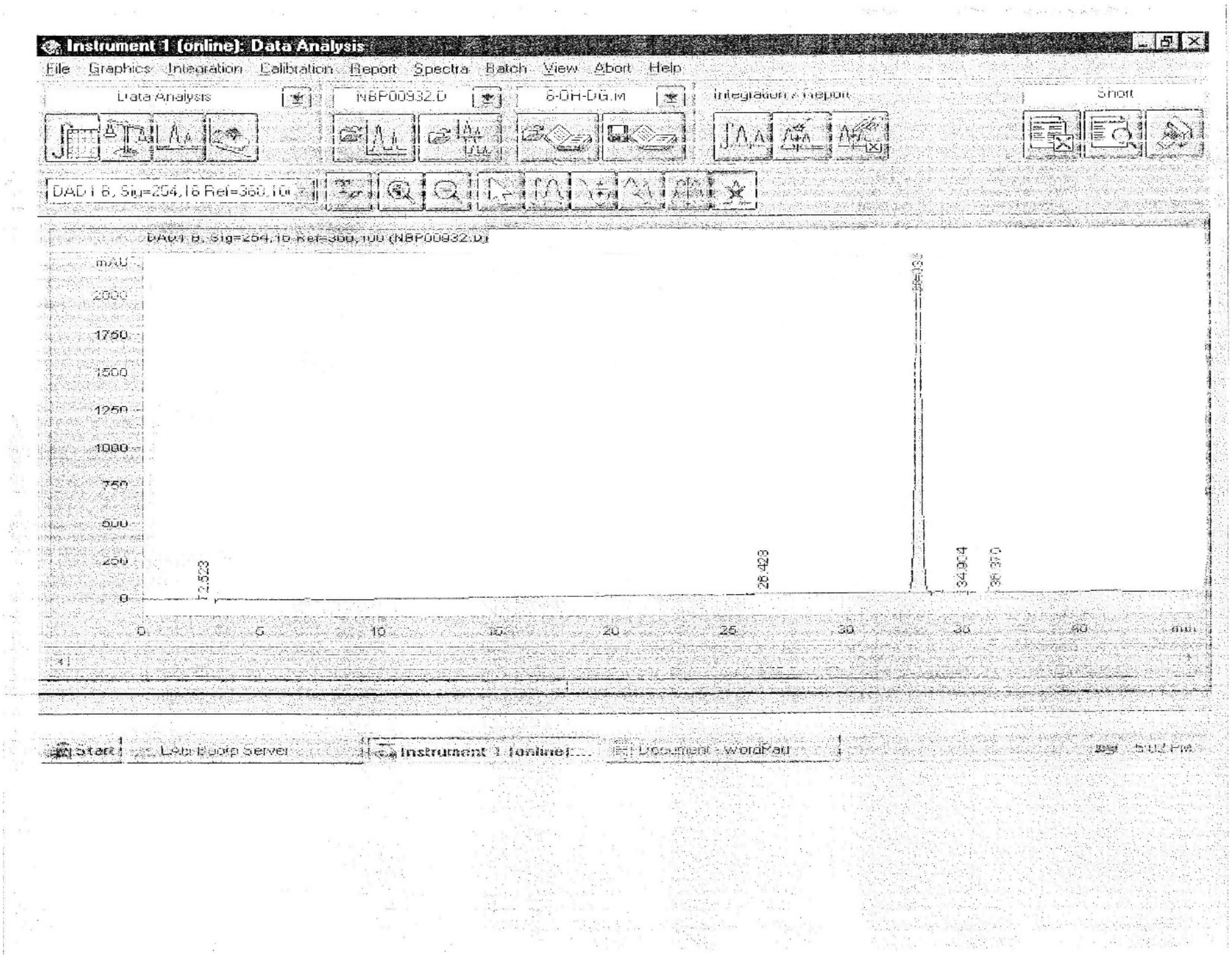

Peak	Ret Time (min)	Width (min)	Area (mAU*s)	Height (mAU)	Area %
1	2.523	0.1840	190.62079	14.25252	0.4663
2	26.428	0.1274	431.77814	4.80940	1.0563
3	33.035	0.2966	3.96705e4	2136.78589	97.0477
4	34.904	0.7126	349.49069	6.36453	0.8550
5	36.370	0.5087	234.94179	6.22020	0.5747

Figure S23. HPLC chromatogram of compound **6**.

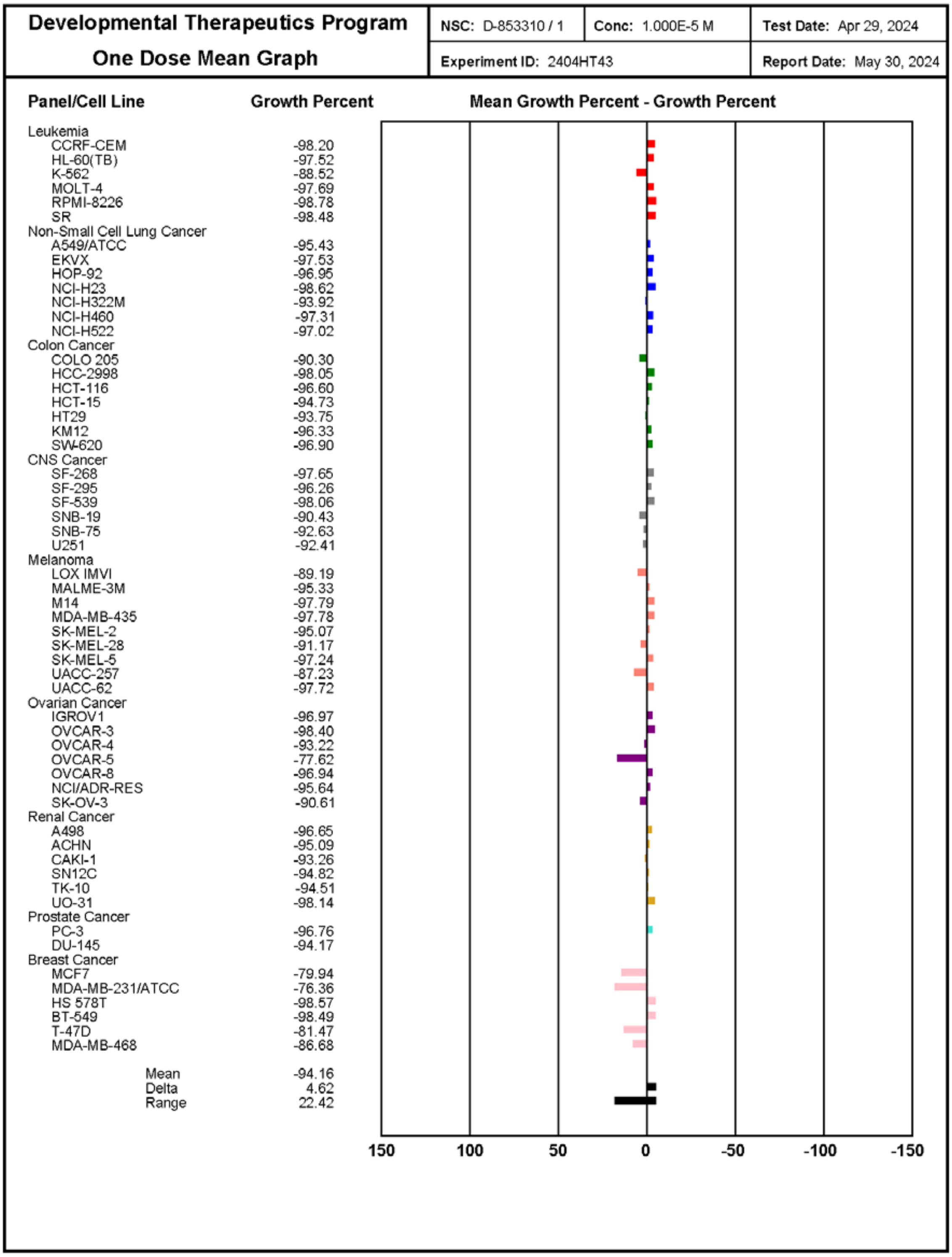

Figure S24. NCI-60 results at one dose (10 µM) of compound **1** (NSC 853310) after 48 h of treatment.

National Cancer Institute Developmental Therapeutics Program
In-Vitro Testing Results

NSC : D - 853310 / 1	Experiment ID : 2407HT60	Test Type : HTS	Units : Molar
Report Date : December 4, 2024	Test Date : July 29, 2024	QNS :	MC :
COMI : C26	Stain Reagent :	SSPL : 0ZVG	

Log10 Concentration

	Time		Mean Optical Densities					Percent Growth							
Panel/Cell Line	Zero	Ctrl	-8.0	-7.0	-6.0	-5.0	-4.0	-8.0	-7.0	-6.0	-5.0	-4.0	GI50	TGI	LC50
Leukemia															
CCRF-CEM	0.676	4.286	4.510	4.605	2.657	0.014	0.003	106	109	55	-98	-100	* 1.07E-6	* 2.28E-6	* 4.85E-6
HL-60(TB)	0.587	2.824	3.500	3.902	0.037	0.007	0.000	131	149	-94	-99	-100	* 2.56E-7	* 4.11E-7	* 6.60E-7
K-562	0.506	7.390	7.469	8.253	6.901	0.086	0.006	101	113	93	-83	-99	* 1.75E-6	* 3.37E-6	* 6.49E-6
MOLT-4	0.700	3.609	4.114	4.169	1.581	0.010	0.001	117	119	30	-98	-100	* 6.01E-7	* 1.72E-6	* 4.20E-6
RPMI-8226	4.400	10.261	11.940	12.291	10.592	0.032	0.004	129	135	106	-99	-100	* 1.87E-6	* 3.28E-6	* 5.75E-6
SR	0.057	0.620	0.653	0.661	0.207	0.002	0.001	106	107	27	-96	-99	* 5.12E-7	* 1.64E-6	* 4.20E-6
Non-Small Cell Lung Cancer															
A549/ATCC	0.612	4.388	4.596	4.525	4.347	0.033	0.003	106	104	99	-95	-100	* 1.79E-6	* 3.25E-6	* 5.88E-6
EKVX	2.594	7.333	8.818	8.742	5.720	0.241	0.014	131	130	66	-91	-100	* 1.27E-6	* 2.64E-6	* 5.50E-6
HOP-62	1.049	3.448	3.900	3.998	2.523	0.025	0.007	119	123	62	-98	-99	* 1.18E-6	* 2.43E-6	* 5.02E-6
HOP-92	8.768	10.268	11.184	11.688	10.892	0.144	0.019	164	200	142	-98	-100	* 2.41E-6	* 3.90E-6	* 6.29E-6
NCI-H226	6.508	13.383	14.986	14.837	14.592	0.106	0.012	123	121	118	-98	-100	* 2.06E-6	* 3.50E-6	* 5.97E-6
NCI-H23	4.317	11.185	12.064	10.982	6.480	0.147	0.018	113	97	32	-97	-100	* 5.22E-7	* 1.76E-6	* 4.33E-6
NCI-H322M	4.446	11.942	13.559	13.414	12.359	0.689	0.039	122	120	106	-84	-99	* 1.96E-6	* 3.59E-6	* 6.58E-6
NCI-H460	1.246	18.944	19.893	20.950	11.880	0.096	0.031	105	111	60	-92	-98	* 1.16E-6	* 2.48E-6	* 5.28E-6
NCI-H522	3.641	9.201	9.464	9.741	8.118	0.249	0.007	105	110	80	-93	-100	* 1.50E-6	* 2.91E-6	* 5.64E-6
Colon Cancer															
COLO 205	1.187	4.714	5.313	5.508	5.718	0.571	0.010	117	122	128	-52	-99	* 2.72E-6	* 5.15E-6	* 9.76E-6
HCC-2998	1.982	5.441	5.830	7.623	5.836	0.293	0.010	111	163	111	-85	-100	* 2.05E-6	* 3.69E-6	* 6.62E-6
HCT-116	0.434	4.709	4.746	4.906	3.101	0.019	0.005	101	105	62	-96	-99	* 1.20E-6	* 2.48E-6	* 5.15E-6
HCT-15	1.389	11.919	11.343	15.016	10.140	0.146	0.012	94	129	83	-90	-99	* 1.56E-6	* 3.03E-6	* 5.91E-6
HT29	0.630	5.226	5.373	5.566	5.323	0.091	0.008	103	107	102	-86	-99	* 1.90E-6	* 3.50E-6	* 6.47E-6
KM12	0.537	3.106	3.540	3.592	3.705	0.142	0.006	117	119	123	-74	-99	* 2.36E-6	* 4.23E-6	* 7.59E-6
SW-620	0.598	4.200	4.953	4.872	4.767	0.021	0.008	121	119	116	-96	-99	* 2.04E-6	* 3.51E-6	* 6.04E-6
CNS Cancer															
SF-268	0.654	2.157	2.328	2.201	1.587	0.041	0.004	111	103	62	-94	-99	* 1.20E-6	* 2.50E-6	* 5.24E-6
SF-295	1.921	4.564	4.536	4.595	4.344	0.120	0.010	99	101	92	-94	-100	* 1.68E-6	* 3.12E-6	* 5.81E-6
SF-539	3.656	12.884	14.044	13.691	9.052	0.056	0.024	113	109	58	-98	-99	* 1.13E-6	* 2.36E-6	* 4.91E-6
SNB-19	1.925	4.811	5.169	5.251	4.736	0.377	0.012	112	115	97	-80	-99	* 1.85E-6	* 3.53E-6	* 6.74E-6
SNB-75	1.257	2.396	2.497	2.540	1.762	0.163	0.005	109	113	44	-87	-100	* 8.25E-7	* 2.17E-6	* 5.22E-6
U251	0.750	4.047	4.598	4.562	3.438	0.081	0.006	117	116	82	-89	-99	* 1.53E-6	* 3.00E-6	* 5.90E-6
Melanoma															
LOX IMVI	0.607	3.513	3.830	4.102	2.390	0.065	0.007	111	120	61	-89	-99	* 1.19E-6	* 2.55E-6	* 5.49E-6
MALME-3M	6.325	8.944	9.065	9.586	8.017	0.611	0.024	105	124	64	-90	-100	* 1.24E-6	* 2.61E-6	* 5.49E-6
M14	4.271	11.958	13.021	13.110	9.601	0.103	0.027	114	115	69	-98	-99	* 1.31E-6	* 2.60E-6	* 5.19E-6
MDA-MB-435	1.473	4.874	5.373	5.386	4.776	0.043	0.013	115	115	97	-97	-99	* 1.75E-6	* 3.16E-6	* 5.72E-6
SK-MEL-2	2.519	5.550	5.057	5.661	4.490	0.199	0.012	84	104	65	-92	-100	* 1.25E-6	* 2.60E-6	* 5.40E-6
SK-MEL-28	1.431	3.164	3.416	3.649	3.140	0.165	0.003	115	128	99	-88	-100	* 1.82E-6	* 3.37E-6	* 6.23E-6
SK-MEL-5	3.825	11.459	11.663	12.137	10.676	0.139	0.025	103	109	90	-96	-99	* 1.63E-6	* 3.03E-6	* 5.63E-6
UACC-257	3.486	7.298	7.716	8.348	5.009	0.531	0.006	111	128	40	-85	-100	* 7.67E-7	* 2.09E-6	* 5.26E-6
UACC-62	0.739	3.049	3.276	3.213	2.247	0.010	0.002	110	107	65	-99	-100	* 1.24E-6	* 2.50E-6	* 5.04E-6
Ovarian Cancer															
IGROV1	1.018	3.394	3.833	4.075	3.235	0.193	0.009	118	129	93	-81	-99	* 1.77E-6	* 3.43E-6	* 6.64E-6
OVCAR-3	4.654	18.685	20.384	20.767	14.110	0.085	0.027	112	115	67	-98	-99	* 1.27E-6	* 2.55E-6	* 5.12E-6
OVCAR-4	5.328	9.372	10.816	10.602	8.683	1.365	0.020	136	130	83	-74	-100	* 1.62E-6	* 3.37E-6	* 7.00E-6
OVCAR-5	6.474	14.805	15.562	15.813	16.066	1.569	0.017	109	112	115	-76	-100	* 2.20E-6	* 4.01E-6	* 7.33E-6
OVCAR-8	0.673	3.725	4.165	4.119	3.329	0.034	0.007	114	113	87	-95	-99	* 1.60E-6	* 3.01E-6	* 5.67E-6
NCI/ADR-RES	3.746	10.491	12.178	12.380	11.267	0.390	0.019	125	128	111	-90	-100	* 2.02E-6	* 3.58E-6	* 6.36E-6
SK-OV-3	2.611	9.797	9.969	10.088	9.806	0.472	0.017	102	104	100	-82	-99	* 1.88E-6	* 3.55E-6	* 6.68E-6
Renal Cancer															
786-0	1.778	6.495	6.367	6.203	5.766	0.384	0.009	97	94	84	-78	-100	* 1.63E-6	* 3.30E-6	* 6.69E-6
A498	1.927	7.756	8.655	8.692	4.633	0.153	0.015	116	116	46	-92	-99	* 8.89E-7	* 2.16E-6	* 4.97E-6
ACHN	1.537	6.486	6.466	6.565	6.590	0.415	0.011	100	102	102	-73	-99	* 1.98E-6	* 3.83E-6	* 7.39E-6
CAKI-1	1.045	4.167	4.454	4.642	3.262	0.265	0.007	109	115	71	-75	-99	* 1.39E-6	* 3.07E-6	* 6.77E-6
RXF 393	1.802	3.005	3.381	3.468	2.827	0.110	0.008	131	139	85	-94	-100	* 1.57E-6	* 2.99E-6	* 5.69E-6
SN12C	0.623	2.418	2.680	2.689	2.317	0.047	0.006	115	115	94	-92	-99	* 1.73E-6	* 3.20E-6	* 5.93E-6
TK-10	6.290	16.819	17.954	17.900	17.227	0.453	0.016	111	110	104	-93	-100	* 1.88E-6	* 3.37E-6	* 6.06E-6
UO-31	1.550	5.879	5.941	6.141	5.040	0.031	0.009	101	106	81	-98	-99	* 1.48E-6	* 2.83E-6	* 5.39E-6
Prostate Cancer															
PC-3	3.978	11.351	13.233	13.223	9.516	0.298	0.015	125	126	75	-92	-100	* 1.41E-6	* 2.80E-6	* 5.58E-6
DU-145	0.636	3.759	3.956	4.040	3.396	0.091	0.007	106	109	88	-86	-99	* 1.66E-6	* 3.22E-6	* 6.23E-6
Breast Cancer															
MCF7	2.550	15.066	12.570	13.157	11.480	0.881	0.009	80	85	71	-66	-100	* 1.43E-6	* 3.33E-6	* 7.71E-6
MDA-MB-231/ATCC	5.261	12.471	13.201	13.182	13.437	1.272	0.019	110	110	113	-76	-100	* 2.16E-6	* 3.97E-6	* 7.30E-6
HS 578T	1.006	2.714	2.778	2.866	1.618	0.025	0.005	104	109	36	-98	-100	* 6.43E-7	* 1.86E-6	* 4.41E-6
BT-549	3.822	8.044	9.590	9.650	8.364	0.055	0.010	137	138	108	-99	-100	* 1.91E-6	* 3.33E-6	* 5.82E-6
T-47D	5.838	11.540	11.277	11.248	10.571	3.660	0.028	95	95	83	-37	-100	* 1.88E-6	* 4.90E-6	* 1.60E-5
MDA-MB-468	6.310	10.749	10.505	10.928	10.012	1.497	0.015	94	104	84	-76	-100	* 1.62E-6	* 3.33E-6	* 6.85E-6

Figure S25. NCI's DTP dose-response report for compound **1**. GI_{50}, TGI and LC_{50} values (expressed as molarity).

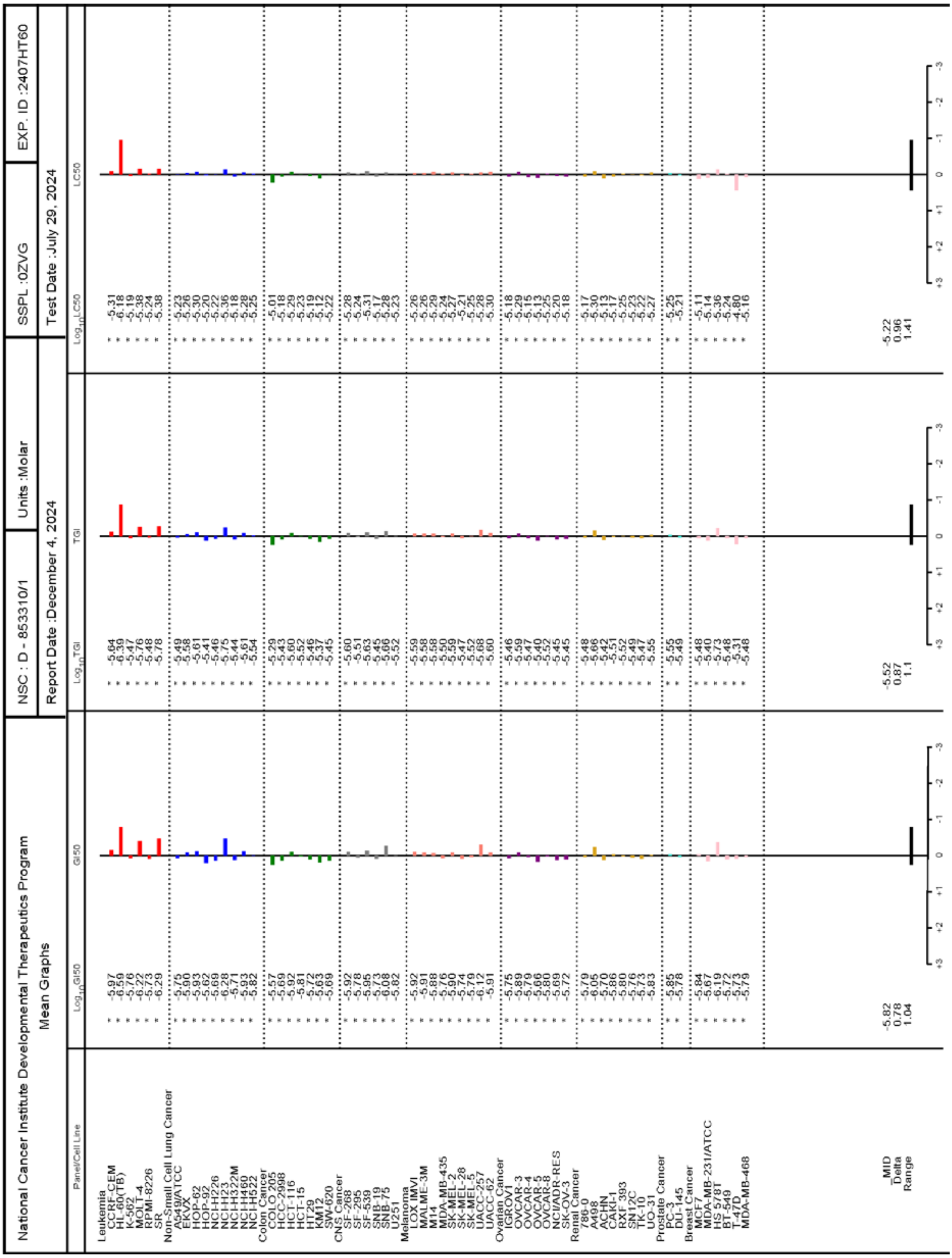

Figure S26. NCI's DTP dose-response report for compound **1**. Mean graphs of GI_{50}, TGI and LC_{50} values.

A)

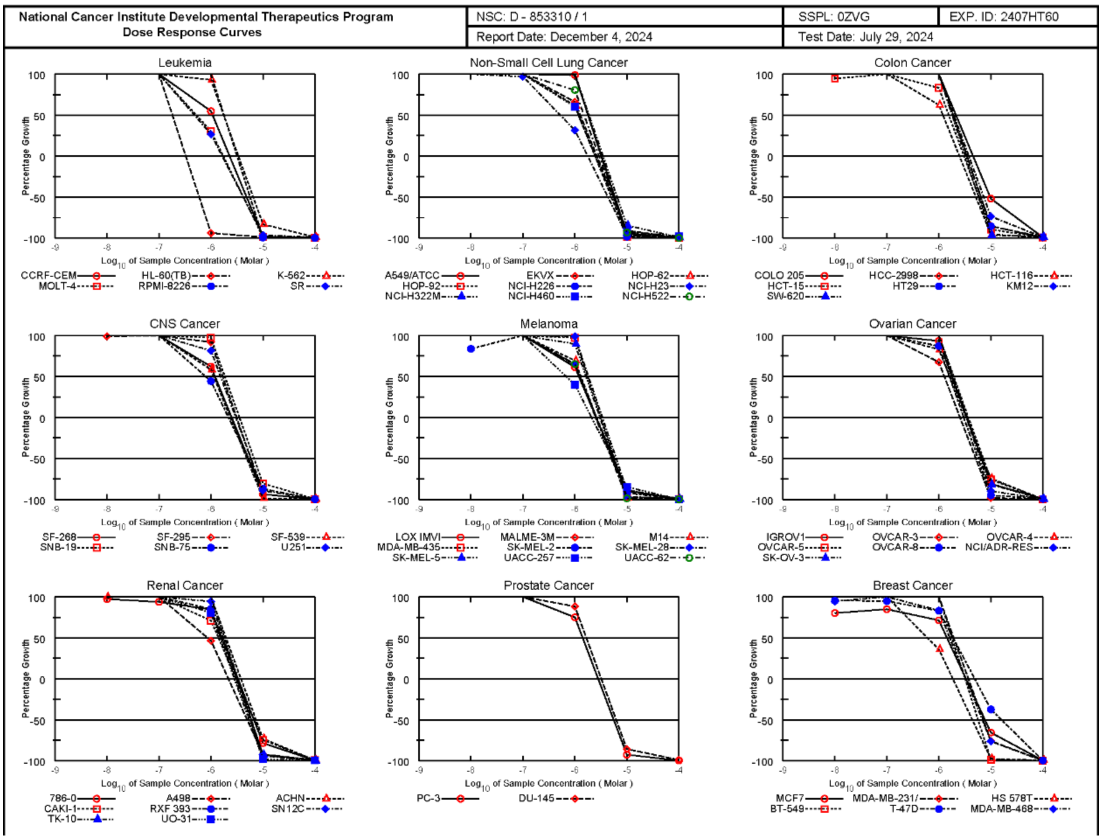

B)

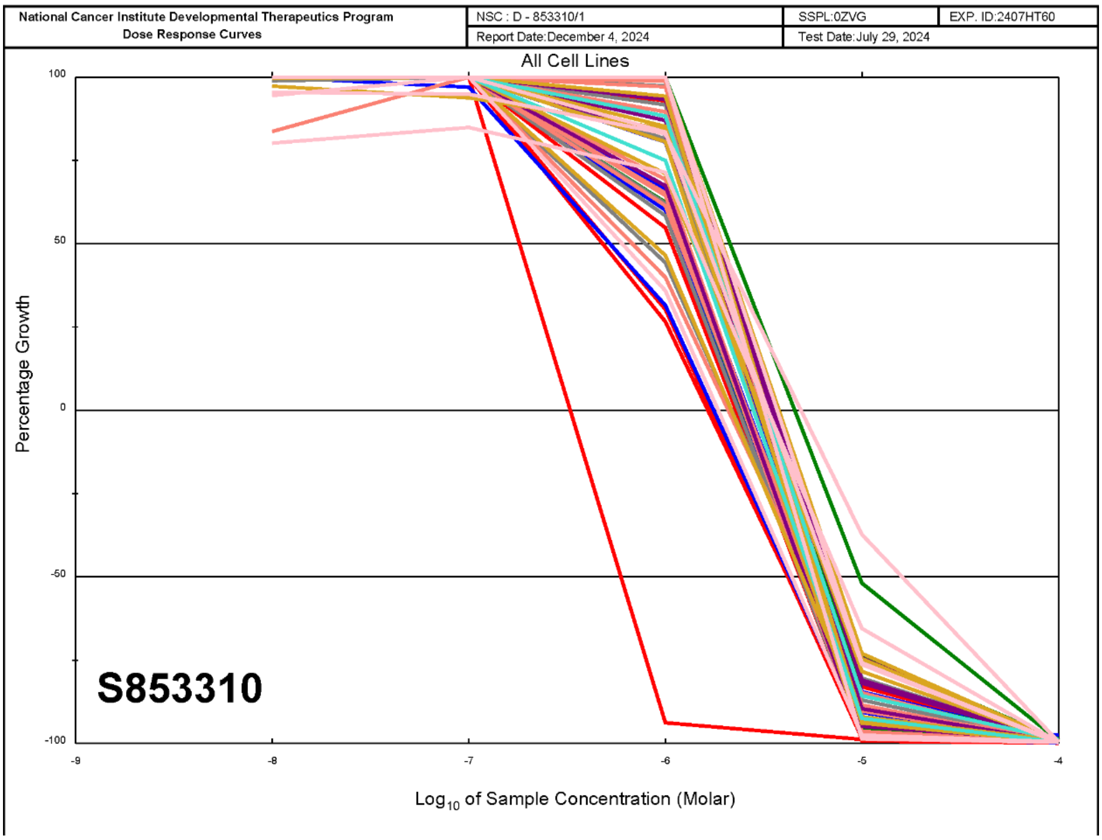

Figure S27. Dose-response curves of the NCI analysis for **1**. (**A**) Curves grouped in nine subpanels derived from different cancer types. (**B**) Curves obtained for all the cancer cell lines tested.

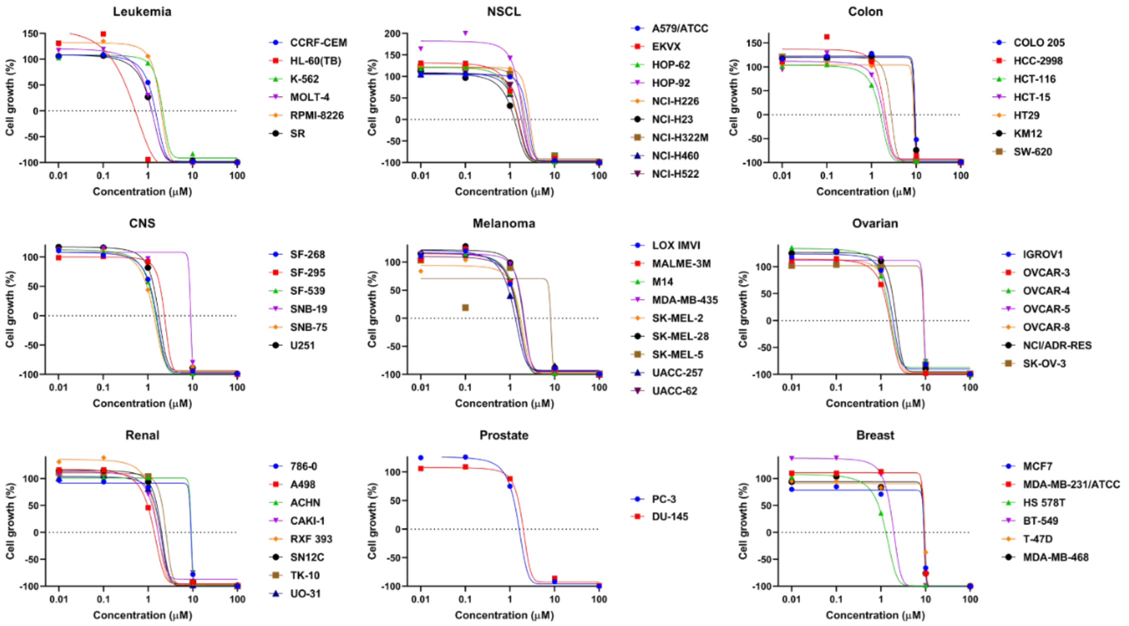

Figure S28. Dose-response curves of **1** in all the cell lines in the NCI panel.

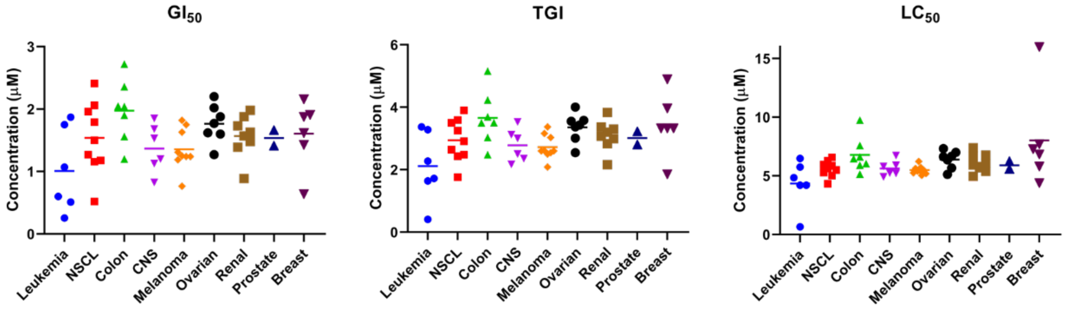

Figure S29. Mean graphs of the GI_{50}, TGI, and LC_{50} values of **1**.

Developmental Therapeutics Program One Dose Mean Graph	NSC: D-853311 / 1	Conc: 1.000E-5 M	Test Date: Apr 29, 2024
	Experiment ID: 2404HT43		Report Date: May 30, 2024

Panel/Cell Line	Growth Percent
**Leukemia**	
CCRF-CEM	-98.28
HL-60(TB)	-97.95
K-562	-76.90
MOLT-4	-97.84
RPMI-8226	-98.68
SR	-98.48
**Non-Small Cell Lung Cancer**	
A549/ATCC	-96.44
EKVX	-98.28
HOP-92	-98.28
NCI-H23	-98.59
NCI-H322M	-87.55
NCI-H460	-97.58
NCI-H522	-97.65
**Colon Cancer**	
COLO 205	-8.09
HCC-2998	-92.40
HCT-116	-96.08
HCT-15	-94.60
HT29	-93.65
KM12	-91.43
SW-620	-96.88
**CNS Cancer**	
SF-268	-97.72
SF-295	-96.61
SF-539	-98.22
SNB-19	-89.73
SNB-75	-92.23
U251	-92.03
**Melanoma**	
LOX IMVI	-92.38
MALME-3M	-94.96
M14	-98.60
MDA-MB-435	-96.76
SK-MEL-2	-94.65
SK-MEL-28	-89.74
SK-MEL-5	-97.88
UACC-257	-88.72
UACC-62	-98.09
**Ovarian Cancer**	
IGROV1	-96.52
OVCAR-3	-98.46
OVCAR-4	-95.49
OVCAR-5	-75.53
OVCAR-8	-97.72
NCI/ADR-RES	-97.95
SK-OV-3	-89.19
**Renal Cancer**	
A498	-96.72
ACHN	-96.85
CAKI-1	-96.27
SN12C	-92.89
TK-10	-96.17
UO-31	-98.43
**Prostate Cancer**	
PC-3	-97.19
DU-145	-91.60
**Breast Cancer**	
MCF7	-64.28
MDA-MB-231/ATCC	-79.85
HS 578T	-98.93
BT-549	-98.58
T-47D	-71.18
MDA-MB-468	-86.26
Mean	-91.96
Delta	6.97
Range	90.84

Mean Growth Percent - Growth Percent

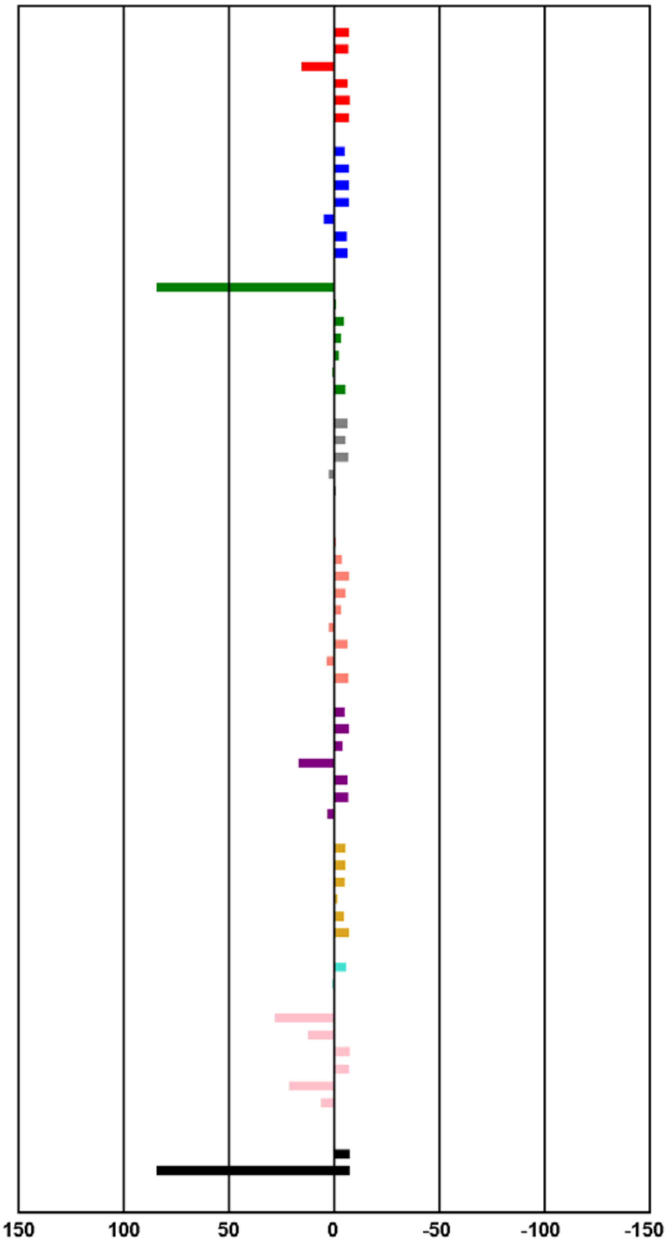

Figure S30. NCI-60 results at one dose (10 µM) of compound **2** (NSC 853311) after 48 h of treatment.

National Cancer Institute Developmental Therapeutics Program<br>In-Vitro Testing Results			
NSC : D - 853311 / 1	Experiment ID : 2407HT60	Test Type : HTS	Units : Molar
Report Date : December 4, 2024	Test Date : July 29, 2024	QNS :	MC :
COMI : C27	Stain Reagent :	SSPL : 0ZVG	

Log10 Concentration

	Time		Mean Optical Densities					Percent Growth							
Panel/Cell Line	Zero	Ctrl	-8.0	-7.0	-6.0	-5.0	-4.0	-8.0	-7.0	-6.0	-5.0	-4.0	GI50	TGI	LC50
Leukemia															
CCRF-CEM	0.676	4.286	4.618	4.568	2.505	0.016	0.011	109	108	51	-98	-98	* 1.01E-6	* 2.20E-6	* 4.77E-6
HL-60(TB)	0.587	2.824	3.169	3.592	0.023	0.012	0.010	115	134	-96	-98	-98	* 2.31E-7	* 3.82E-7	* 6.30E-7
K-562	0.506	7.390	7.988	7.260	5.631	0.123	0.021	109	98	74	-76	-96	* 1.45E-6	* 3.13E-6	* 6.75E-6
MOLT-4	0.700	3.609	4.166	4.228	1.775	0.017	0.013	119	121	37	-98	-98	* 7.00E-7	* 1.88E-6	* 4.43E-6
RPMI-8226	4.400	10.261	11.557	11.242	9.570	0.044	0.025	122	117	88	-99	-99	* 1.60E-6	* 2.96E-6	* 5.48E-6
SR	0.057	0.620	0.665	0.618	0.172	0.002	0.001	108	100	20	-97	-99	* 4.23E-7	* 1.49E-6	* 3.97E-6
Non-Small Cell Lung Cancer															
A549/ATCC	0.612	4.388	4.449	4.612	4.337	0.010	0.003	102	106	99	-98	-100	* 1.77E-6	* 3.17E-6	* 5.68E-6
EKVX	2.594	7.333	9.095	8.140	5.674	0.131	0.015	137	117	65	-95	-99	* 1.24E-6	* 2.55E-6	* 5.24E-6
HOP-62	1.049	3.448	3.892	4.051	2.125	0.017	0.004	118	125	45	-98	-100	* 8.62E-7	* 2.06E-6	* 4.59E-6
HOP-92	8.768	10.268	11.115	11.577	10.506	0.098	0.014	156	190	118	-99	-100	* 2.05E-6	* 3.49E-6	* 5.94E-6
NCI-H226	6.508	13.383	14.857	14.962	13.737	0.056	0.013	121	123	105	-99	-100	* 1.86E-6	* 3.27E-6	* 5.75E-6
NCI-H23	4.317	11.185	11.034	11.313	5.797	0.124	0.014	98	102	22	-97	-100	* 4.42E-7	* 1.52E-6	* 4.01E-6
NCI-H322M	4.446	11.942	13.583	13.658	11.683	0.645	0.034	122	123	97	-86	-99	* 1.80E-6	* 3.39E-6	* 6.38E-6
NCI-H460	1.246	18.944	20.313	19.439	12.951	0.077	0.011	108	103	66	-94	-99	* 1.26E-6	* 2.59E-6	* 5.32E-6
NCI-H522	3.641	9.201	9.394	9.163	7.815	0.082	0.008	104	99	75	-98	-100	* 1.40E-6	* 2.72E-6	* 5.29E-6
Colon Cancer															
COLO 205	1.187	4.714	5.402	5.440	5.809	0.713	0.021	120	121	131	-40	-98	* 2.98E-6	* 5.84E-6	* 1.49E-5
HCC-2998	1.982	5.441	6.170	5.670	7.141	0.402	0.015	121	107	149	-80	-99	* 2.71E-6	* 4.48E-6	* 7.41E-6
HCT-116	0.434	4.709	4.731	5.015	3.951	0.021	0.006	101	107	82	-95	-99	* 1.52E-6	* 2.91E-6	* 5.57E-6
HCT-15	1.389	11.919	9.037	12.336	10.781	0.112	0.041	73	104	89	-92	-97	* 1.65E-6	* 3.11E-6	* 5.87E-6
HT29	0.630	5.226	5.349	5.424	4.703	0.081	0.012	103	104	89	-87	-98	* 1.66E-6	* 3.19E-6	* 6.15E-6
KM12	0.537	3.106	3.497	3.658	3.569	0.168	0.007	115	121	118	-69	-99	* 2.31E-6	* 4.29E-6	* 7.94E-6
SW-620	0.598	4.200	4.731	4.869	4.490	0.022	0.010	115	119	108	-96	-98	* 1.92E-6	* 3.38E-6	* 5.93E-6
CNS Cancer															
SF-268	0.654	2.157	2.311	2.361	1.542	0.018	0.003	110	113	59	-97	-100	* 1.14E-6	* 2.39E-6	* 4.99E-6
SF-295	1.921	4.564	4.773	4.644	4.600	0.092	0.011	108	103	101	-95	-99	* 1.82E-6	* 3.28E-6	* 5.89E-6
SF-539	3.656	12.884	13.609	13.557	6.776	0.041	0.014	108	107	34	-99	-100	* 6.02E-7	* 1.80E-6	* 4.28E-6
SNB-19	1.925	4.811	5.270	5.185	4.689	0.327	0.012	116	113	96	-83	-99	* 1.80E-6	* 3.43E-6	* 6.54E-6
SNB-75	1.257	2.396	2.545	2.438	1.228	0.147	0.003	113	104	-2	-88	-100	* 3.21E-7	* 9.51E-7	* 3.59E-6
U251	0.750	4.047	4.648	4.558	3.107	0.086	0.004	118	115	72	-89	-99	* 1.36E-6	* 2.80E-6	* 5.74E-6
Melanoma															
LOX IMVI	0.607	3.513	3.962	4.000	1.584	0.052	0.002	115	117	34	-91	-100	* 6.35E-7	* 1.86E-6	* 4.67E-6
MALME-3M	6.325	8.944	9.240	9.327	7.478	0.809	0.016	112	115	44	-87	-100	* 8.27E-7	* 2.17E-6	* 5.21E-6
M14	4.271	11.958	12.888	12.555	9.658	0.066	0.017	112	108	70	-98	-100	* 1.32E-6	* 2.60E-6	* 5.16E-6
MDA-MB-435	1.473	4.874	5.472	5.370	4.933	0.047	0.013	118	115	102	-97	-99	* 1.82E-6	* 3.25E-6	* 5.81E-6
SK-MEL-2	2.519	5.550	5.495	5.394	4.295	0.185	0.003	98	95	59	-93	-100	* 1.14E-6	* 2.44E-6	* 5.22E-6
SK-MEL-28	1.431	3.164	3.541	3.498	3.333	0.247	0.003	122	119	110	-83	-100	* 2.04E-6	* 3.72E-6	* 6.76E-6
SK-MEL-5	3.825	11.459	11.047	11.265	9.483	0.133	0.011	94	98	74	-96	-100	* 1.39E-6	* 2.72E-6	* 5.34E-6
UACC-257	3.486	7.298	8.209	8.330	4.504	0.561	0.006	124	127	27	-84	-100	* 5.85E-7	* 1.74E-6	* 4.93E-6
UACC-62	0.739	3.049	3.264	3.261	2.485	0.010	0.001	109	109	76	-99	-100	* 1.40E-6	* 2.72E-6	* 5.26E-6
Ovarian Cancer															
IGROV1	1.018	3.394	3.841	3.922	3.115	0.095	0.007	119	122	88	-91	-99	* 1.64E-6	* 3.11E-6	* 5.93E-6
OVCAR-3	4.654	18.685	20.083	19.743	12.162	0.103	0.035	110	108	54	-98	-99	* 1.06E-6	* 2.26E-6	* 4.83E-6
OVCAR-4	5.328	9.372	10.721	10.926	8.208	0.503	0.012	133	138	71	-91	-100	* 1.35E-6	* 2.76E-6	* 5.61E-6
OVCAR-5	6.474	14.805	14.503	15.057	15.276	0.922	0.028	96	103	106	-86	-100	* 1.95E-6	* 3.56E-6	* 6.50E-6
OVCAR-8	0.673	3.725	4.033	4.012	3.145	0.015	0.002	110	109	81	-98	-100	* 1.49E-6	* 2.84E-6	* 5.41E-6
NCI/ADR-RES	3.746	10.491	12.470	12.182	11.070	0.181	0.030	129	125	109	-95	-99	* 1.94E-6	* 3.41E-6	* 6.00E-6
SK-OV-3	2.611	9.797	9.943	10.567	8.387	0.539	0.009	102	111	80	-79	-100	* 1.55E-6	* 3.19E-6	* 6.55E-6
Renal Cancer															
786-0	1.778	6.495	6.322	6.268	5.871	0.228	0.007	96	95	87	-87	-100	* 1.63E-6	* 3.15E-6	* 6.11E-6
A498	1.927	7.756	8.621	8.043	4.498	0.110	0.005	115	105	44	-94	-100	* 7.99E-7	* 2.08E-6	* 4.79E-6
ACHN	1.537	6.486	6.485	6.518	6.519	0.321	0.011	100	101	101	-79	-99	* 1.91E-6	* 3.63E-6	* 6.89E-6
CAKI-1	1.045	4.167	4.634	4.563	2.486	0.105	0.005	115	113	46	-90	-100	* 8.76E-7	* 2.18E-6	* 5.09E-6
RXF 393	1.802	3.005	3.309	3.331	2.569	0.062	0.004	125	127	64	-97	-100	* 1.22E-6	* 2.50E-6	* 5.12E-6
SN12C	0.623	2.418	2.576	2.706	1.988	0.031	0.004	109	116	76	-95	-99	* 1.42E-6	* 2.78E-6	* 5.46E-6
TK-10	6.290	16.819	17.328	17.326	16.962	0.241	0.013	105	105	101	-96	-100	* 1.82E-6	* 3.26E-6	* 5.84E-6
UO-31	1.550	5.879	6.010	5.899	4.803	0.025	0.012	103	100	75	-98	-99	* 1.40E-6	* 2.71E-6	* 5.26E-6
Prostate Cancer															
PC-3	3.978	11.351	12.764	12.853	8.543	0.281	0.012	119	120	62	-93	-100	* 1.19E-6	* 2.51E-6	* 5.28E-6
DU-145	0.636	3.759	4.157	4.006	3.452	0.068	0.007	113	108	90	-89	-99	* 1.68E-6	* 3.18E-6	* 6.04E-6
Breast Cancer															
MCF7	2.550	15.066	14.183	14.033	11.354	1.141	0.017	93	92	70	-55	-99	* 1.45E-6	* 3.63E-6	* 9.08E-6
MDA-MB-231/ATCC	5.261	12.471	13.029	13.085	12.699	1.492	0.017	108	108	103	-72	-100	* 2.01E-6	* 3.89E-6	* 7.52E-6
HS 578T	1.006	2.714	2.367	2.651	0.946	0.019	0.003	80	96	-7	-98	-100	* 2.81E-7	* 8.55E-7	* 2.96E-6
BT-549	3.822	8.044	9.280	9.673	8.057	0.055	0.012	129	138	100	-98	-100	* 1.79E-6	* 3.20E-6	* 5.70E-6
T-47D	5.838	11.540	11.245	11.527	9.843	3.398	0.038	95	100	70	-42	-99	* 1.52E-6	* 4.24E-6	* 1.39E-5
MDA-MB-468	6.310	10.749	10.791	11.101	10.504	1.396	0.027	101	108	94	-78	-100	* 1.81E-6	* 3.53E-6	* 6.89E-6

Figure S31. NCI's DTP dose-response report for compound **2**. GI_{50}, TGI and LC_{50} values (expressed as molarity).

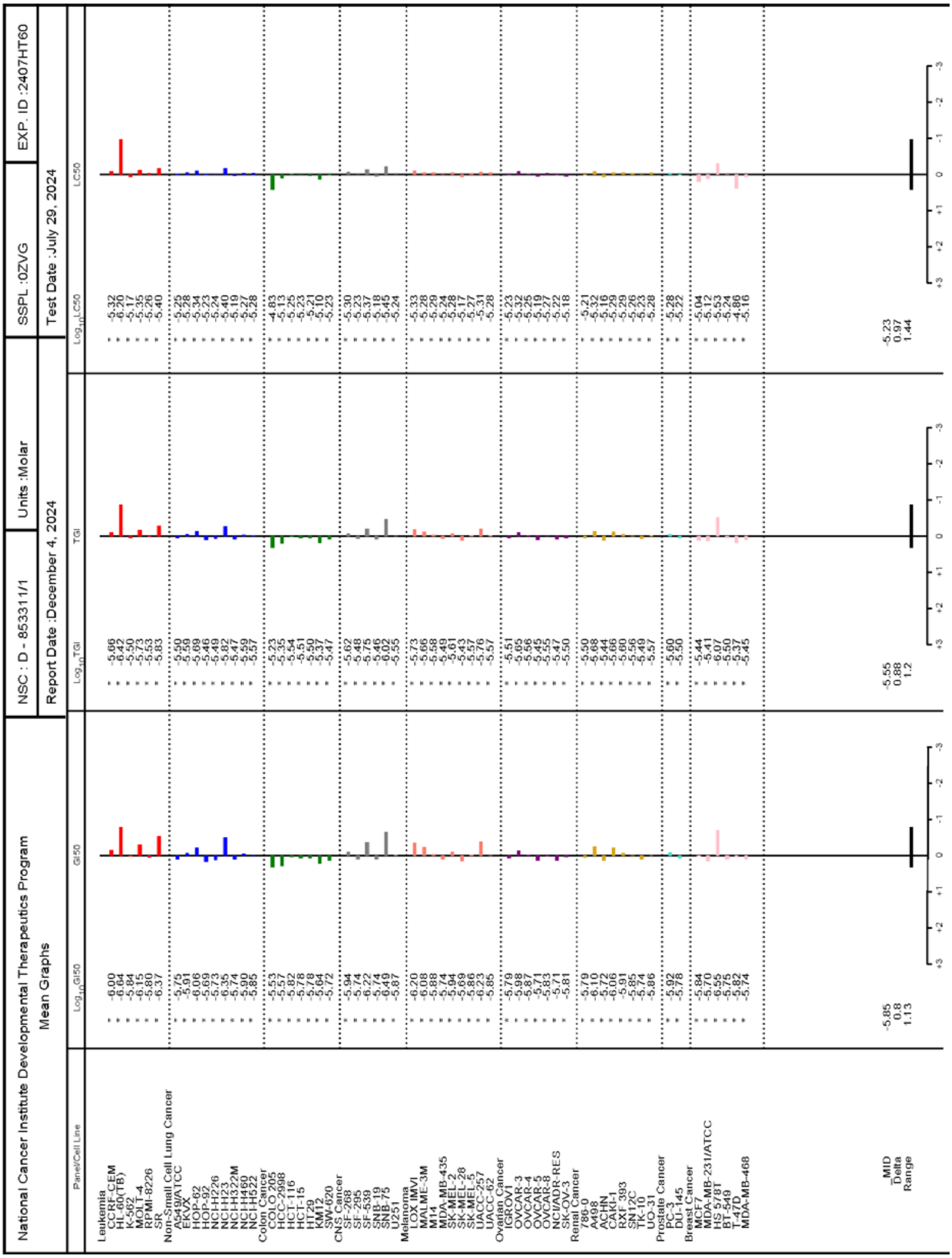

Figure S32. NCI's DTP dose-response report for compound **2**. Mean graphs of GI_{50}, TGI and LC_{50} values.

A)

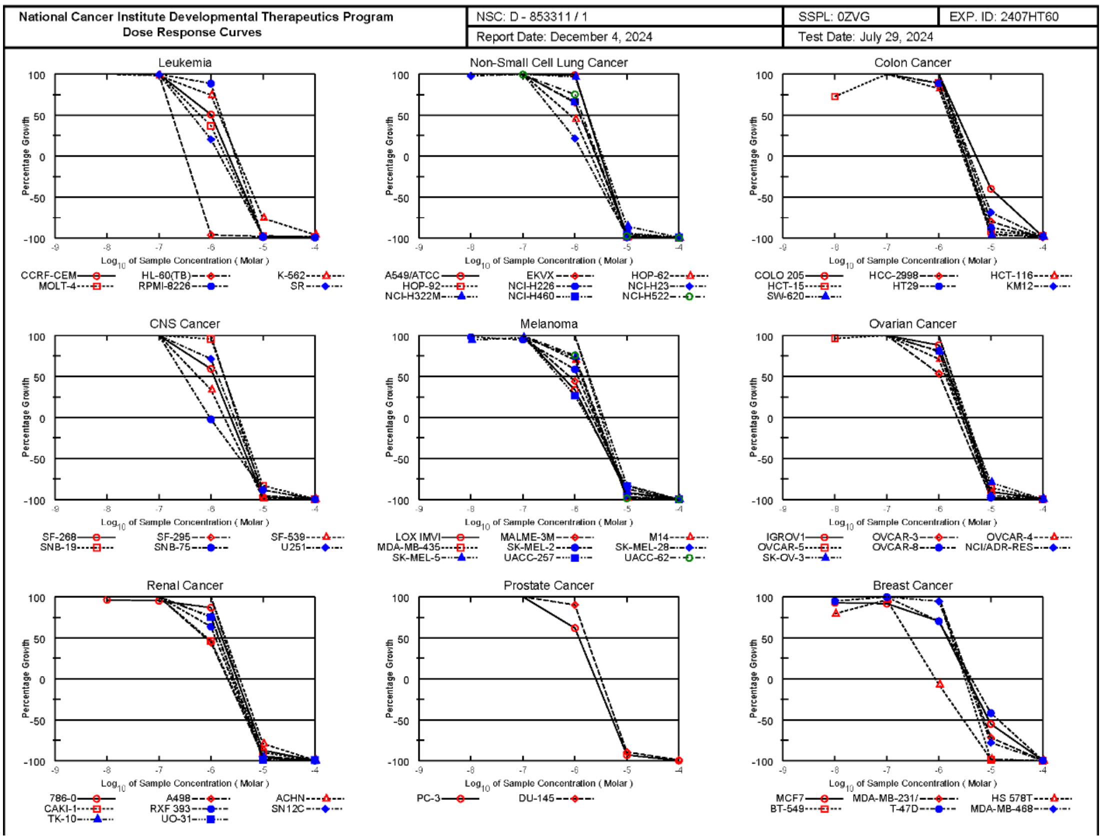

B)

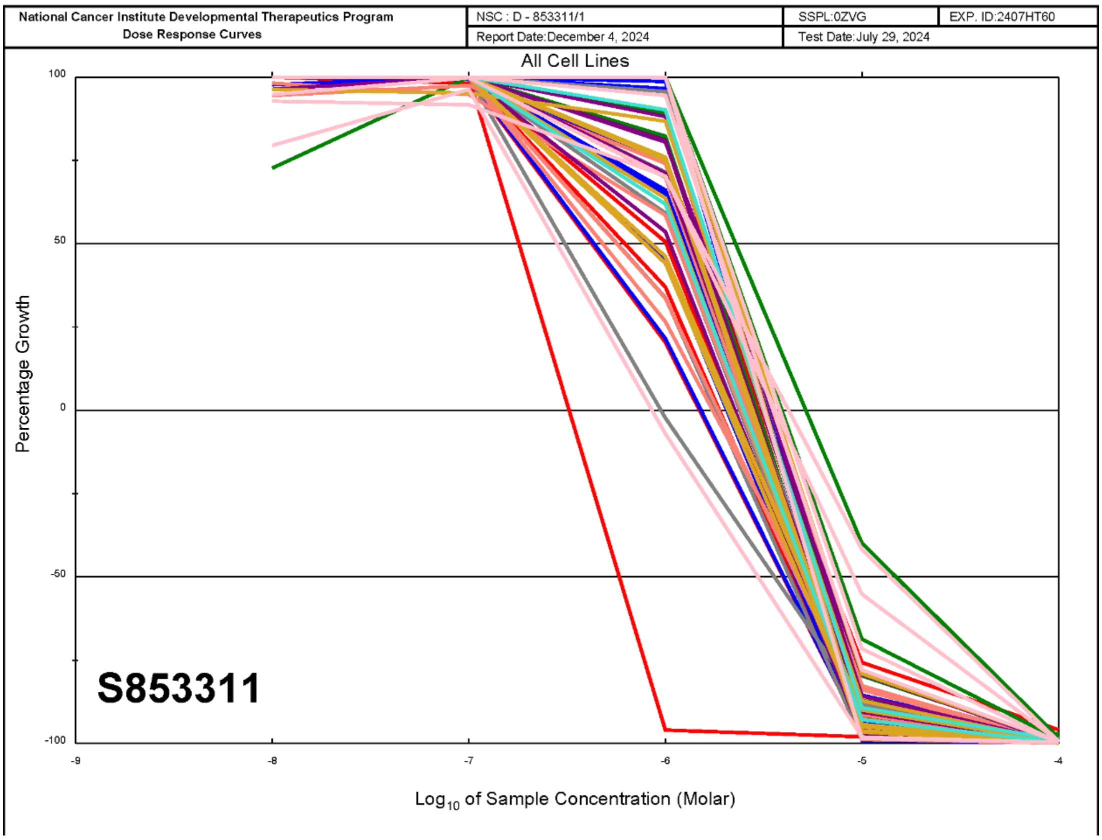

Figure S33. Dose-response curves of the NCI analysis for **2**. (**A**) Curves grouped in nine subpanels derived from different cancer types. (**B**) Curves obtained for all the cancer cell lines tested.

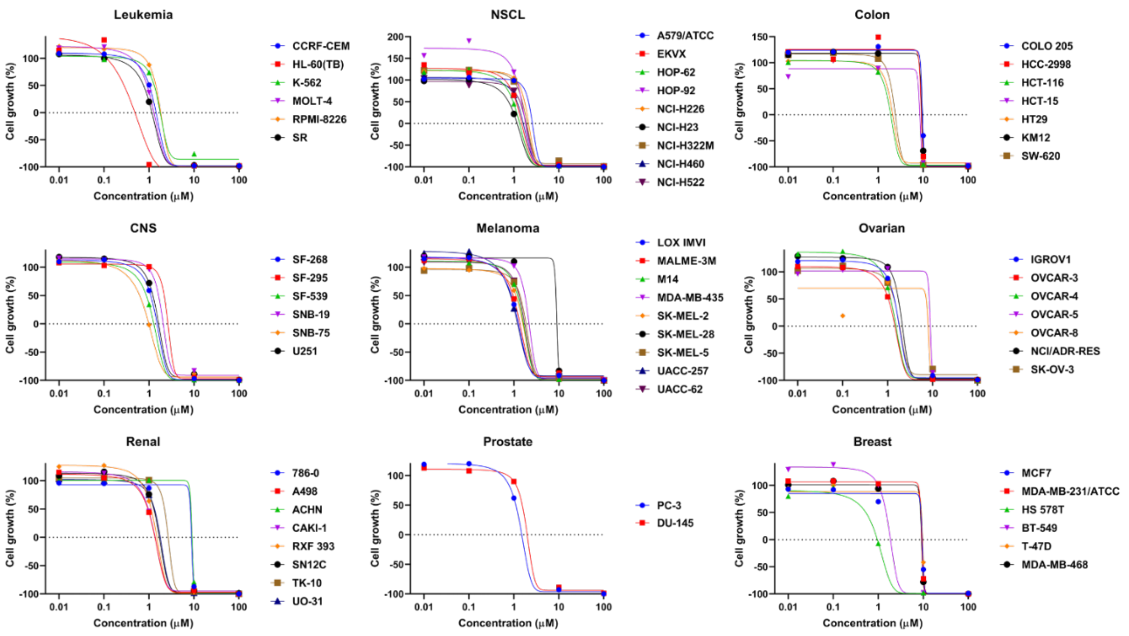

Figure S34. Dose-response curves of **2** in all the cell lines in the NCI panel.

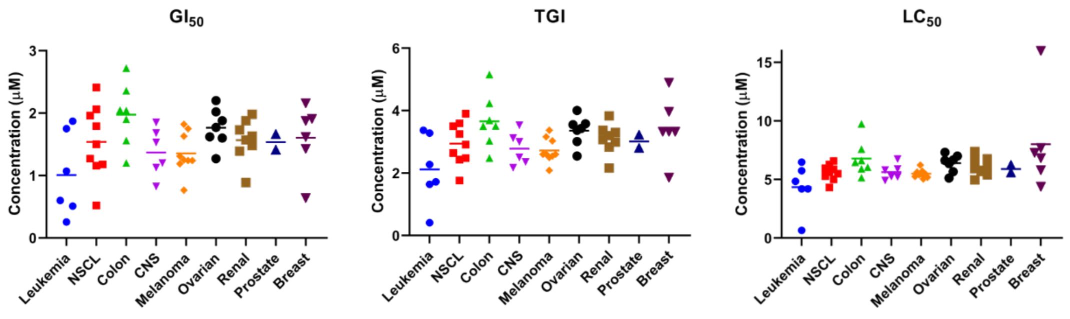

Figure S35. Mean graphs of the GI_{50}, TGI, and LC_{50} values of **2.**

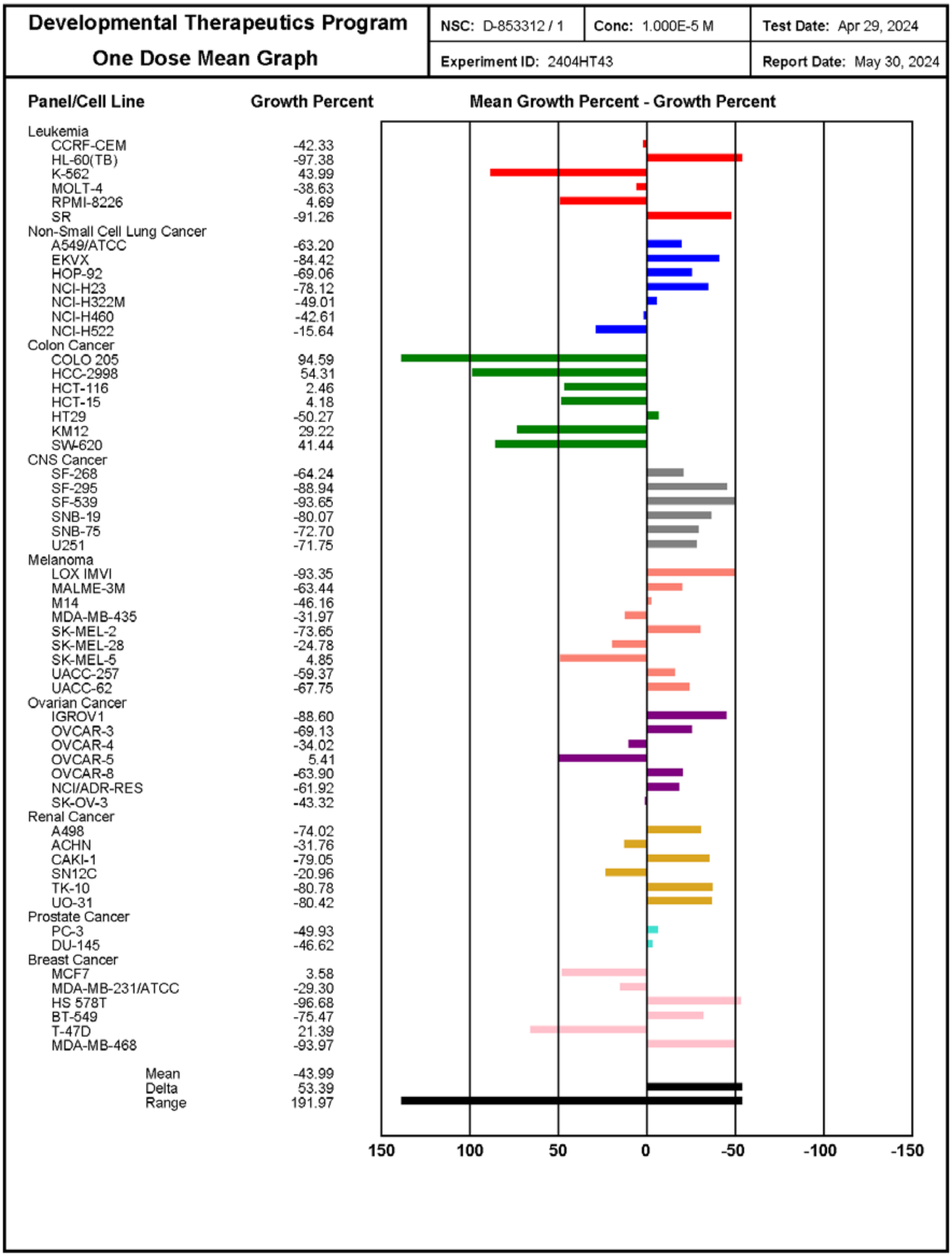

Figure S36. NCI-60 results at one dose (10 µM) of compound **3** (NSC 853312) after 48 h of treatment.

National Cancer Institute Developmental Therapeutics Program<br>In-Vitro Testing Results			
NSC : D - 853312 / 1	Experiment ID : 2407HT60	Test Type : HTS	Units : Molar
Report Date : December 4, 2024	Test Date : July 29, 2024	QNS :	MC :
COMI : C28	Stain Reagent :	SSPL : 0ZVG	

			Log10 Concentration												
	Time		Mean Optical Densities					Percent Growth							
Panel/Cell Line	Zero	Ctrl	-8.0	-7.0	-6.0	-5.0	-4.0	-8.0	-7.0	-6.0	-5.0	-4.0	GI50	TGI	LC50
Leukemia															
CCRF-CEM	0.676	4.286	4.721	4.627	4.443	0.861	0.001	112	109	104	5	-100	* 3.54E-6	* 1.12E-5	* 3.35E-5
HL-60(TB)	0.587	2.824	2.980	3.263	3.150	0.001	0.000	106	118	114	-100	-100	* 2.00E-6	* 3.42E-6	* 5.85E-6
K-562	0.506	7.390	7.538	7.880	8.286	3.105	0.003	102	107	113	38	-99	* 6.88E-6	* 1.89E-5	* 4.36E-5
MOLT-4	0.700	3.609	4.208	4.131	3.617	0.346	0.001	121	118	100	-51	-100	* 2.15E-6	* 4.62E-6	* 9.91E-6
RPMI-8226	4.400	10.261	11.401	11.118	9.229	4.175	0.003	120	115	83	-6	-100	* 2.34E-6	* 8.62E-6	* 2.95E-5
SR	0.057	0.620	0.612	0.661	0.530	0.001	0.000	98	107	84	-98	-100	* 1.54E-6	* 2.89E-6	* 5.45E-6
Non-Small Cell Lung Cancer															
A549/ATCC	0.612	4.388	4.557	4.414	4.519	0.143	0.031	104	101	103	-77	-95	* 1.98E-6	* 3.75E-6	* 7.11E-6
EKVX	2.594	7.333	8.362	8.122	7.706	0.348	0.111	122	117	108	-87	-96	* 1.98E-6	* 3.59E-6	* 6.48E-6
HOP-62	1.049	3.448	3.796	3.688	3.635	0.095	0.045	114	110	108	-91	-96	* 1.95E-6	* 3.49E-6	* 6.22E-6
HOP-92	8.768	10.268	11.436	11.538	10.945	0.851	0.480	179	187	143	-90	-94	* 2.51E-6	* 4.11E-6	* 6.72E-6
NCI-H226	6.508	13.383	14.910	14.874	14.442	0.467	0.897	122	122	115	-93	-86	* 2.06E-6	* 3.58E-6	* 6.23E-6
NCI-H23	4.317	11.185	11.646	11.411	10.565	2.090	0.012	107	103	91	-52	-100	* 1.94E-6	* 4.35E-6	* 9.75E-6
NCI-H322M	4.446	11.942	13.081	13.776	12.809	2.603	0.448	115	124	112	-41	-90	* 2.53E-6	* 5.36E-6	* 1.50E-5
NCI-H460	1.246	18.944	18.408	19.900	18.786	1.641	0.004	97	105	99	2	-100	* 3.21E-6	* 1.05E-5	* 3.26E-5
NCI-H522	3.641	9.201	9.496	8.963	8.613	3.354	0.006	105	96	89	-8	-100	* 2.53E-6	* 8.29E-6	* 2.87E-5
Colon Cancer															
COLO 205	1.187	4.714	5.540	5.442	5.670	5.007	0.117	123	121	127	108	-90	* 1.97E-5	* 3.51E-5	* 6.28E-5
HCC-2998	1.982	5.441	3.722	7.208	6.099	3.751	0.085	50	151	119	51	-96	* 1.02E-5	* 2.23E-5	* 4.88E-5
HCT-116	0.434	4.709	4.960	5.128	4.896	0.905	0.005	106	110	104	11	-99	* 3.82E-6	* 1.26E-5	* 3.59E-5
HCT-15	1.389	11.919	13.169	11.575	12.810	2.719	0.027	112	97	108	13	-98	* 4.07E-6	* 1.30E-5	* 3.68E-5
HT29	0.630	5.226	5.376	5.243	5.547	0.566	0.024	103	100	107	-10	-96	* 3.07E-6	* 8.21E-6	* 2.91E-5
KM12	0.537	3.106	3.425	3.540	3.567	2.033	0.013	112	117	118	58	-98	* 1.13E-5	* 2.36E-5	* 4.95E-5
SW-620	0.598	4.200	4.726	4.836	4.504	2.099	0.004	115	118	108	42	-99	* 7.50E-6	* 1.97E-5	* 4.47E-5
CNS Cancer															
SF-268	0.654	2.157	2.271	2.353	2.212	0.448	0.004	108	113	104	-32	-99	* 2.50E-6	* 5.85E-6	* 1.87E-5
SF-295	1.921	4.564	4.442	4.571	4.643	0.390	0.270	95	100	103	-80	-86	* 1.95E-6	* 3.66E-6	* 6.88E-6
SF-539	3.656	12.884	13.107	13.641	13.321	0.314	0.012	102	108	105	-91	-100	* 1.90E-6	* 3.42E-6	* 6.15E-6
SNB-19	1.925	4.811	5.239	5.067	4.956	0.491	0.136	115	109	105	-74	-93	* 2.02E-6	* 3.84E-6	* 7.30E-6
SNB-75	1.257	2.396	2.531	2.510	2.396	0.378	0.031	112	110	100	-70	-98	* 1.97E-6	* 3.88E-6	* 7.63E-6
U251	0.750	4.047	4.485	4.507	4.294	0.193	0.004	113	114	107	-74	-100	* 2.07E-6	* 3.90E-6	* 7.35E-6
Melanoma															
LOX IMVI	0.607	3.513	3.872	3.961	3.386	0.041	0.001	112	115	96	-93	-100	* 1.74E-6	* 3.21E-6	* 5.91E-6
MALME-3M	6.325	8.944	9.359	9.542	8.894	2.473	0.061	116	123	98	-61	-99	* 2.01E-6	* 4.14E-6	* 8.54E-6
M14	4.271	11.958	12.830	12.804	11.279	1.893	0.039	111	111	91	-56	-99	* 1.91E-6	* 4.18E-6	* 9.15E-6
MDA-MB-435	1.473	4.874	5.354	5.446	5.246	0.942	0.014	114	117	111	-36	-99	* 2.60E-6	* 5.69E-6	* 1.67E-5
SK-MEL-2	2.519	5.550	5.373	5.546	5.400	1.465	0.006	94	100	95	-42	-100	* 2.13E-6	* 4.95E-6	* 1.38E-5
SK-MEL-28	1.431	3.164	3.309	3.497	3.606	1.506	0.064	109	119	126	4	-96	* 4.19E-6	* 1.10E-5	* 3.50E-5
SK-MEL-5	3.825	11.459	11.373	12.561	12.463	2.265	0.008	99	114	113	-41	-100	* 2.58E-6	* 5.44E-6	* 1.43E-5
UACC-257	3.486	7.298	8.009	8.091	6.740	1.476	0.043	119	121	86	-58	-99	* 1.77E-6	* 3.95E-6	* 8.84E-6
UACC-62	0.739	3.049	3.348	3.126	2.960	0.367	0.005	113	103	96	-50	-99	* 2.07E-6	* 4.53E-6	* 9.95E-6
Ovarian Cancer															
IGROV1	1.018	3.394	3.856	3.893	3.332	0.383	0.033	119	121	97	-62	-97	* 1.98E-6	* 4.07E-6	* 8.37E-6
OVCAR-3	4.654	18.685	19.153	19.566	16.001	1.313	0.031	103	106	81	-72	-99	* 1.59E-6	* 3.39E-6	* 7.20E-6
OVCAR-4	5.328	9.372	10.626	10.445	9.892	4.445	0.021	131	127	113	-17	-100	* 3.06E-6	* 7.45E-6	* 2.53E-5
OVCAR-5	6.474	14.805	15.483	14.940	15.344	6.176	0.623	108	102	107	-5	-90	* 3.23E-6	* 9.09E-6	* 3.38E-5
OVCAR-8	0.673	3.725	3.827	4.068	3.872	0.449	0.014	103	111	105	-33	-98	* 2.50E-6	* 5.75E-6	* 1.82E-5
NCI/ADR-RES	3.746	10.491	11.699	11.864	11.338	1.365	0.104	118	120	113	-64	-97	* 2.27E-6	* 4.36E-6	* 8.38E-6
SK-OV-3	2.611	9.797	10.092	9.943	9.695	2.040	0.272	104	102	99	-22	-90	* 2.53E-6	* 6.58E-6	* 2.60E-5
Renal Cancer															
786-0	1.778	6.495	6.254	6.392	5.956	1.206	0.007	95	98	89	-32	-100	* 2.09E-6	* 5.42E-6	* 1.84E-5
A498	1.927	7.756	8.327	8.317	7.457	0.992	0.088	110	110	95	-48	-95	* 2.05E-6	* 4.58E-6	* 1.08E-5
ACHN	1.537	6.486	6.363	6.382	6.539	1.480	0.346	98	98	101	-4	-78	* 3.06E-6	* 9.13E-6	* 4.22E-5
CAKI-1	1.045	4.167	4.492	4.551	4.300	0.395	0.112	110	112	104	-62	-89	* 2.12E-6	* 4.23E-6	* 8.45E-6
RXF 393	1.802	3.005	3.244	3.262	3.286	0.721	0.015	120	121	124	-60	-99	* 2.52E-6	* 4.71E-6	* 8.82E-6
SN12C	0.623	2.418	2.578	2.729	2.623	0.417	0.013	109	117	111	-33	-98	* 2.66E-6	* 5.90E-6	* 1.82E-5
TK-10	6.290	16.819	17.498	17.344	17.478	3.006	0.733	106	105	106	-52	-88	* 2.26E-6	* 4.68E-6	* 9.68E-6
UO-31	1.550	5.879	5.667	5.972	5.782	0.273	0.140	95	102	98	-82	-91	* 1.84E-6	* 3.49E-6	* 6.61E-6
Prostate Cancer															
PC-3	3.978	11.351	12.109	12.584	11.125	2.442	0.175	110	117	97	-39	-96	* 2.22E-6	* 5.19E-6	* 1.58E-5
DU-145	0.636	3.759	4.107	4.022	3.717	0.295	0.029	111	109	99	-54	-96	* 2.09E-6	* 4.45E-6	* 9.48E-6
Breast Cancer															
MCF7	2.550	15.066	14.532	13.968	11.787	3.362	0.026	96	91	74	6	-99	* 2.25E-6	* 1.15E-5	* 3.43E-5
MDA-MB-231/ATCC	5.261	12.471	13.003	12.773	13.978	6.014	0.814	107	104	121	10	-84	* 4.38E-6	* 1.28E-5	* 4.32E-5
HS 578T	1.006	2.714	2.119	3.111	2.653	0.096	0.027	65	123	96	-90	-97	* 1.77E-6	* 3.28E-6	* 6.08E-6
BT-549	3.822	8.044	9.508	8.890	8.417	1.701	0.010	134	120	109	-56	-100	* 2.29E-6	* 4.60E-6	* 9.26E-6
T-47D	5.838	11.540	11.543	11.424	11.024	6.440	0.135	100	98	91	10	-98	* 3.23E-6	* 1.25E-5	* 3.63E-5
MDA-MB-468	6.310	10.749	10.644	11.015	10.668	0.818	0.025	98	106	98	-87	-100	* 1.82E-6	* 3.39E-6	* 6.31E-6

Figure S37. NCI's DTP dose-response report for compound **3**. GI_{50}, TGI and LC_{50} values (expressed as molarity).

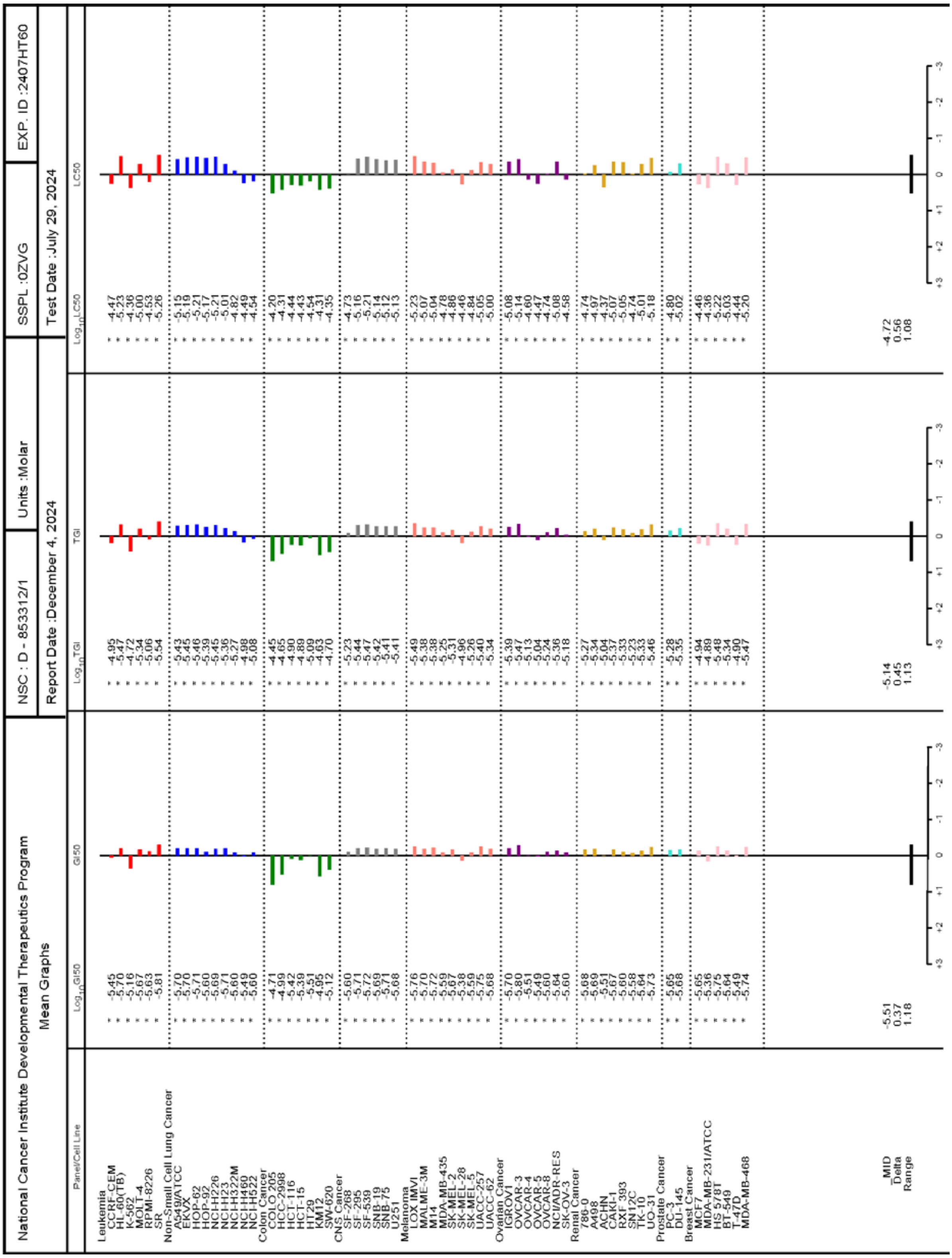

Figure S38. NCI's DTP dose-response report for compound **3**. Mean graphs of GI_{50}, TGI and LC_{50} values.

A)

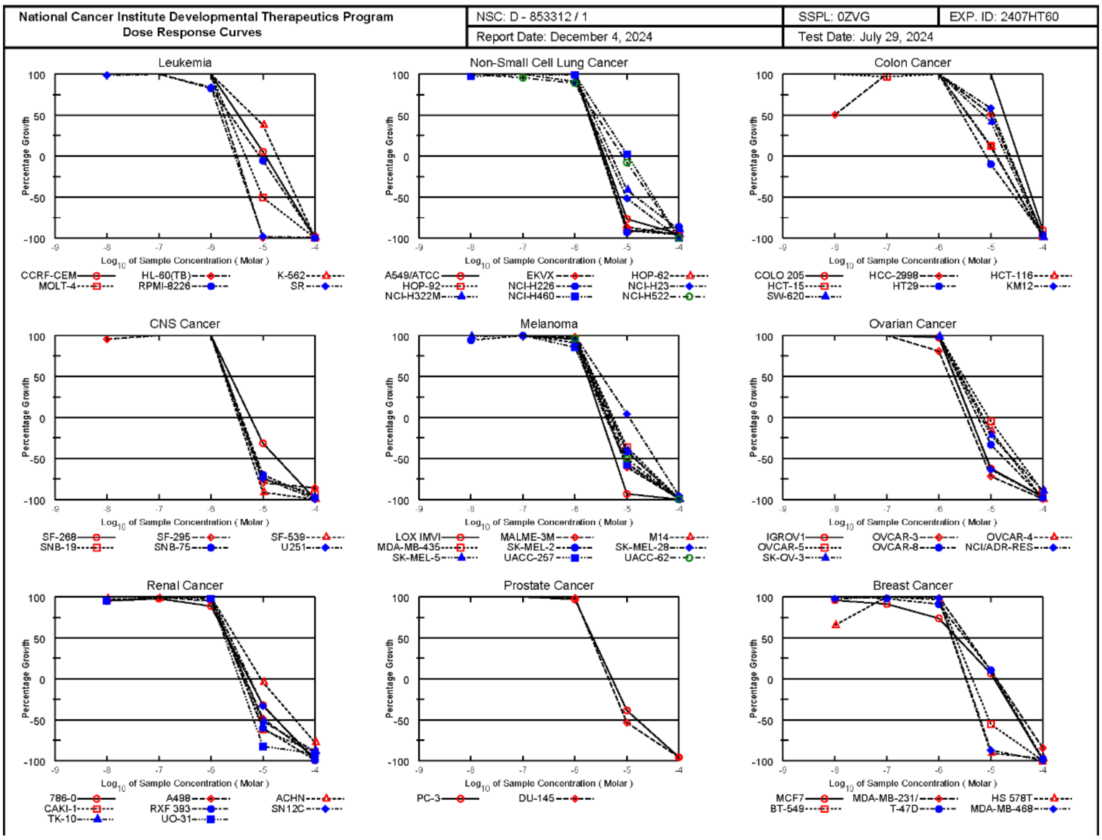

B)

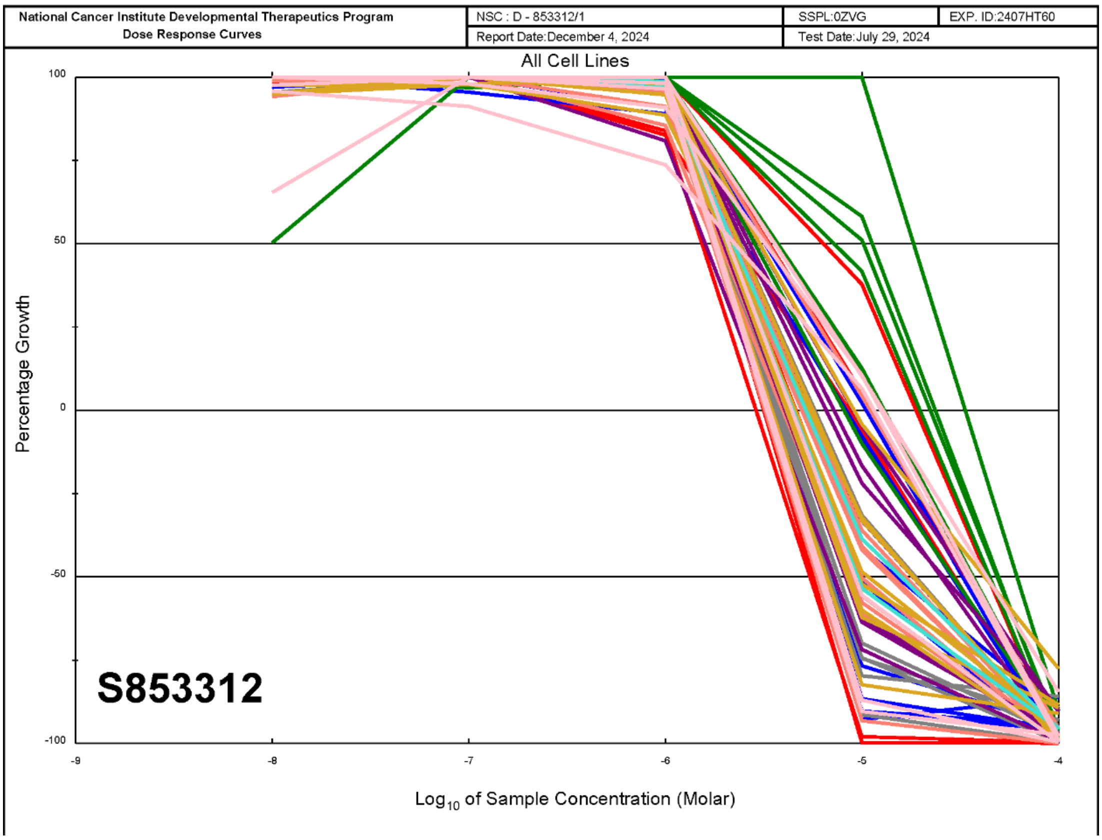

Figure S39. Dose-response curves of the NCI analysis for **3**. (**A**) Curves grouped in nine subpanels derived from different cancer types. (**B**) Curves obtained for all the cancer cell lines tested.

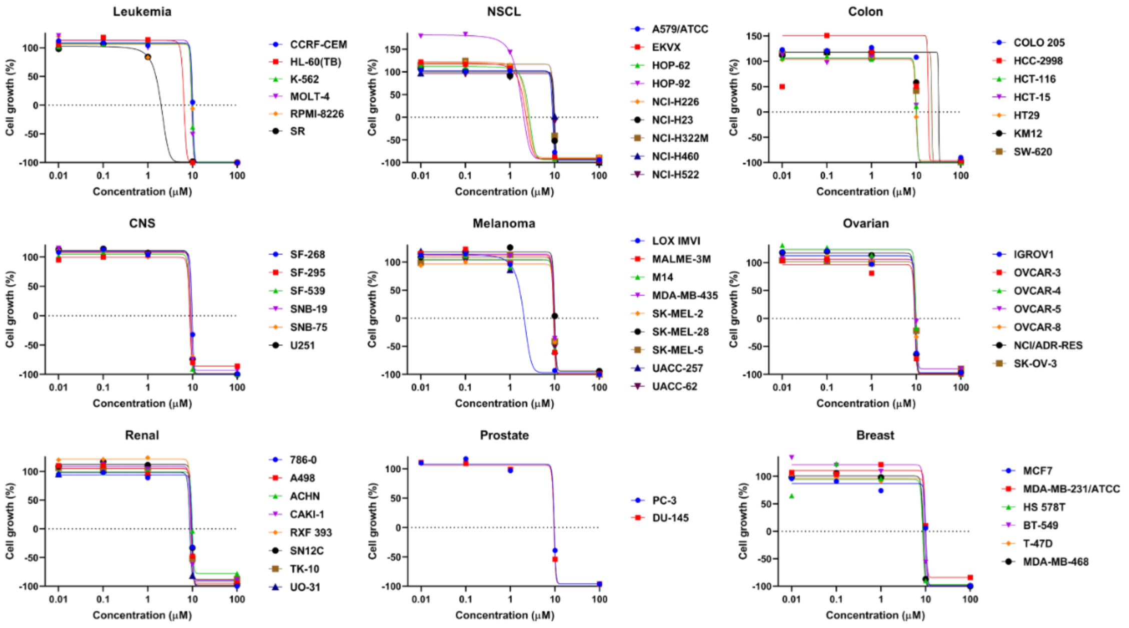

Figure S40. Dose-response curves of **3** in all the cell lines in the NCI panel.

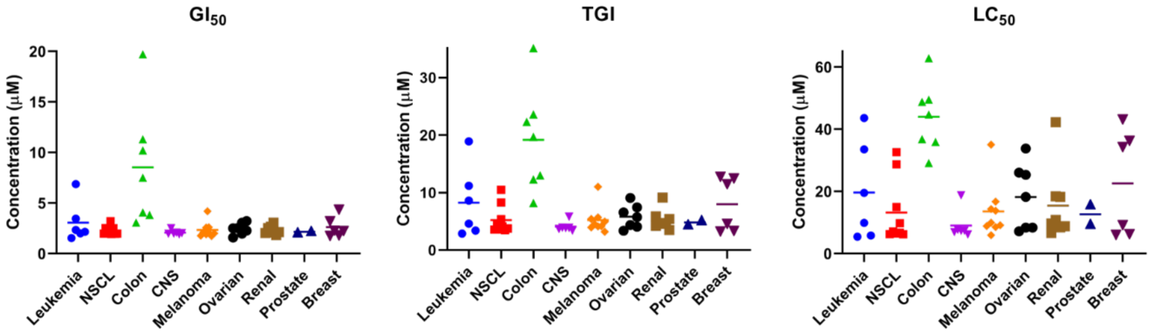

Figure S41. Mean graphs of the GI_{50}, TGI, and LC_{50} values of **3**.

Developmental Therapeutics Program	NSC: D-841524 / 1	Conc: 1.00E-5 Molar	Test Date: Jan 23, 2023
One Dose Mean Graph	Experiment ID: 2301OS90		Report Date: Feb 24, 2023

Panel/Cell Line	Growth Percent
**Leukemia**	
CCRF-CEM	-2.29
HL-60(TB)	1.60
K-562	12.95
MOLT-4	12.67
RPMI-8226	4.71
SR	4.64
**Non-Small Cell Lung Cancer**	
A549/ATCC	-32.98
EKVX	-56.64
HOP-62	-63.76
HOP-92	-53.87
NCI-H226	-13.26
NCI-H23	-37.89
NCI-H322M	2.42
NCI-H460	-30.04
NCI-H522	-55.26
**Colon Cancer**	
COLO 205	-49.57
HCC-2998	8.96
HCT-116	-36.04
HCT-15	-47.59
HT29	3.46
KM12	-20.22
SW-620	2.29
**CNS Cancer**	
SF-268	-11.02
SF-295	-49.73
SF-539	-88.88
SNB-19	-67.08
SNB-75	-90.17
U251	-56.67
**Melanoma**	
LOX IMVI	-56.27
MALME-3M	-86.43
M14	-61.30
MDA-MB-435	-33.11
SK-MEL-2	-30.61
SK-MEL-28	-33.39
SK-MEL-5	-88.91
UACC-257	-44.00
UACC-62	-75.53
**Ovarian Cancer**	
IGROV1	-61.69
OVCAR-3	-51.76
OVCAR-4	6.13
OVCAR-5	-58.36
OVCAR-8	-51.78
NCI/ADR-RES	17.53
SK-OV-3	1.87
**Renal Cancer**	
786-0	-29.97
A498	-60.19
ACHN	-29.58
CAKI-1	-56.13
RXF 393	-51.55
SN12C	-19.35
TK-10	-33.55
UO-31	-40.57
**Prostate Cancer**	
PC-3	3.20
DU-145	-59.43
**Breast Cancer**	
MCF7	8.21
MDA-MB-231/ATCC	-67.34
HS 578T	-20.10
BT-549	-74.66
T-47D	36.82
MDA-MB-468	-33.24
Mean	-34.07
Delta	56.10
Range	126.99

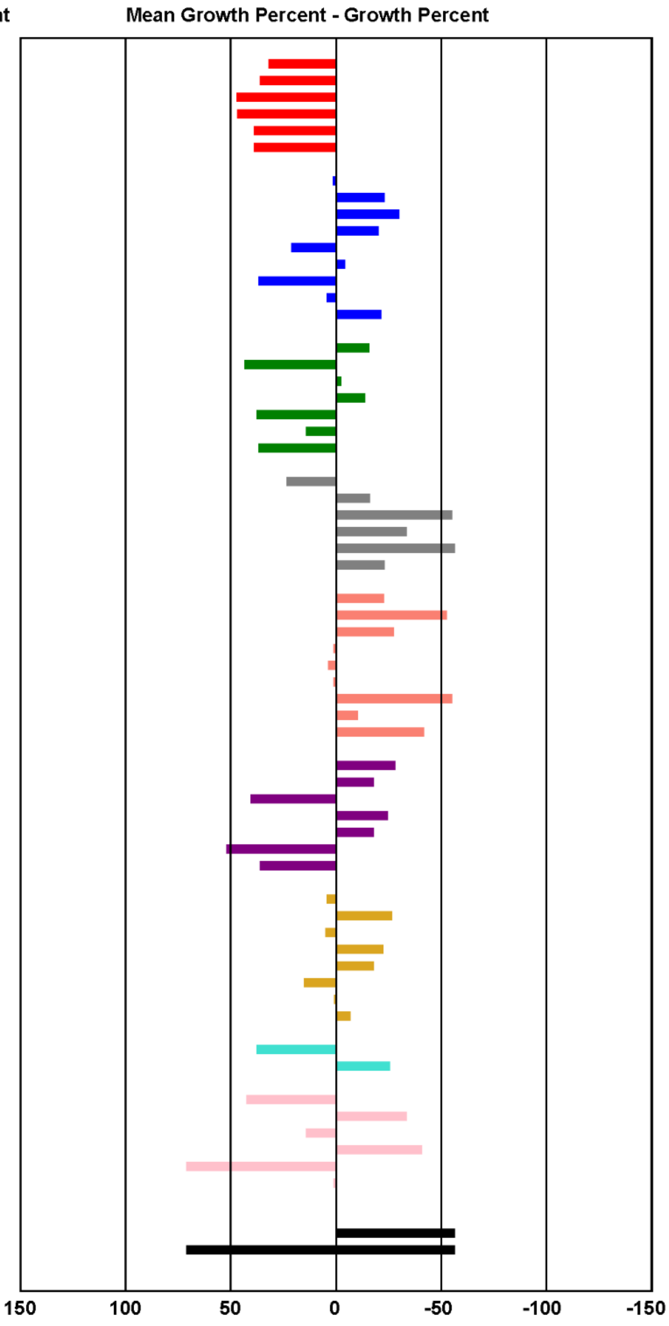

Figure S42. NCI-60 results at one dose (10 μM) of compound **4** (NSC 841524) after 48 h of treatment.

National Cancer Institute Developmental Therapeutics Program
In-Vitro Testing Results

NSC : D - 841524 / 1	Experiment ID : 2306NS34	Test Type : 08	Units : Molar
Report Date : July 19, 2023	Test Date : June 12, 2023	QNS :	MC :
COMI : C3	Stain Reagent : SRB Dual-Pass Related	SSPL : 0ZVG	

			Log10 Concentration												
	Time		Mean Optical Densities					Percent Growth							
Panel/Cell Line	Zero	Ctrl	-8.0	-7.0	-6.0	-5.0	-4.0	-8.0	-7.0	-6.0	-5.0	-4.0	GI50	TGI	LC50
Leukemia															
CCRF-CEM	0.487	2.424	2.510	2.597	1.918	0.511	0.450	104	109	74	1	-8	2.13E-6	1.38E-5	> 1.00E-4
HL-60(TB)	0.593	2.716	2.297	2.227	1.114	0.428	0.461	80	77	25	-28	-22	3.27E-7	2.94E-6	> 1.00E-4
K-562	0.199	1.802	1.795	1.773	1.496	0.244	0.124	100	98	81	3	-38	2.49E-6	1.17E-5	> 1.00E-4
MOLT-4	0.568	2.474	2.461	1.836	1.761	0.569	0.523	99	67	63	0	-8	1.59E-6	1.01E-5	> 1.00E-4
RPMI-8226	0.778	2.411	2.491	2.521	2.132	0.714	0.832	105	107	83	-8	3	2.30E-6	.	> 1.00E-4
SR	0.409	2.178	2.049	1.995	1.803	0.472	0.420	93	90	79	4	1	2.41E-6	> 1.00E-4	> 1.00E-4
Non-Small Cell Lung Cancer															
A549/ATCC	0.679	2.745	2.666	2.702	2.475	0.419	0.196	96	98	87	-38	-71	1.97E-6	4.95E-6	2.27E-5
EKVX	0.862	2.462	2.339	2.405	2.100	0.316	0.650	92	96	77	-63	-25	1.56E-6	3.54E-6	.
HOP-62	0.634	2.098	2.015	2.035	1.963	0.134	0.257	94	96	91	-79	-59	1.74E-6	3.43E-6	6.75E-6
HOP-92	1.227	1.748	1.653	1.716	1.634	0.365	0.391	82	94	78	-70	-68	1.55E-6	3.36E-6	7.30E-6
NCI-H226	0.824	1.289	1.234	1.345	1.199	0.392	0.402	88	112	81	-52	-51	1.70E-6	4.04E-6	9.59E-6
NCI-H23	0.585	1.815	1.850	1.817	1.027	0.239	0.225	103	100	36	-59	-62	6.04E-7	2.39E-6	8.00E-6
NCI-H322M	0.625	1.816	1.820	1.828	1.490	0.405	0.217	100	101	73	-35	-65	1.62E-6	4.71E-6	3.09E-5
NCI-H460	0.448	2.986	3.002	2.988	2.299	0.185	0.180	101	100	73	-59	-60	1.49E-6	3.58E-6	8.59E-6
NCI-H522	1.154	2.889	2.799	2.891	2.714	0.371	0.752	95	100	90	-68	-35	1.79E-6	3.71E-6	.
Colon Cancer															
COLO 205	0.446	1.980	1.959	2.005	1.859	0.057	0.069	99	102	92	-87	-85	1.72E-6	3.26E-6	6.20E-6
HCC-2998	0.528	2.096	1.903	2.036	2.024	0.360	0.053	88	96	95	-32	-90	2.27E-6	5.61E-6	2.05E-5
HCT-116	0.211	1.732	1.811	1.791	1.283	0.047	0.099	105	104	70	-78	-53	1.37E-6	2.99E-6	6.50E-6
HCT-15	0.353	2.009	1.904	1.996	1.658	0.123	0.208	94	99	79	-65	-41	1.58E-6	3.53E-6	.
HT29	0.312	1.931	1.964	2.060	1.895	0.267	0.220	102	108	98	-15	-30	2.66E-6	7.42E-6	> 1.00E-4
KM12	1.046	3.221	3.191	3.248	2.926	0.681	0.234	99	101	86	-35	-78	2.00E-6	5.15E-6	2.25E-5
SW-620	0.379	2.182	2.185	2.209	1.904	0.187	0.088	100	101	85	-51	-77	1.80E-6	4.21E-6	9.87E-6
CNS Cancer															
SF-268	1.305	2.702	2.628	2.684	2.557	0.832	0.862	95	99	90	-36	-34	2.06E-6	5.15E-6	> 1.00E-4
SF-295	1.009	2.583	2.519	2.574	2.594	0.248	0.094	96	99	101	-75	-91	1.94E-6	3.73E-6	7.17E-6
SF-539	0.824	2.412	2.257	2.328	2.022	0.078	0.071	90	95	75	-91	-91	1.42E-6	2.85E-6	5.70E-6
SNB-19	0.590	1.917	1.842	1.869	1.824	0.164	0.128	94	96	93	-72	-78	1.82E-6	3.65E-6	7.33E-6
U251	0.403	1.959	1.862	1.930	1.697	0.193	0.173	94	98	83	-52	-57	1.76E-6	4.12E-6	9.65E-6
Melanoma															
LOX IMVI	0.387	2.439	2.219	2.278	1.058	0.051	0.040	89	92	33	-87	-90	5.12E-7	1.88E-6	4.91E-6
MALME-3M	0.448	1.021	0.966	1.029	0.851	0.139	0.128	90	101	70	-69	-71	1.40E-6	3.19E-6	7.30E-6
M14	0.486	1.927	1.843	1.866	1.378	0.162	0.094	94	96	62	-67	-81	1.24E-6	3.03E-6	7.41E-6
MDA-MB-435	0.634	2.429	2.364	2.397	2.105	0.221	0.030	96	98	82	-65	-95	1.65E-6	3.61E-6	7.89E-6
SK-MEL-2	1.299	3.004	2.994	3.010	2.849	0.499	0.645	99	100	91	-62	-50	1.85E-6	3.94E-6	8.39E-6
SK-MEL-28	0.774	2.121	2.169	2.170	1.926	0.286	0.046	104	104	86	-63	-94	1.73E-6	3.76E-6	8.16E-6
SK-MEL-5	1.016	3.168	3.031	3.080	2.710	0.085	0.018	94	96	79	-92	-98	1.47E-6	2.90E-6	5.69E-6
UACC-257	1.253	2.654	2.628	2.755	2.385	0.689	0.393	98	107	81	-45	-69	1.76E-6	4.39E-6	1.62E-5
UACC-62	0.980	2.740	2.552	2.524	2.270	0.068	0.092	89	88	73	-93	-91	1.38E-6	2.76E-6	5.51E-6
Ovarian Cancer															
IGROV1	0.466	1.901	1.887	2.036	1.418	0.217	0.356	99	109	66	-53	-24	1.37E-6	3.58E-6	.
OVCAR-3	0.323	0.936	1.030	1.000	0.608	0.075	0.201	115	110	47	-77	-38	8.83E-7	2.38E-6	.
OVCAR-4	1.459	2.571	2.518	2.576	2.214	1.054	1.089	95	100	68	-28	-25	1.54E-6	5.12E-6	> 1.00E-4
OVCAR-5	0.679	1.593	1.616	1.601	1.567	0.249	0.174	103	101	97	-63	-74	1.97E-6	4.03E-6	8.25E-6
OVCAR-8	0.434	2.097	2.129	2.163	1.887	0.250	0.295	102	104	87	-42	-32	1.94E-6	4.71E-6	> 1.00E-4
NCI/ADR-RES	0.952	2.788	2.751	2.789	2.558	0.523	0.696	98	100	87	-45	-27	1.92E-6	4.57E-6	> 1.00E-4
SK-OV-3	0.760	1.841	1.877	1.824	2.397	0.621	0.294	103	98	152	-18	-61	3.96E-6	7.80E-6	5.44E-5
Renal Cancer															
786-0	0.643	2.539	2.362	2.565	2.696	0.449	0.641	91	101	108	-30	0	2.64E-6	6.05E-6	> 1.00E-4
A498	1.630	2.141	2.062	2.106	2.058	0.578	0.039	85	93	84	-65	-98	1.69E-6	3.67E-6	7.98E-6
ACHN	0.463	1.913	1.936	2.080	2.028	0.287	0.166	102	112	108	-38	-64	2.49E-6	5.48E-6	2.85E-5
CAKI-1	0.914	2.050	1.932	2.018	2.051	0.398	0.379	90	97	100	-57	-59	2.09E-6	4.36E-6	9.09E-6
RXF 393	0.954	1.643	1.589	1.628	1.538	0.404	0.310	92	98	85	-58	-68	1.75E-6	3.93E-6	8.84E-6
SN12C	0.689	2.382	2.116	2.191	2.006	0.427	0.174	84	89	78	-38	-75	1.74E-6	4.69E-6	2.11E-5
TK-10	1.392	2.855	2.724	2.816	2.846	0.455	0.718	91	97	99	-67	-48	1.98E-6	3.94E-6	.
UO-31	0.594	1.664	1.433	1.475	1.132	0.113	0.145	78	82	50	-81	-76	1.00E-6	2.42E-6	5.81E-6
Prostate Cancer															
PC-3	0.560	1.808	1.759	1.807	1.452	0.416	0.342	96	100	71	-26	-39	1.66E-6	5.44E-6	> 1.00E-4
DU-145	0.438	1.500	1.482	1.543	1.352	0.081	0.044	98	104	86	-82	-90	1.64E-6	3.26E-6	6.48E-6
Breast Cancer															
MCF7	0.452	2.449	2.264	2.374	1.887	0.401	0.292	91	96	72	-11	-35	1.83E-6	7.32E-6	> 1.00E-4
MDA-MB-231/ATCC	0.570	1.156	1.164	1.176	1.064	0.211	0.330	101	103	84	-63	-42	1.71E-6	3.73E-6	.
HS 578T	1.198	2.251	2.145	2.158	1.884	0.729	0.907	90	91	65	-39	-24	1.40E-6	4.21E-6	> 1.00E-4
BT-549	1.095	1.953	1.878	1.954	1.964	0.202	0.440	91	100	101	-82	-60	1.91E-6	3.58E-6	6.72E-6
T-47D	0.756	1.909	1.802	1.822	1.549	0.798	0.579	91	92	69	4	-23	1.94E-6	1.36E-5	> 1.00E-4
MDA-MB-468	0.858	1.207	1.185	1.219	1.108	0.424	0.346	94	104	72	-51	-60	1.51E-6	3.86E-6	9.88E-6

Figure S43. NCI's DTP dose-response report for compound **4**. GI_{50}, TGI and LC_{50} values (expressed as molarity).

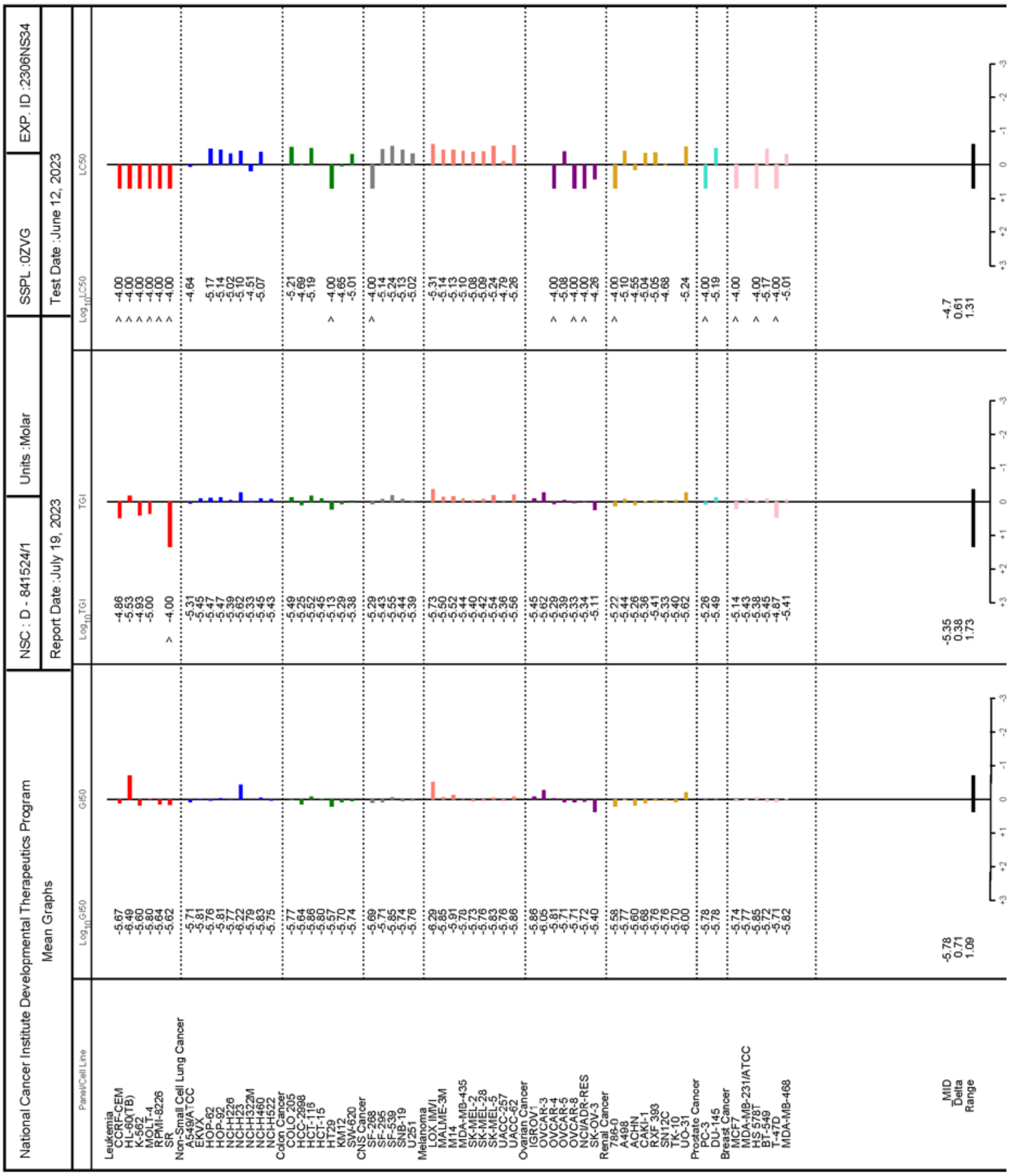

Figure S44. NCI's DTP dose-response report for compound **4**. Mean graphs of GI_{50}, TGI and LC_{50} values.

A)

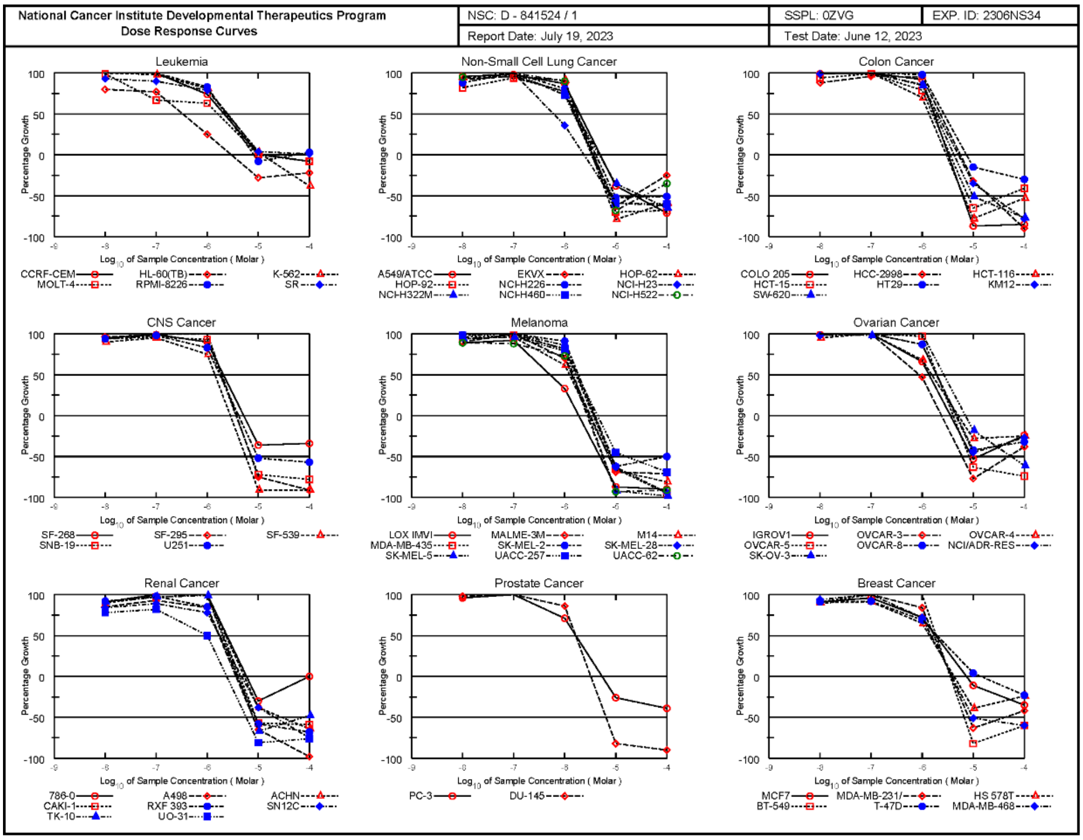

B)

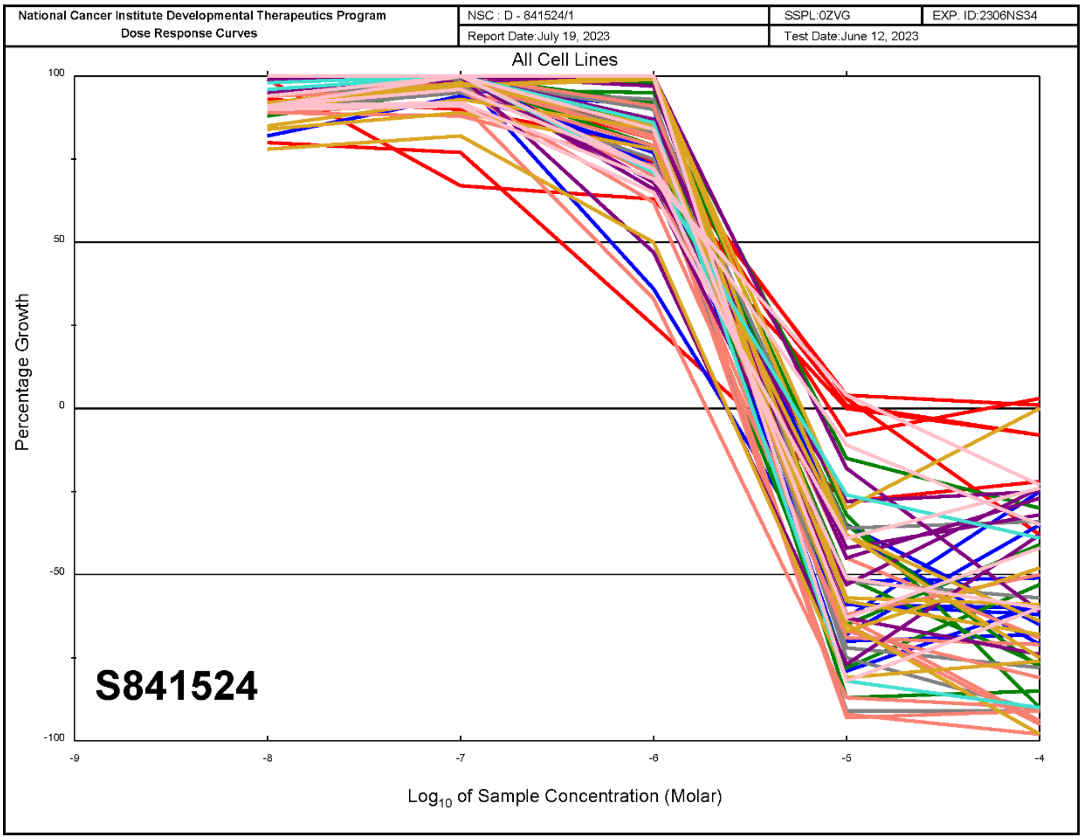

Figure S45. Dose-response curves of the NCI analysis for **4**. (**A**) Curves grouped in nine subpanels derived from different cancer types. (**B**) Curves obtained for all the cancer cell lines tested.

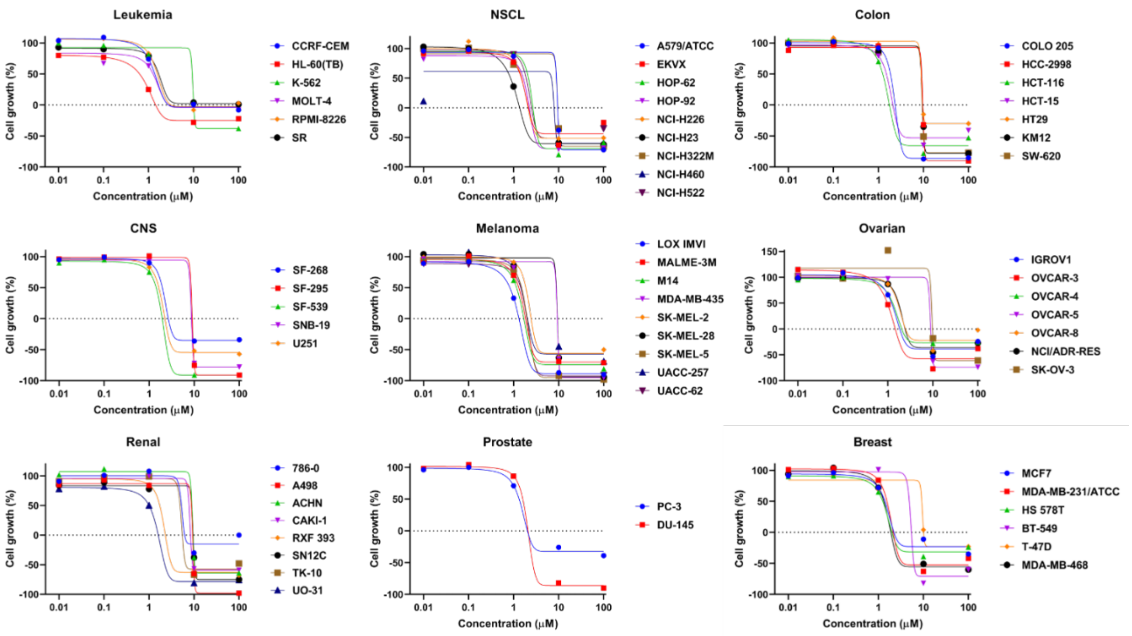

Figure S46. Dose-response curves of **4** in all the cell lines in the NCI panel.

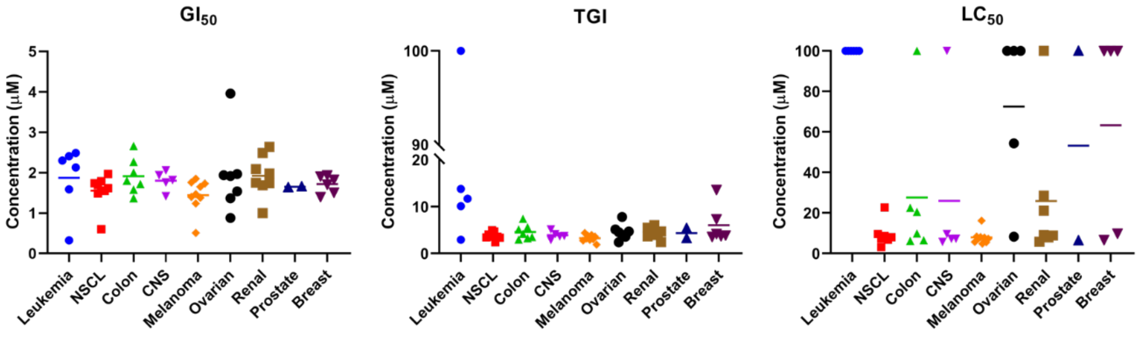

Figure S47. Mean graphs of the GI_{50}, TGI, and LC_{50} values of **4**.

Developmental Therapeutics Program One Dose Mean Graph	NSC: D-841525 / 1	Conc: 1.00E-5 Molar	Test Date: Jan 23, 2023
	Experiment ID: 2301OS90		Report Date: Feb 24, 2023

Panel/Cell Line	Growth Percent
**Leukemia**	
CCRF-CEM	0.45
HL-60(TB)	2.86
K-562	14.15
MOLT-4	13.84
RPMI-8226	2.74
SR	5.51
**Non-Small Cell Lung Cancer**	
A549/ATCC	-53.97
EKVX	-62.41
HOP-62	-65.22
HOP-92	-60.35
NCI-H226	-24.62
NCI-H23	-39.10
NCI-H322M	-2.90
NCI-H460	-26.26
NCI-H522	-47.34
**Colon Cancer**	
COLO 205	-49.91
HCC-2998	-39.76
HCT-116	-38.07
HCT-15	-7.18
HT29	4.97
KM12	3.78
SW-620	1.46
**CNS Cancer**	
SF-268	-9.35
SF-295	-61.06
SF-539	-88.80
SNB-19	-71.16
SNB-75	-75.91
U251	-51.35
**Melanoma**	
LOX IMVI	-72.28
MALME-3M	-85.95
M14	-57.75
MDA-MB-435	-21.68
SK-MEL-2	-36.12
SK-MEL-28	-32.28
SK-MEL-5	-87.27
UACC-257	-38.24
UACC-62	-74.91
**Ovarian Cancer**	
IGROV1	-56.43
OVCAR-3	-59.10
OVCAR-4	7.30
OVCAR-5	-70.32
OVCAR-8	-70.80
NCI/ADR-RES	-31.76
SK-OV-3	11.12
**Renal Cancer**	
786-0	-18.62
A498	-41.62
ACHN	-32.59
CAKI-1	-62.33
RXF 393	-64.74
SN12C	-24.00
TK-10	-34.95
UO-31	-36.90
**Prostate Cancer**	
PC-3	4.32
DU-145	-60.11
**Breast Cancer**	
MCF7	6.65
MDA-MB-231/ATCC	-74.77
HS 578T	-15.87
BT-549	-66.78
T-47D	31.93
MDA-MB-468	-54.87
Mean	-35.78
Delta	53.02
Range	120.73

Mean Growth Percent - Growth Percent

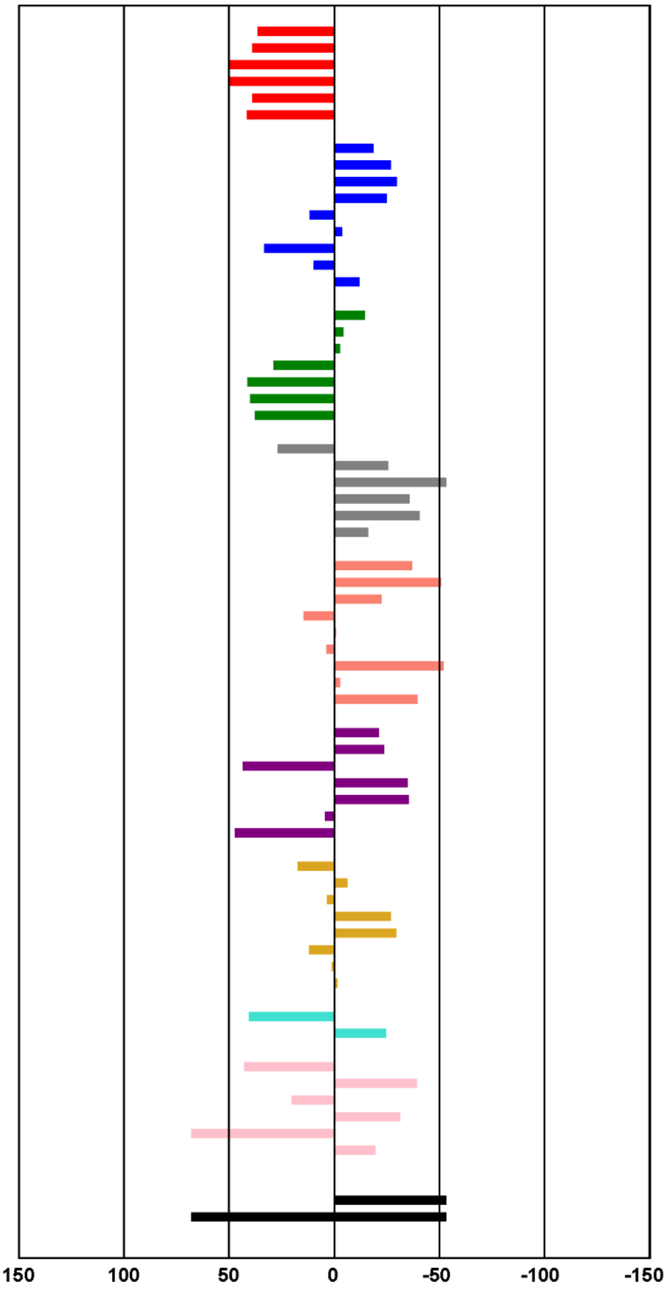

Figure S48. NCI-60 results at one dose (10 μM) of compound **5** (NSC 841525) after 48 h of treatment.

National Cancer Institute Developmental Therapeutics Program
In-Vitro Testing Results

NSC : D - 841525 / 1	Experiment ID : 2306NS34	Test Type : 08	Units : Molar
Report Date : July 19, 2023	Test Date : June 12, 2023	QNS :	MC :
COMI : C4	Stain Reagent : SRB Dual-Pass Related	SSPL : 0ZVG	

			Log10 Concentration												
	Time		Mean Optical Densities					Percent Growth							
Panel/Cell Line	Zero	Ctrl	-8.0	-7.0	-6.0	-5.0	-4.0	-8.0	-7.0	-6.0	-5.0	-4.0	GI50	TGI	LC50
**Leukemia**															
CCRF-CEM	0.487	2.622	2.537	2.400	2.074	0.498	0.481	96	90	74	1	-1	2.14E-6	1.97E-5	> 1.00E-4
HL-60(TB)	0.593	2.915	2.863	2.756	2.098	0.632	0.738	98	93	65	2	6	1.72E-6	> 1.00E-4	> 1.00E-4
K-562	0.199	1.968	2.060	2.069	1.696	0.393	0.353	105	106	85	11	9	2.95E-6	> 1.00E-4	> 1.00E-4
MOLT-4	0.568	2.816	2.775	2.590	2.111	0.744	0.722	98	90	69	8	7	2.02E-6	> 1.00E-4	> 1.00E-4
RPMI-8226	0.778	2.667	2.626	2.545	2.370	0.889	0.870	98	94	84	6	5	2.73E-6	> 1.00E-4	> 1.00E-4
SR	0.409	2.196	2.171	2.183	2.138	0.604	0.630	99	99	97	11	12	3.51E-6	> 1.00E-4	> 1.00E-4
**Non-Small Cell Lung Cancer**															
A549/ATCC	0.679	2.826	2.700	2.778	2.522	0.364	0.280	94	98	86	-46	-59	1.87E-6	4.46E-6	1.96E-5
EKVX	0.862	2.507	2.431	2.441	2.151	0.256	0.655	95	96	78	-70	-24	1.55E-6	3.37E-6	.
HOP-62	0.634	2.321	2.378	2.207	2.287	0.179	0.442	103	93	98	-72	-30	1.92E-6	3.78E-6	.
HOP-92	1.227	1.943	1.835	1.829	1.827	0.421	0.924	85	84	84	-66	-25	1.68E-6	3.63E-6	.
NCI-H226	0.824	1.336	1.297	1.350	1.277	0.569	0.462	92	103	88	-31	-44	2.10E-6	5.50E-6	> 1.00E-4
NCI-H23	0.585	1.896	1.862	1.827	1.491	0.241	0.279	97	95	69	-59	-52	1.41E-6	3.47E-6	8.52E-6
NCI-H322M	0.625	2.090	2.236	2.217	1.967	0.624	0.274	110	109	92	0	-56	2.84E-6	9.94E-6	7.74E-5
NCI-H460	0.448	3.115	3.142	3.102	2.780	0.237	0.201	101	100	87	-47	-55	1.90E-6	4.46E-6	2.22E-5
NCI-H522	1.154	2.993	2.881	2.933	2.833	0.452	0.671	94	97	91	-61	-42	1.87E-6	3.98E-6	.
**Colon Cancer**															
COLO 205	0.446	1.938	1.983	2.010	2.096	0.407	0.119	103	105	111	-9	-73	3.22E-6	8.45E-6	4.34E-5
HCC-2998	0.528	2.181	2.146	2.041	2.076	0.586	0.055	98	92	94	4	-90	3.05E-6	1.09E-5	3.76E-5
HCT-116	0.211	1.997	1.965	1.689	1.466	0.058	0.076	98	83	70	-73	-64	1.39E-6	3.11E-6	6.96E-6
HCT-15	0.353	2.102	1.981	1.936	1.700	0.121	0.207	93	91	77	-66	-42	1.55E-6	3.46E-6	.
HT29	0.312	2.217	2.168	2.167	2.226	0.425	0.320	97	97	100	6	0	3.42E-6	> 1.00E-4	> 1.00E-4
KM12	1.046	3.295	3.314	3.322	3.125	1.190	0.424	101	101	92	6	-59	3.11E-6	1.25E-5	7.18E-5
SW-620	0.379	2.284	2.312	2.361	2.303	0.230	0.086	101	104	101	-39	-77	2.31E-6	5.24E-6	1.90E-5
**CNS Cancer**															
SF-268	1.305	2.824	2.793	2.812	2.789	1.140	0.995	98	99	98	-13	-24	2.70E-6	7.68E-6	> 1.00E-4
SF-295	1.009	2.677	2.624	2.577	2.709	0.288	0.094	97	94	102	-71	-91	1.99E-6	3.87E-6	7.52E-6
SF-539	0.824	2.452	2.435	2.512	2.333	0.158	0.095	99	104	93	-81	-89	1.76E-6	3.42E-6	6.64E-6
SNB-19	0.590	2.059	1.981	1.959	1.950	0.270	0.115	95	93	93	-54	-81	1.95E-6	4.27E-6	9.35E-6
U251	0.403	2.046	1.995	1.995	1.812	0.197	0.285	97	97	86	-51	-29	1.82E-6	4.23E-6	.
**Melanoma**															
LOX IMVI	0.387	2.588	2.419	2.298	1.741	0.040	0.045	92	87	62	-90	-89	1.19E-6	2.55E-6	5.47E-6
MALME-3M	0.448	1.043	1.032	1.066	0.941	0.178	0.083	98	104	83	-60	-81	1.70E-6	3.79E-6	8.46E-6
M14	0.486	1.905	1.863	1.806	1.524	0.208	0.051	97	93	73	-57	-90	1.50E-6	3.64E-6	8.81E-6
MDA-MB-435	0.634	2.691	2.617	2.572	2.451	0.369	0.103	96	94	88	-42	-84	1.97E-6	4.77E-6	1.56E-5
SK-MEL-2	1.299	3.111	3.103	3.070	2.967	0.610	0.872	100	98	92	-53	-33	1.95E-6	4.31E-6	.
SK-MEL-28	0.774	2.222	2.164	2.123	2.000	0.418	0.052	96	93	85	-46	-93	1.84E-6	4.44E-6	1.22E-5
SK-MEL-5	1.016	3.230	3.204	3.209	2.987	0.109	0.113	99	99	89	-89	-89	1.66E-6	3.16E-6	6.02E-6
UACC-257	1.253	2.755	2.721	2.658	2.477	0.816	0.604	98	94	82	-35	-52	1.86E-6	5.01E-6	7.83E-5
UACC-62	0.980	2.814	2.633	2.654	2.546	0.214	0.094	90	91	85	-78	-90	1.65E-6	3.33E-6	6.73E-6
**Ovarian Cancer**															
IGROV1	0.466	2.011	2.054	2.280	1.846	0.326	0.352	103	117	89	-30	-24	2.13E-6	5.60E-6	> 1.00E-4
OVCAR-3	0.323	1.053	1.074	1.053	0.781	0.083	0.204	103	100	63	-74	-37	1.24E-6	2.87E-6	.
OVCAR-4	1.459	2.767	2.774	2.742	2.427	1.353	1.292	100	98	74	-7	-11	1.97E-6	8.14E-6	> 1.00E-4
OVCAR-5	0.679	1.589	1.571	1.539	1.590	0.280	0.181	98	94	100	-59	-73	2.07E-6	4.26E-6	8.80E-6
OVCAR-8	0.434	2.165	2.182	2.196	2.039	0.243	0.381	101	102	93	-44	-12	2.05E-6	4.77E-6	> 1.00E-4
NCI/ADR-RES	0.952	2.871	2.834	2.891	2.689	0.532	0.773	98	101	91	-44	-19	2.00E-6	4.70E-6	> 1.00E-4
SK-OV-3	0.760	1.961	2.031	1.889	2.391	0.819	0.554	106	94	136	5	-27	4.52E-6	1.42E-5	> 1.00E-4
**Renal Cancer**															
786-0	0.643	2.592	2.446	2.450	2.547	0.463	0.606	93	93	98	-28	-6	2.40E-6	5.99E-6	> 1.00E-4
A498	1.630	2.249	2.284	2.232	2.241	0.883	0.091	106	97	99	-46	-94	2.17E-6	4.81E-6	1.22E-5
ACHN	0.463	2.090	2.131	2.128	2.097	0.363	0.341	103	102	100	-22	-26	2.59E-6	6.64E-6	> 1.00E-4
CAKI-1	0.914	2.245	2.197	2.244	2.339	0.367	0.688	96	100	107	-60	-25	2.20E-6	4.38E-6	.
RXF 393	0.954	1.739	1.713	1.717	1.623	0.599	0.401	97	97	85	-37	-58	1.94E-6	4.97E-6	4.13E-5
SN12C	0.689	2.424	2.411	2.414	2.338	0.478	0.275	99	99	95	-31	-60	2.28E-6	5.70E-6	4.55E-5
TK-10	1.392	2.959	2.899	2.517	2.941	0.813	1.151	96	72	99	-42	-17	2.23E-6	5.05E-6	> 1.00E-4
UO-31	0.594	1.766	1.627	1.890	1.592	0.295	0.417	88	111	85	-50	-30	1.82E-6	4.25E-6	.
**Prostate Cancer**															
PC-3	0.560	2.127	1.972	1.865	1.597	0.545	0.501	90	83	66	-3	-11	1.72E-6	9.12E-6	> 1.00E-4
DU-145	0.438	1.630	1.682	1.473	1.577	0.179	0.121	104	87	96	-59	-72	1.97E-6	4.14E-6	8.72E-6
**Breast Cancer**															
MCF7	0.452	2.454	2.333	2.366	2.014	0.465	0.345	94	96	78	1	-24	2.30E-6	1.06E-5	> 1.00E-4
MDA-MB-231/ATCC	0.570	1.229	1.251	1.281	1.234	0.169	0.407	103	108	101	-70	-29	1.98E-6	3.88E-6	.
HS 578T	1.198	2.380	2.342	2.313	2.111	0.996	1.044	97	94	77	-17	-13	1.95E-6	6.61E-6	> 1.00E-4
BT-549	1.095	2.044	1.914	2.001	1.850	0.306	0.605	86	96	80	-72	-45	1.57E-6	3.35E-6	.
T-47D	0.756	1.970	1.901	1.718	1.611	1.032	0.859	94	79	70	23	8	2.68E-6	> 1.00E-4	> 1.00E-4
MDA-MB-468	0.858	1.310	1.272	1.224	1.206	0.357	0.338	92	81	77	-58	-61	1.58E-6	3.70E-6	8.66E-6

Figure S49. NCI's DTP dose-response report for compound **5**. GI_{50}, TGI and LC_{50} values (expressed as molarity).

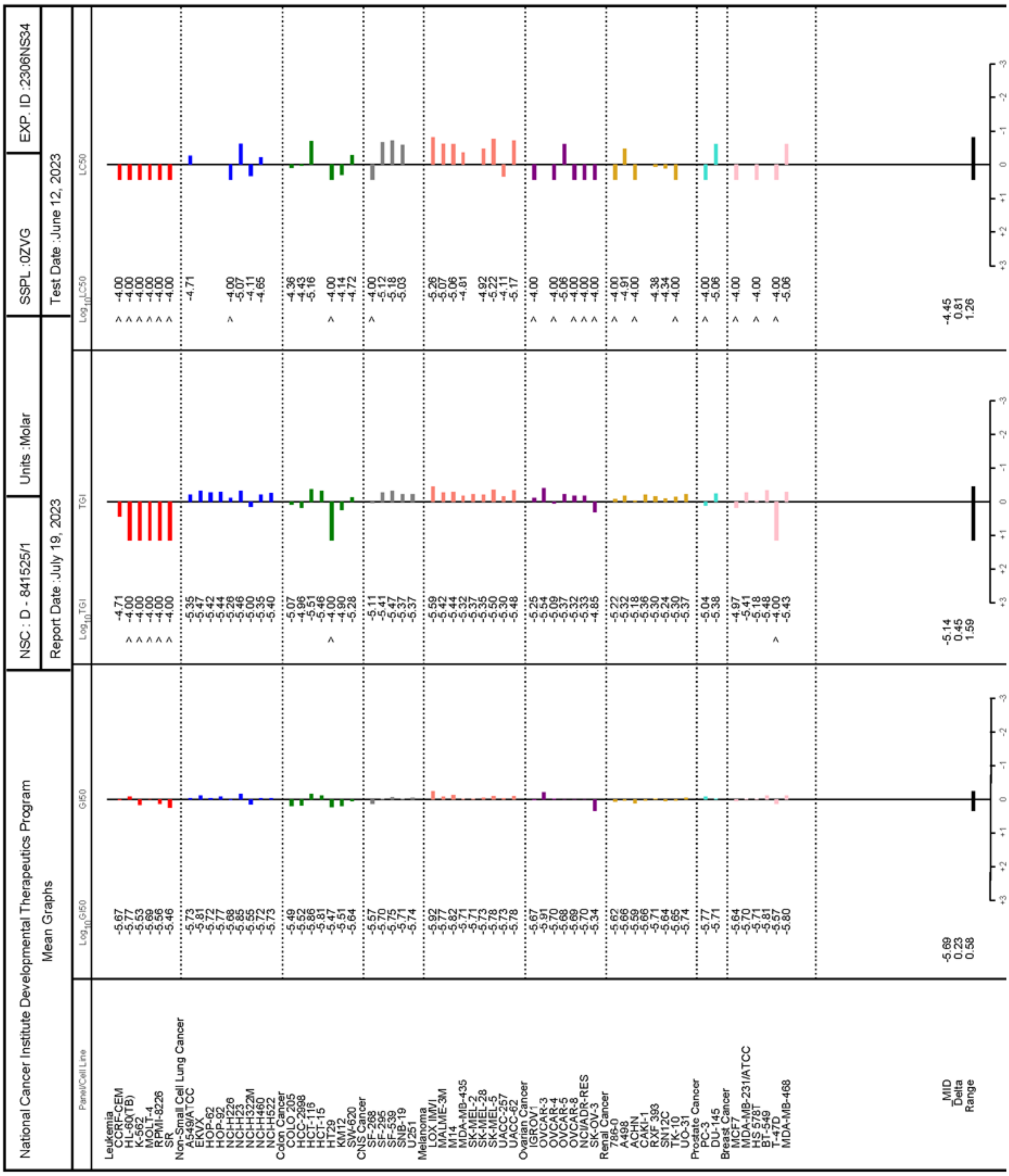

Figure S50. NCI's DTP dose-response report for compound **5**. Mean graphs of GI_{50}, TGI and LC_{50} values.

A)

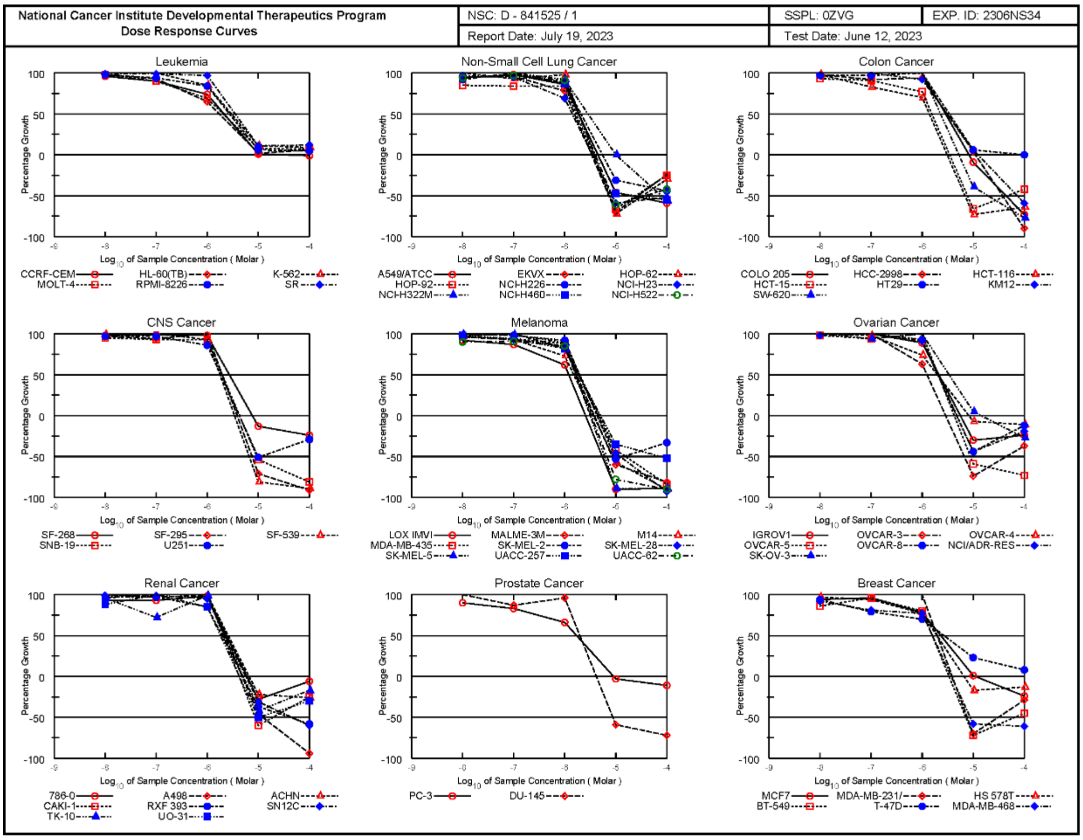

B)

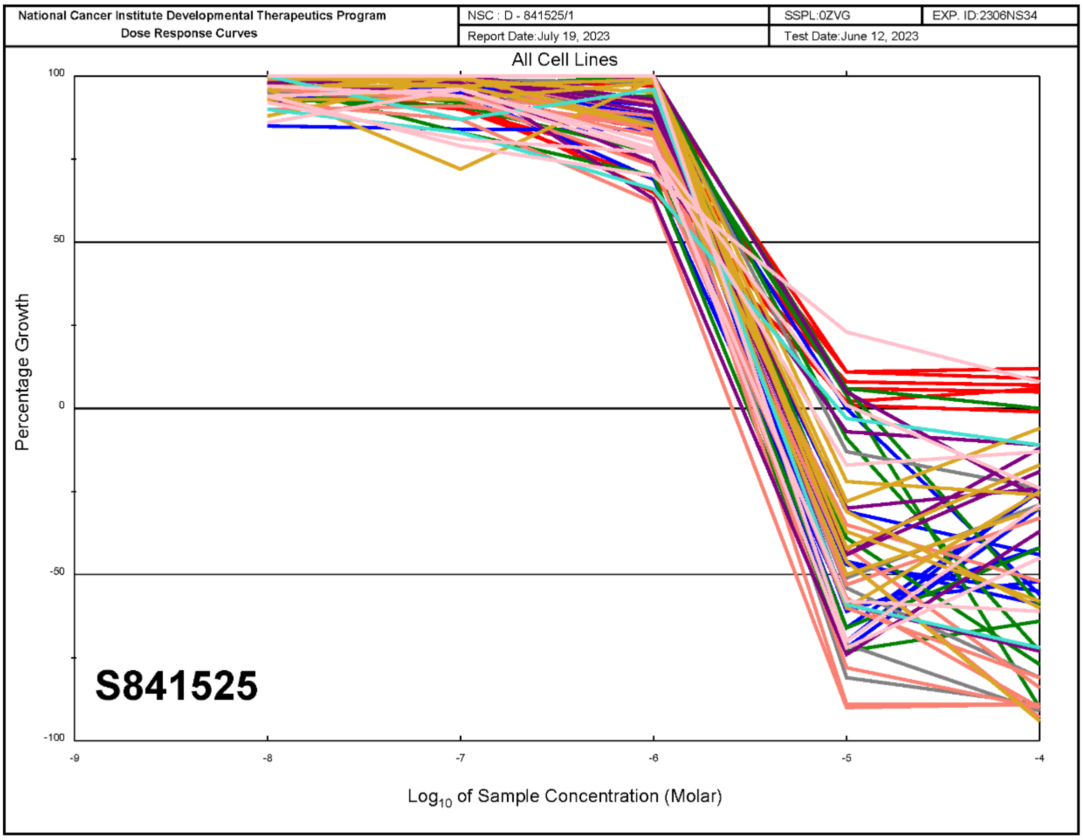

Figure S51. Dose-response curves of the NCI analysis for **5**. (**A**) Curves grouped in nine subpanels derived from different cancer types. (**B**) Curves obtained for all the cancer cell lines tested.

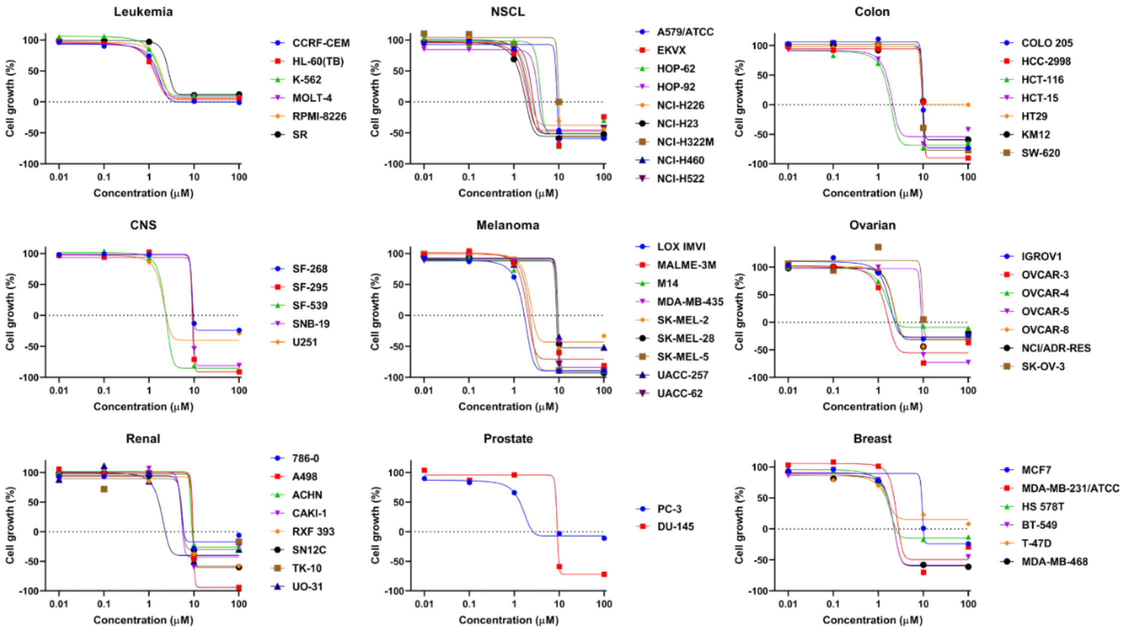

Figure S52. Dose-response curves of **5** in all the cell lines in the NCI panel.

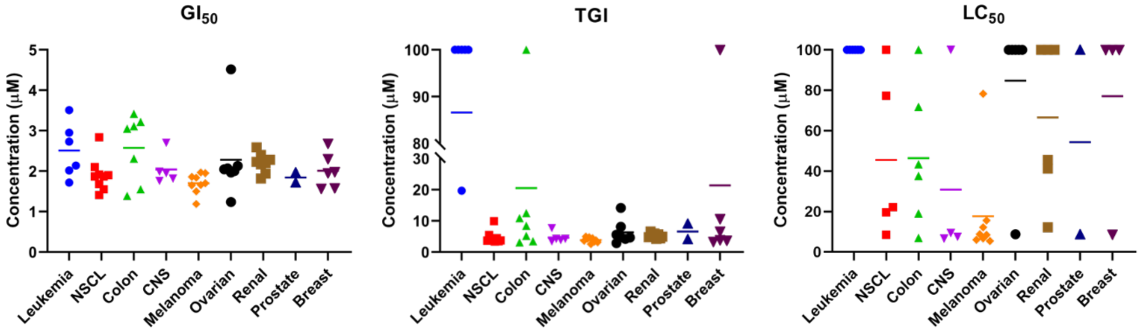

Figure S53. Mean graphs of the GI_{50}, TGI, and LC_{50} values of **5**.

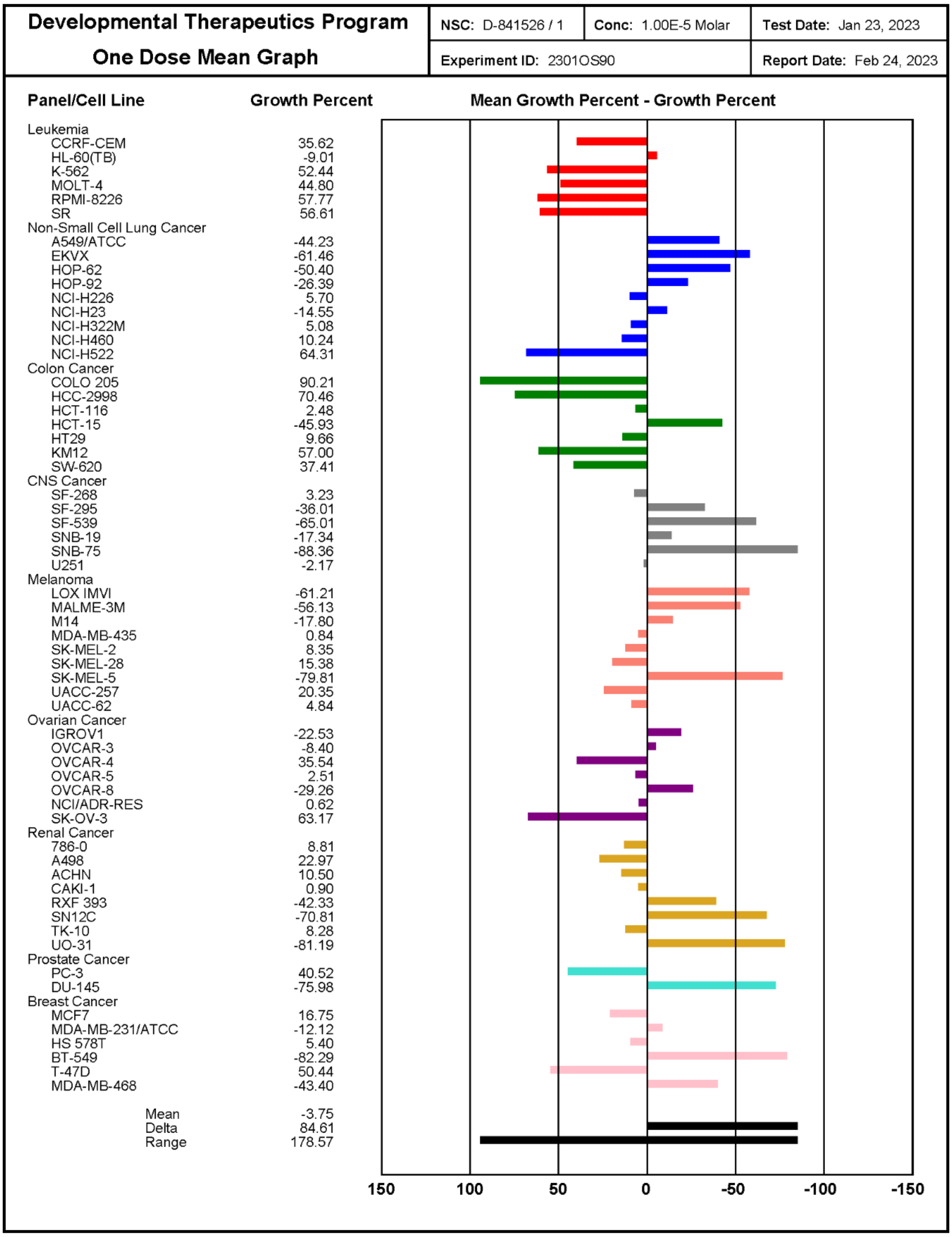

Figure S54. NCI-60 results at one dose (10 μM) of compound **6** (NSC 841526) after 48 h of treatment.

National Cancer Institute Developmental Therapeutics Program
In-Vitro Testing Results

NSC : D - 841526 / 1	Experiment ID : 2306NS37	Test Type : 08	Units : Molar
Report Date : July 28, 2023	Test Date : June 20, 2023	QNS :	MC :
COMI : C5	Stain Reagent : SRB Dual-Pass Related	SSPL : 0ZVG	

			Log10 Concentration												
	Time		Mean Optical Densities					Percent Growth							
Panel/Cell Line	Zero	Ctrl	-8.0	-7.0	-6.0	-5.0	-4.0	-8.0	-7.0	-6.0	-5.0	-4.0	GI50	TGI	LC50
Leukemia															
CCRF-CEM	0.475	1.872	1.885	1.868	1.738	1.000	0.259	101	100	90	38	-45	5.82E-6	2.83E-5	> 1.00E-4
HL-60(TB)	0.528	2.129	1.992	2.021	1.860	0.442	0.356	91	93	83	-16	-33	2.16E-6	6.86E-6	> 1.00E-4
K-562	0.122	0.904	0.767	0.796	0.782	0.421	0.064	82	86	84	38	-48	5.54E-6	2.77E-5	> 1.00E-4
MOLT-4	0.570	2.552	2.570	2.431	2.197	1.382	0.311	101	94	82	41	-46	6.03E-6	2.98E-5	> 1.00E-4
RPMI-8226	0.580	2.300	2.304	2.256	1.939	1.621	0.341	100	97	79	61	-41	1.27E-5	3.93E-5	> 1.00E-4
Non-Small Cell Lung Cancer															
A549/ATCC	0.300	1.846	1.831	1.816	1.676	0.160	0.166	99	98	89	-47	-45	1.94E-6	4.52E-6	> 1.00E-4
EKVX	0.616	2.065	2.024	1.936	1.739	0.284	0.296	97	91	78	-54	-52	1.62E-6	3.89E-6	9.33E-6
HOP-62	0.743	2.284	2.105	2.087	2.261	0.623	0.241	88	87	99	-16	-68	2.65E-6	7.23E-6	4.54E-5
HOP-92	1.120	1.778	1.757	1.772	1.772	1.181	0.492	97	99	99	9	-56	3.52E-6	1.39E-5	8.07E-5
NCI-H226	0.673	1.330	1.400	1.344	1.278	0.652	0.340	111	102	92	-3	-49	2.77E-6	9.26E-6	> 1.00E-4
NCI-H23	0.610	1.842	1.799	1.773	1.754	0.695	0.367	96	94	93	7	-40	3.15E-6	1.40E-5	> 1.00E-4
NCI-H322M	0.732	2.246	2.334	2.291	2.285	1.120	0.547	106	103	103	26	-25	4.82E-6	3.18E-5	> 1.00E-4
NCI-H460	0.237	2.152	2.329	2.337	2.139	0.458	0.183	109	110	99	12	-23	3.65E-6	2.17E-5	> 1.00E-4
NCI-H522	0.702	2.064	2.042	1.998	1.982	1.342	0.092	98	95	94	47	-87	8.63E-6	2.24E-5	5.30E-5
Colon Cancer															
COLO 205	0.489	2.040	2.065	2.154	2.059	1.713	0.087	102	107	101	79	-82	1.51E-5	3.09E-5	6.31E-5
HCC-2998	0.669	2.235	2.109	2.076	2.018	1.725	0.570	92	90	86	67	-15	1.63E-5	6.60E-5	> 1.00E-4
HCT-116	0.233	2.180	2.107	2.003	2.036	0.658	0.090	96	91	93	22	-62	4.00E-6	1.83E-5	7.26E-5
HCT-15	0.260	1.846	1.753	1.734	1.593	0.197	0.039	94	93	84	-24	-85	2.06E-6	5.95E-6	2.64E-5
HT29	0.212	1.437	1.511	1.462	1.405	0.285	0.079	106	102	97	6	-63	3.30E-6	1.22E-5	6.52E-5
KM12	0.453	1.426	1.437	1.489	1.388	0.708	0.100	101	106	96	26	-78	4.56E-6	1.78E-5	5.38E-5
SW-620	0.244	1.768	1.800	1.847	1.778	1.018	0.110	102	105	101	51	-55	1.02E-5	3.02E-5	8.98E-5
CNS Cancer															
SF-268	0.779	2.240	2.237	2.153	2.134	1.268	0.509	100	94	93	33	-35	5.25E-6	3.10E-5	> 1.00E-4
SF-295	0.957	2.778	2.706	2.677	2.771	0.783	0.515	96	94	100	-18	-46	2.64E-6	7.00E-6	> 1.00E-4
SF-539	0.789	2.438	2.563	2.548	2.618	0.456	0.243	108	107	111	-42	-69	2.50E-6	5.30E-6	1.94E-5
SNB-19	0.726	2.616	2.524	2.499	2.500	0.931	0.108	95	94	94	11	-85	3.37E-6	1.30E-5	4.30E-5
SNB-75	1.022	2.166	2.054	2.040	1.953	0.357	0.691	90	89	81	-65	-32	1.64E-6	3.59E-6	.
U251	0.319	1.887	1.812	1.736	1.730	0.445	0.077	95	90	90	8	-76	3.08E-6	1.25E-5	4.90E-5
Melanoma															
LOX IMVI	0.248	1.769	1.761	1.695	1.551	0.023	0.007	99	95	86	-91	-97	1.59E-6	3.06E-6	5.88E-6
MALME-3M	0.517	1.271	1.282	1.360	1.223	0.375	0.178	101	112	94	-27	-66	2.29E-6	5.93E-6	3.89E-5
M14	0.493	2.115	2.077	2.044	2.000	0.603	0.382	98	96	93	7	-23	3.15E-6	1.70E-5	> 1.00E-4
MDA-MB-435	0.588	2.030	2.000	2.036	2.134	0.861	0.131	98	100	107	19	-78	4.45E-6	1.57E-5	5.16E-5
SK-MEL-2	1.303	3.111	3.105	3.108	3.018	2.687	0.649	100	100	95	77	-50	1.62E-5	4.02E-5	9.97E-5
SK-MEL-28	0.607	1.743	1.785	1.702	1.723	0.969	0.329	104	96	98	32	-46	5.32E-6	2.57E-5	> 1.00E-4
SK-MEL-5	0.723	2.543	2.491	2.544	2.343	0.303	0.102	97	100	89	-58	-86	1.84E-6	4.03E-6	8.80E-6
UACC-257	1.026	2.527	2.467	2.451	2.303	1.524	0.561	96	95	85	33	-45	4.74E-6	2.65E-5	> 1.00E-4
Ovarian Cancer															
IGROV1	0.360	1.754	2.033	2.099	1.750	0.566	0.270	120	125	100	15	-25	3.85E-6	2.34E-5	> 1.00E-4
OVCAR-3	0.545	2.263	2.327	2.229	2.197	1.987	0.469	104	98	96	84	-14	2.22E-5	7.19E-5	> 1.00E-4
OVCAR-4	0.758	1.888	1.892	1.816	1.761	1.304	0.628	100	94	89	48	-17	9.10E-6	5.46E-5	> 1.00E-4
OVCAR-5	0.626	1.723	1.760	1.768	1.799	1.106	0.413	103	104	107	44	-34	7.95E-6	3.65E-5	> 1.00E-4
OVCAR-8	0.398	1.971	1.939	1.991	1.837	0.423	0.073	98	101	91	2	-82	2.89E-6	1.04E-5	4.16E-5
NCI/ADR-RES	0.415	1.403	1.425	1.388	1.322	0.337	0.128	102	98	92	-19	-69	2.38E-6	6.75E-6	4.16E-5
SK-OV-3	0.802	1.960	2.146	1.915	2.477	1.386	0.773	116	96	145	50	-4	1.02E-5	8.57E-5	> 1.00E-4
Renal Cancer															
786-0	0.585	2.426	2.361	2.319	2.404	1.358	0.229	96	94	99	42	-61	7.23E-6	2.56E-5	7.83E-5
A498	1.476	2.360	2.369	2.308	2.227	2.011	0.129	101	94	85	60	-91	1.17E-5	2.50E-5	5.34E-5
ACHN	0.438	1.955	1.954	2.030	1.976	0.865	0.146	100	105	101	28	-67	5.03E-6	1.98E-5	6.67E-5
CAKI-1	0.528	2.482	2.436	2.381	2.307	0.991	0.430	98	95	91	24	-19	4.07E-6	3.62E-5	> 1.00E-4
RXF 393	0.851	1.497	1.544	1.503	1.512	1.035	0.200	107	101	102	28	-77	5.12E-6	1.87E-5	5.59E-5
SN12C	0.597	2.529	2.426	2.484	2.387	0.688	0.545	95	98	93	5	-9	3.05E-6	2.24E-5	> 1.00E-4
TK-10	1.236	2.947	2.886	2.883	2.864	1.896	0.443	96	96	95	39	-64	6.27E-6	2.37E-5	7.28E-5
UO-31	0.406	1.507	1.359	1.414	1.241	0.528	0.149	87	92	76	11	-63	2.50E-6	1.41E-5	6.62E-5
Prostate Cancer															
PC-3	0.555	1.941	1.923	1.920	1.789	1.194	0.533	99	98	89	46	-4	8.11E-6	8.33E-5	> 1.00E-4
DU-145	0.333	1.448	1.506	1.470	1.420	0.282	0.181	105	102	97	-15	-46	2.64E-6	7.32E-6	> 1.00E-4
Breast Cancer															
MCF7	0.333	1.784	1.685	1.663	1.480	0.479	0.246	93	92	79	10	-26	2.63E-6	1.89E-5	> 1.00E-4
MDA-MB-231/ATCC	0.687	1.637	1.641	1.669	1.771	0.879	0.334	100	103	114	20	-51	4.82E-6	1.92E-5	9.57E-5
HS 578T	1.315	2.302	2.311	2.215	2.150	1.507	1.159	101	91	85	19	-12	3.39E-6	4.18E-5	> 1.00E-4
BT-549	1.105	2.124	2.002	1.926	1.878	1.295	0.626	88	81	76	19	-43	2.83E-6	2.00E-5	> 1.00E-4
MDA-MB-468	0.681	1.046	1.028	1.035	1.001	0.185	0.199	95	97	88	-73	-71	1.71E-6	3.51E-6	7.20E-6

Figure S55. NCI's DTP dose-response report for compound **6**. GI_{50}, TGI and LC_{50} values (expressed as molarity).

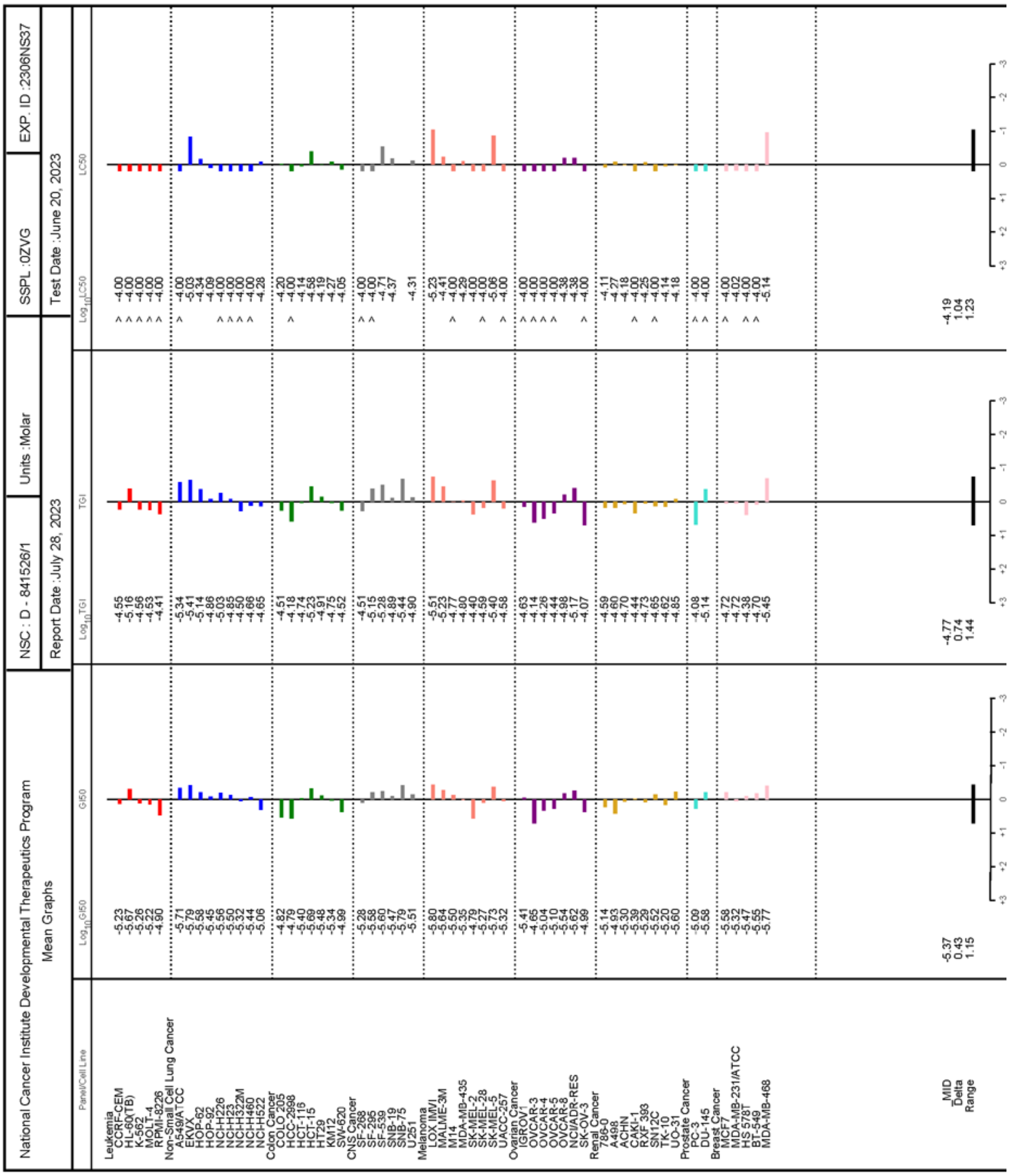

Figure S56. NCI's DTP dose-response report for compound **6**. Mean graphs of GI_{50}, TGI and LC_{50} values.

A)

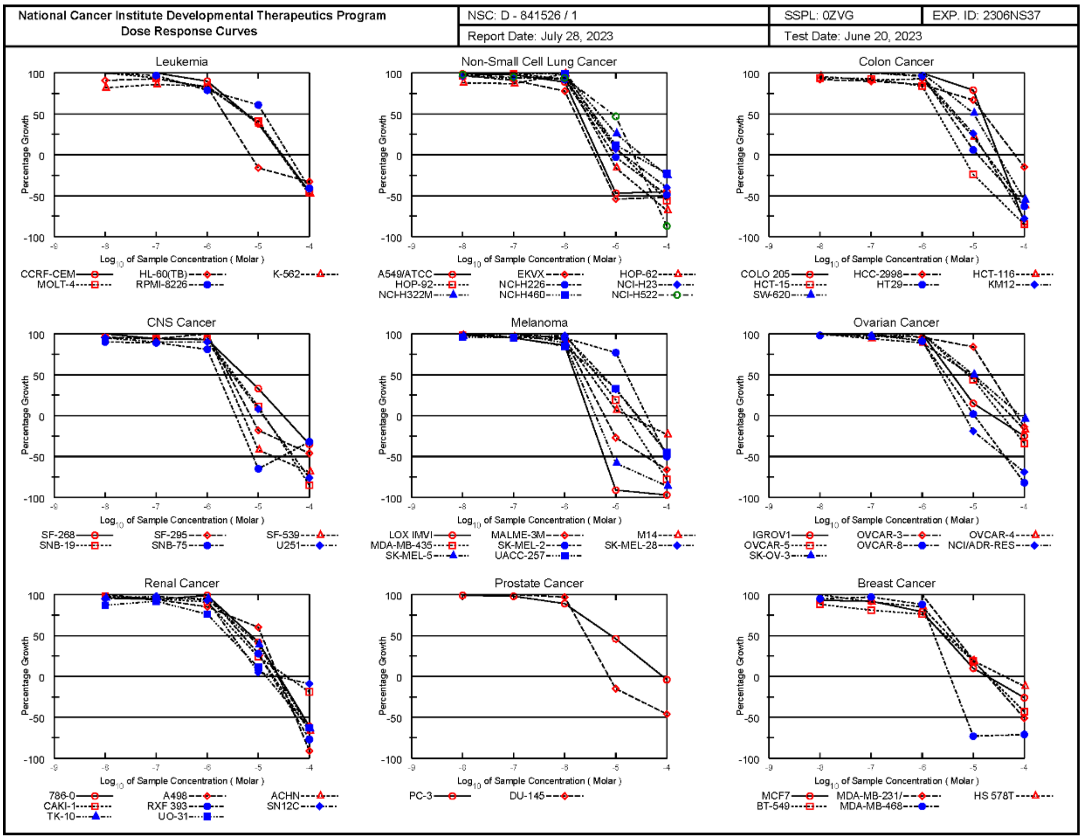

B)

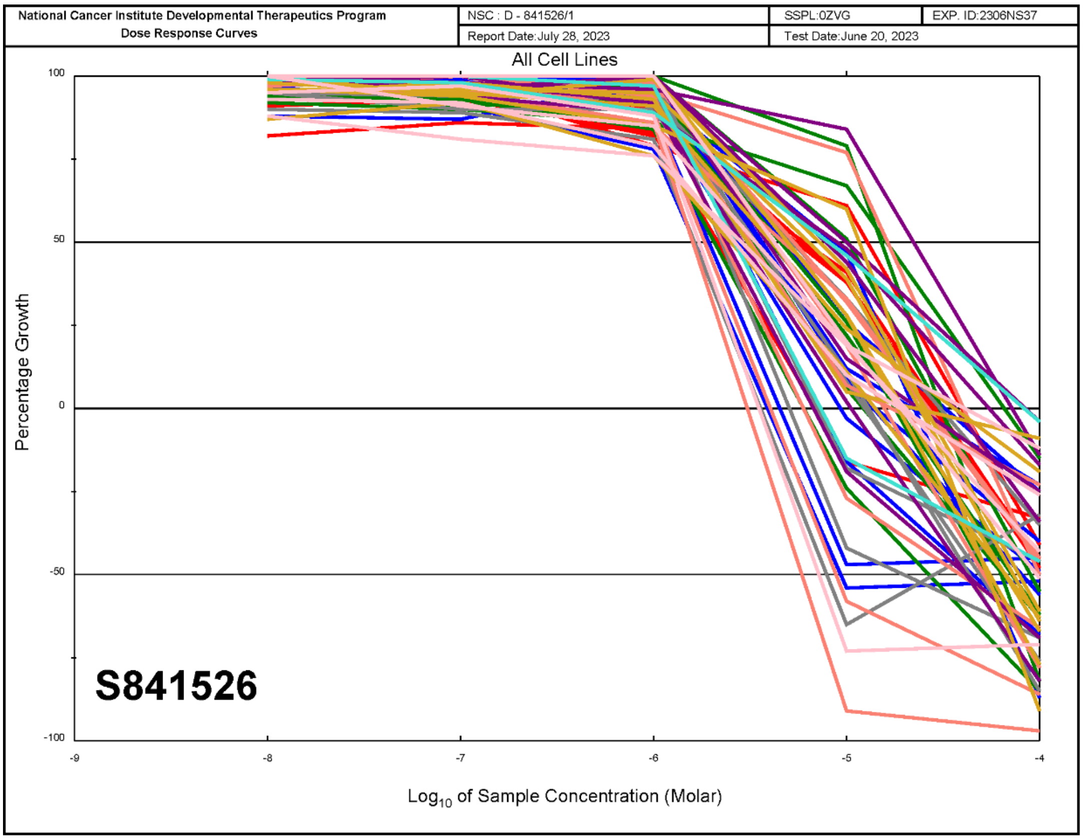

Figure S57. Dose-response curves of the NCI analysis for **6**. (**A**) Curves grouped in nine subpanels derived from different cancer types. (**B**) Curves obtained for all the cancer cell lines tested.

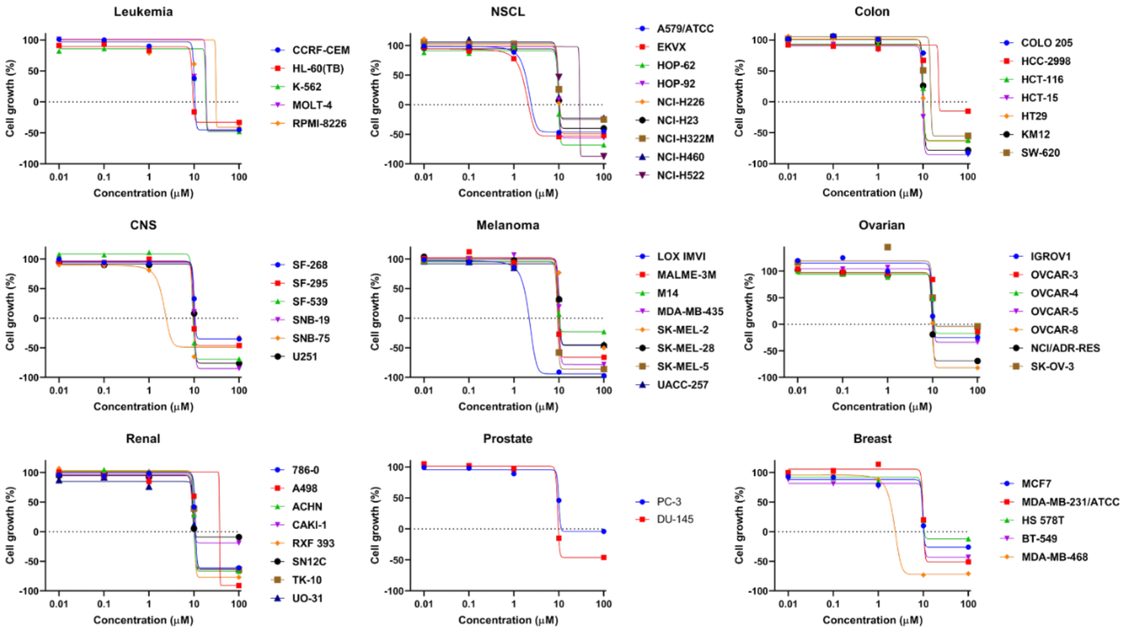

Figure S58. Dose-response curves of **6** in all the cell lines in the NCI panel.

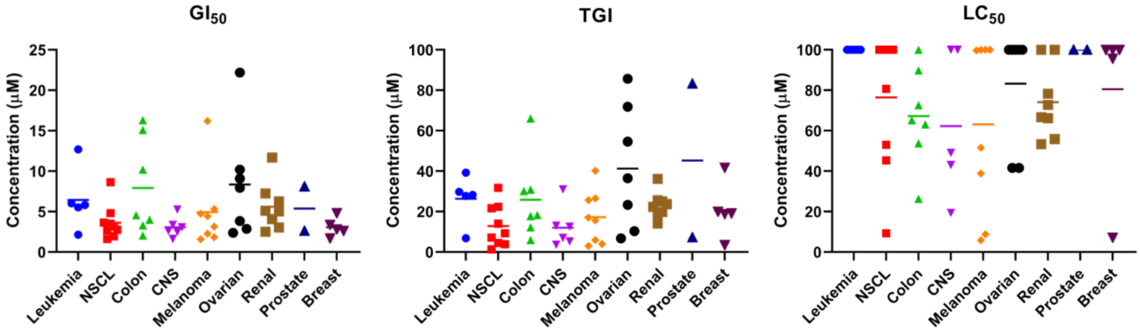

Figure S59. Mean graphs of the GI_{50}, TGI, and LC_{50} values of **6**.

Table S1. Pharmacokinetic properties obtained for compounds **1, 2,** and **3**. Values obtained from ADMElab 3.0.

	Property	A	B	C
0Absorption	Caco-2 permeability (log cm/s)	Poor (-5.21)	Poor (-5.16)	Good (-4.96)
	P-gp substrate	Yes	Yes	Yes
	Human intestinal absorption (HIA)	Yes	Yes	Yes
Distribution	Plasma protein binding (PPB)	Poor (99.02%)	Poor (97.26 %)	Poor (98.32%)
	Volume of distribution at steady state (L/kg)	Excellent (0.52)	Excellent (0.34)	Excellent (0.16)
	Fraction unbound in plasms (Fu)	Low (0.68)	Low (2.26)	Low (0.78)
Metabolism	CYP2D6 substrate	Yes	No	Yes
	CYP1A2 substrate	Yes	No	Yes
	CYP2C19 inhibitor	No	No	No
	CYP2C9 inhibitor	No	No	No
	CYP3A4 inhibitor	No	No	Yes
	CYP2B6 inhibitor	No	No	No
	CYP2C8 inhibitor	No	No	No
Excretion	Plasma clearance (CL_{plasma}) (ml/min/kg)	Medium (5.56)	Medium (5.86)	Medium (6.27)
	Half-life ($T_{1/2}$) of a drug (h)	Short life (0.85)	Short life (0.59)	Short life (0.30)
Toxicity	Human hepatotoxicity	Non-hepatotoxic	Non-hepatotoxic	Non-hepatotoxic
	Skin sensitisation	Yes	Yes	Yes
	AMES toxicity	No	No	No
Physicochemica l properties	Molecular weight (g/mol)	411.99	431.98	402.99
	Number of hydrogen bond acceptors (nHA)	4	6	4
	Number of hydrogen bond donors (nHD)	1	1	1
	Topological surface area (TPSA) (Å^2)	69.96	89.31	55.40
Medicinal chemistry	Lipinski rule [a]	Accepted	Accepted	Accepted
	Synthetic accessibility (SA) score [b]	Easy	Easy	Easy
	PAINS (alerts)	0	0	0

[a] MW ≤ 500, logP ≤ 5, nHA ≤ 10, nHD ≤ 5; [b] high SAscore: ≥ 6, difficult to synthesize, low SAscore, < 6, easy to synthesize.

Molecule 1

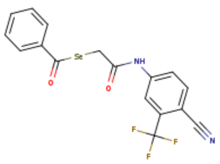

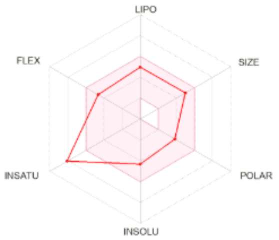

SMILES N#Cc1ccc(cc1C(F)(F)F)NC(=O)C[Se]C(=O)c1ccccc1

Physicochemical Properties	
Formula	C17H11F3N2O2Se
Molecular weight	411.24 g/mol
Num. heavy atoms	25
Num. arom. heavy atoms	12
Fraction Csp3	0.12
Num. rotatable bonds	7
Num. H-bond acceptors	6
Num. H-bond donors	1
Molar Refractivity	85.94
TPSA	69.96 Å²

Lipophilicity	
Log $P_{o/w}$ (iLOGP)	0.00
Log $P_{o/w}$ (XLOGP3)	3.21
Log $P_{o/w}$ (WLOGP)	4.44
Log $P_{o/w}$ (MLOGP)	2.49
Log $P_{o/w}$ (SILICOS-IT)	2.99
Consensus Log $P_{o/w}$	2.63

Water Solubility	
Log *S* (ESOL)	-4.31
Solubility	2.04e-02 mg/ml ; 4.95e-05 mol/l
Class	Moderately soluble
Log *S* (Ali)	-4.35
Solubility	1.83e-02 mg/ml ; 4.45e-05 mol/l
Class	Moderately soluble
Log *S* (SILICOS-IT)	-6.42
Solubility	1.55e-04 mg/ml ; 3.76e-07 mol/l
Class	Poorly soluble

Pharmacokinetics	
GI absorption	High
BBB permeant	Yes
P-gp substrate	No
CYP1A2 inhibitor	Yes
CYP2C19 inhibitor	Yes
CYP2C9 inhibitor	No
CYP2D6 inhibitor	Yes
CYP3A4 inhibitor	Yes
Log K_p (skin permeation)	-6.53 cm/s

Druglikeness	
Lipinski	Yes; 0 violation
Ghose	Yes
Veber	Yes
Egan	Yes
Muegge	Yes
Bioavailability Score	0.55

Medicinal Chemistry	
PAINS	0 alert
Brenk	1 alert: heavy_metal
Leadlikeness	No; 1 violation: MW>350
Synthetic accessibility	3.03

Figure S60. Pharmacokinetic properties obtained for **1.** Values obtained from SwissADME.

Molecule 1

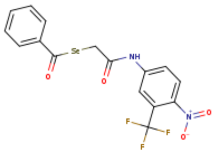

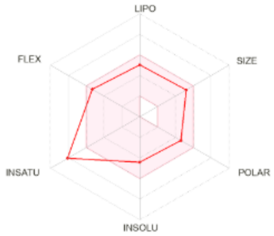

SMILES O=C(Nc1ccc(c(c1)C(F)(F)F)[N+](=O)[O-])C[Se]C(=O)c1ccccc1

Physicochemical Properties	
Formula	C16H11F3N2O4Se
Molecular weight	431.22 g/mol
Num. heavy atoms	26
Num. arom. heavy atoms	12
Fraction Csp3	0.12
Num. rotatable bonds	8
Num. H-bond acceptors	7
Num. H-bond donors	1
Molar Refractivity	90.05
TPSA	91.99 Å²

Lipophilicity	
Log $P_{o/w}$ (iLOGP)	0.00
Log $P_{o/w}$ (XLOGP3)	3.32
Log $P_{o/w}$ (WLOGP)	4.48
Log $P_{o/w}$ (MLOGP)	2.17
Log $P_{o/w}$ (SILICOS-IT)	0.83
Consensus Log $P_{o/w}$	2.16

Water Solubility	
Log *S* (ESOL)	-4.42
Solubility	1.64e-02 mg/ml ; 3.81e-05 mol/l
Class	Moderately soluble
Log *S* (Ali)	-4.93
Solubility	5.09e-03 mg/ml ; 1.18e-05 mol/l
Class	Moderately soluble
Log *S* (SILICOS-IT)	-5.70
Solubility	8.60e-04 mg/ml ; 2.00e-06 mol/l
Class	Moderately soluble

Pharmacokinetics	
GI absorption	High
BBB permeant	No
P-gp substrate	Yes
CYP1A2 inhibitor	No
CYP2C19 inhibitor	Yes
CYP2C9 inhibitor	Yes
CYP2D6 inhibitor	Yes
CYP3A4 inhibitor	Yes
Log K_p (skin permeation)	-6.57 cm/s

Druglikeness	
Lipinski	Yes; 0 violation
Ghose	Yes
Veber	Yes
Egan	Yes
Muegge	Yes
Bioavailability Score	0.55

Medicinal Chemistry	
PAINS	0 alert
Brenk	3 alerts: heavy_metal, nitro_group, oxygen-nitrogen_single_bond
Leadlikeness	No; 2 violations: MW>350, Rotors>7
Synthetic accessibility	3.04

Figure S61. Pharmacokinetic properties obtained for **2.** Values obtained from SwissADME.

Molecule 1

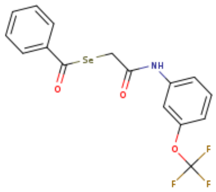

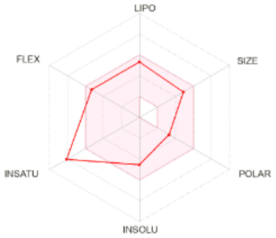

SMILES O=C(Nc1cccc(c1)OC(F)(F)F)C[Se]C(=O)c1ccccc1

Physicochemical Properties	
Formula	C16H12F3NO3Se
Molecular weight	402.23 g/mol
Num. heavy atoms	24
Num. arom. heavy atoms	12
Fraction Csp3	0.12
Num. rotatable bonds	8
Num. H-bond acceptors	6
Num. H-bond donors	1
Molar Refractivity	82.91
TPSA	55.40 Å²

Lipophilicity	
Log $P_{o/w}$ (iLOGP)	0.00
Log $P_{o/w}$ (XLOGP3)	3.79
Log $P_{o/w}$ (WLOGP)	4.56
Log $P_{o/w}$ (MLOGP)	2.33
Log $P_{o/w}$ (SILICOS-IT)	2.49
Consensus Log $P_{o/w}$	2.63

Water Solubility	
Log *S* (ESOL)	-4.56
Solubility	1.10e-02 mg/ml ; 2.73e-05 mol/l
Class	Moderately soluble
Log *S* (Ali)	-4.65
Solubility	9.06e-03 mg/ml ; 2.25e-05 mol/l
Class	Moderately soluble
Log *S* (SILICOS-IT)	-6.09
Solubility	3.28e-04 mg/ml ; 8.17e-07 mol/l
Class	Poorly soluble

Pharmacokinetics	
GI absorption	High
BBB permeant	Yes
P-gp substrate	No
CYP1A2 inhibitor	Yes
CYP2C19 inhibitor	Yes
CYP2C9 inhibitor	Yes
CYP2D6 inhibitor	Yes
CYP3A4 inhibitor	Yes
Log K_p (skin permeation)	-6.06 cm/s

Druglikeness	
Lipinski	Yes; 0 violation
Ghose	Yes
Veber	Yes
Egan	Yes
Muegge	Yes
Bioavailability Score	0.55

Medicinal Chemistry	
PAINS	0 alert
Brenk	1 alert: heavy_metal
Leadlikeness	No; 3 violations: MW>350, Rotors>7, XLOGP3>3.5
Synthetic accessibility	2.82

Figure S62. Pharmacokinetic properties obtained for **3.** Values obtained from SwissADME.
